# Umsetzbarkeit des interdisziplinären multimodalen Assessments – die Teamperspektive

**DOI:** 10.1007/s00482-024-00796-z

**Published:** 2024-04-09

**Authors:** Leonie Schouten, Frank Petzke, Anne Gärtner, Bernd Nagel, PAIN2020-Team, Thomas Isenberg, Gabriele Lindena, Carolin Martin, André Möller, Katharina Augustin, Ulrike Kaiser, Anne Gärtner, Anke Preißler, Greta Hoffmann, Julia Pritzke Michael, Frank Petzke, Michael Pfingsten, Leonie Schouten, Karin Deppe, Hans-Raimund Casser, Bernd Nagel, Katja Schwenk, Beatrice Metz-Oster, Lena Milch, Jana Rensland, Thomas Kohlmann, Daniel Szczotkowski, Ursula Marschall, Catharina Schumacher, Ulrike Kaiser

**Affiliations:** 1 Schmerzmedizin, Klinik für Anästhesiologie, Universitätsmedizin Göttingen, Robert-Koch-Straße 40, 37075 Göttingen, Deutschland; 2 Medizinische Fakultät und UniversitätsSchmerzCentrum, Universitätsklinik Carl Gustav Carus Dresden, Fetscherstraße 74, 01307 Dresden, Deutschland; 3 Ambulanz, Tagesklinik, Stationäre Behandlung, DRK Schmerz-Zentrum Mainz, Auf der Steig 16, 55131 Mainz, Deutschland; 4 Klinik für Anästhesiologie und Intensivmedizin, Universitätsklinikum Schleswig-Holstein, Ratzeburger Allee 160, 23538 Lübeck, Deutschland

**Keywords:** Schmerzen und Risikofaktoren, Interdisziplinäre multimodale Schmerztherapie, Ambulante Diagnostik, Versorgungsforschung, Teamprozess, Pain and risk factors, Interdisciplinary multimodal pain therapy, Outpatient diagnostics, Health services research, Team process

## Abstract

**Hintergrund:**

Sekundärpräventive, ambulante Diagnostikangebote für Patient:innen mit Schmerzen und Risikofaktoren für eine Chronifizierung sind bisher nicht hinreichend etabliert. Im Projekt PAIN2020 (Innovationsfonds, 01NVF17049) wurde erstmalig ein frühzeitig im Krankheitsverlauf ansetzendes, ambulantes interdisziplinäres multimodales Assessment (IMA) eingeführt.

**Ziel:**

Zur Durchführung des IMA wurden Abläufe zur Teamzusammenarbeit und Entscheidungskriterien entwickelt, die durch ein Team aus medizinischen, physiotherapeutischen und psychologischen Therapeut:innen umgesetzt wurden. Diese Abläufe und Entscheidungskriterien sollen vor dem Hintergrund klinischer Erfahrung diskutiert und hinsichtlich ihrer Umsetzbarkeit (qualitativ) überprüft werden.

**Methodik:**

Im September 2021 fand ein Workshop zum IMA in PAIN2020 statt, um die im Prozess bisher gewonnenen Erkenntnisse und Erfahrungen durch das Monitoring und die strukturierende Dokumentation in der Umsetzung mit Mitarbeitenden bzw. Teams der PAIN2020-Zentren zur Umsetzbarkeit eines strukturierten interdisziplinären multimodalen Assessments gemeinsam zu reflektieren. In drei Arbeitsphasen wurden berufsgruppenspezifische und -übergreifende Themen bearbeitet.

**Ergebnisse:**

In den Entscheidungsprozessen der Berufsgruppen zeigen sich neben professionsspezifischen Schwerpunkten im Rahmen der Befunderhebung (somatische, funktionelle oder psychosoziale Kernkriterien) jeweils übergreifende Kernkriterien innerhalb der Professionen sowie ergänzend patientenbezogene Aspekte, die in den integrativen Teamprozess einbezogen werden. Hinsichtlich der Teamzusammenarbeit lassen sich aus der Umsetzung der Teamsitzung und des Abschlussgesprächs fördernde bzw. hemmende Struktur- und Prozessparameter in der Umsetzung identifizieren, die auch durch interaktionelle Faktoren begleitet werden.

**Diskussion:**

Für die Umsetzung des IMA ergaben sich (1) Anpassungen für das IMA, das derzeit als A‑IMA im Selektivvertrag mit der BARMER umgesetzt wird, und (2) neue Dimensionen bzw. Aufgabenfelder und Ideen für evidenzbasierte Konzepte zur inhaltlichen Ausgestaltung integrativer Diagnostik sowie für die Rückmeldung der Ergebnisse an die Patient:innen, die zukünftig diskutiert werden sollten.

**Graphic abstract:**

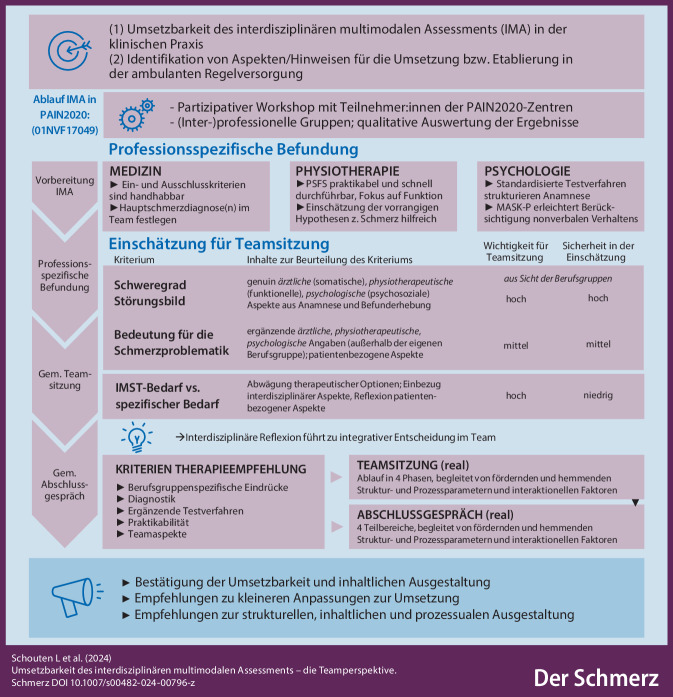

**Zusatzmaterial online:**

Die Online-Version dieses Beitrags (10.1007/s00482-024-00796-z) enthält Zusatzmaterialien zum Projekt PAIN2020.

## Abstract (en)

**Background:**

Secondary preventive outpatient diagnostic services for patients with pain and risk factors for chronification have not yet been sufficiently established. In the PAIN2020 project (Innovation Fund, 01NVF17049) an outpatient interdisciplinary multimodal assessment (IMA) was introduced for the first time early in the course of the disease.

**Objective:**

For the implementation of the IMA procedures for team cooperation and decision criteria were developed, which were implemented by a team of medical, physiotherapeutic and psychological therapists. These procedures and decision criteria are to be discussed against the background of clinical experience and examined with respect to their feasibility (qualitative).

**Methods:**

In PAIN2020 a workshop on IMA was held in September 2021 to jointly reflect on the findings and experiences gained in the process so far through monitoring and structuring documentation in the implementation with staff or teams of PAIN2020 centers on the feasibility of implementing a structured interdisciplinary multimodal assessment. In three work phases, occupational group-specific and cross-occupational group topics were addressed.

**Results:**

In the decision-making processes of the occupational groups, in addition to profession-specific focal points within the framework of the assessment of findings (somatic, functional or psychosocial core criteria), overarching core criteria within the professions as well as complementary patient-related aspects are evident, which are included in the integrative team process. With respect to team collaboration, the implementation of the team meeting and the final discussion can be used to identify structural and process parameters that promote or inhibit implementation, which are also accompanied by interactional factors.

**Discussion:**

For the implementation of the IMA, there were (1) adaptations of the IMA, which is currently implemented as A‑IMA in the selective agreement with BARMER and (2) new dimensions or task fields and ideas for evidence-based concepts for the content design of integrative diagnostics as well as for the feedback of the results to the patients, which should be discussed in the future.

## Einleitung

Die frühzeitige (ambulante) Diagnostik und Therapie von Patient:innen mit wiederkehrenden Schmerzen und Risikofaktoren für eine Chronifizierung ist bisher in der Versorgung nicht abgebildet [9, 10]. Mit dem Projekt PAIN2020 der Deutschen Schmerzgesellschaft e. V., (Patientenorientiert. Abgestuft. Interdisziplinär. Netzwerk.; 01NVF17049, Laufzeit: 04/2018 bis 03/2022) – gefördert durch den Innovationsfonds und in Zusammenarbeit mit schmerzspezialisierten Einrichtungen der Versorgung und der BARMER – wurde als neue Versorgungsform ein interdisziplinäres multimodales Assessment (IMA) frühzeitig im Krankheitsverlauf bei Patient:innen mit Risikofaktoren für eine Chronifizierung im Vergleich zu einem ärztlichen Assessment in der schmerztherapeutischen Regelversorgung (SRV) untersucht. Ziel ist die Vermeidung einer Chronifizierung im Sinne einer Sekundärprävention [11]. Im IMA arbeiten die Berufsgruppen der Schmerzmedizin, Physiotherapie und Psychologie/Psychotherapie im interdisziplinären Team eng zusammen [11]. Koordiniert wird diese Zusammenarbeit durch pflegerische oder dokumentarische Fachangehörige.

**Abb. 1:**
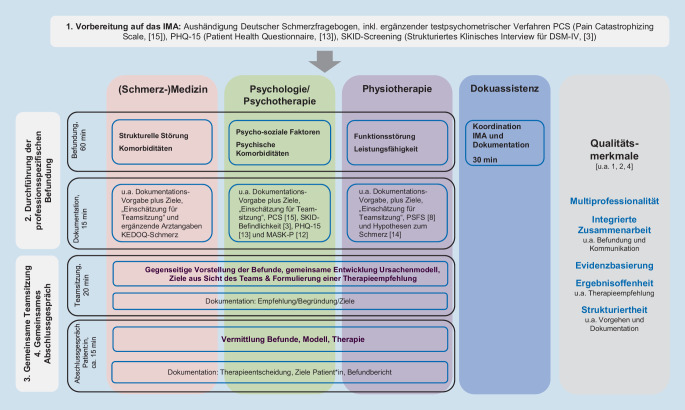
Das interdisziplinäre multimodale Assessment in PAIN2020 – Ablauf, Inhalte und Qualitätsmerkmale

Die Konzeption der neuen Versorgungsleistung IMA orientierte sich an den Empfehlungen und grundsätzlichen Qualitätsmerkmalen zur Umsetzung interdisziplinärer Diagnostik- und Therapieansätze für Patient:innen mit chronischen Schmerzen [1, 2, 4, 18]. Die bereits existierenden Empfehlungen wurden im Rahmen der Konzeption der neuen Versorgungsform an die Zielgruppe der nicht-chronischen Patient:innen angepasst und um ein vorgelagertes Screening, ein gemeinsames, regulär geplantes Abschlussgespräch sowie eine spezifisch an die Durchführung des IMA angepasste, standardisierte Dokumentation ergänzt. Der zeitliche Aufwand wurde für alle Berufsgruppen reduziert. Zum Zeitpunkt der Konzeption wurde das IMA in vier Abschnitte unterteilt (vgl. Abb. 1), für die jeweils ein weitgehend standardisiertes Vorgehen erarbeitet wurde: 1. Vorbereitung auf das IMA (inkl. testpsychometrischer Verfahren, vgl. Tab. 1), 2. Durchführung der professionsspezifischen Befundung im Rahmen des IMA (u. a. inkl. Einordnung in Klassifikationssysteme sowie grundlegender Einschätzungen, vgl. Tab. 1), 3. gemeinsame Teamsitzung und 4. gemeinsames Abschlussgespräch [11].

**Tab. 1:** Verwendete (testpsychometrische) Verfahren und Klassifikationssysteme im IMA nach Zeitpunkt und Berufsgruppe

Zeitpunkt	Berufsgruppe	(Testpsychometrische) Verfahren und Klassifikationssysteme im IMA	Kurzbeschreibung				
Vor dem IMA (Phase 1)	Medizin, Physiotherapie und Psychologie	Deutscher Schmerzfragebogen (DSF; [17])	Mit dem DSF kann eine standardisierte Schmerzdokumentation erfolgen. Er enthält u. a. folgende testpsychometrische Verfahren: DASS [Depression, Anxiety and Stress Scale], VR-12 [Verfahren zur Erfassung gesundheitsbezogener Lebensqualität], FW‑7 [Marburger Fragebogen zum habituellen Wohlbefinden] und SBL‑A [Schmerz-Beschreibungs-Liste]				
	Psychologie Vgl. Online-Zusatzmaterial 6, Dokumente *B13, C9*	Pain Catastrophizing Scale (PCS; [15])	Mit der PCS werden katastrophisierende und ängstliche Kognitionen in Bezug auf das Erleben von Schmerz gemessen. Auf drei Skalen werden insgesamt 13 Items erhoben: Hilflosigkeit (1–5) – hilfloses, ohnmächtiges Erleben, „magnification“ (6, 7, 13) – Erleben von Bedrohung durch Schmerz und Rumination (8–12) – gedankliche Schmerzfixierung				
		SKID-Screening (Strukturiertes Klinisches Interview für DSM-IV; [3])	Das SKID-Screening für DSM-IV ist ein verbreitetes Verfahren zur Diagnostik psychischer Störungen nach dem Klassifikationssystem der Psychiatrie DSM-IV und soll mögliche psychiatrische Komorbiditäten screenen/identifizieren. Es beinhaltet 15 Fragen zu den Bereichen Substanzmissbrauch bzw. -abhängigkeit (1–3), Angststörungen (4–8), Zwangsstörungen (9, 10), Essstörungen (11, 12), somatoforme Störung (13) oder Belastungs- und Anpassungsstörung (14, 15)				
		PHQ-15 (Patient Health Questionnaire; [13])	Der PHQ-15 ist ein Modul des Gesundheitsfragebogens für Patient:innen und wird dazu eingesetzt, den Schweregrad somatischer Symptome zu erfassen. Er erfragt 15 somatische Symptome, die den häufigsten körperlichen Symptomen ambulanter Patient:innen sowie den wichtigsten DSM-IV-Kriterien der Somatisierungsstörung entsprechen				
Während des IMA (Phase 2)	Medizin Vgl. Online-Zusatzmaterial 6, Dokument *C5*	MPSS (Mainz Pain Staging System; [7])	Mit dem Mainzer Stadienmodell der Schmerzchronifizierung wird das aktuelle Chronifizierungsstadium der Patient:innen eingeschätzt. Ergänzend soll das MPSS zur Prognoseeinschätzung und zur Steuerung des Ressourceneinsatzes bei Therapiebeginn dienen. In PAIN2020 ist das MPSS in die Ergänzenden Arztangaben KEDOQ-Schmerz integriert (u. a. werden hier Hauptschmerzdiagnose und -lokalisation ebenfalls dokumentiert)				
	Physiotherapie Vgl. Online-Zusatzmaterial 6, Dokumente *C7, C8*	PSFS (Patientenspezifische Funktionsskala; [8])	Die PSFS ist ein patientenorientiertes Messinstrument und dient der Messung der subjektiven Einschätzung von bis zu 3 individuell gewählten Aktivitäten, die auf einer Skala von 0 bis 10 (0 = unfähig, die Aktivität auszuführen, 10 = kann die Aktivität auf dem gleichen Niveau wie vor der Verletzung oder dem Problem ausführen) eingeschätzt werden				
		Einschätzung der vorrangigen Hypothesen zum Schmerz [14]	Die Einschätzung zu den vorrangigen Hypothesen zum Schmerz erfolgt im Anschluss an Anamnese und Befunderhebung und ist eine subjektive Einschätzung der Physiotherapeut:innen zu den vorrangigen Schmerzmechanismen – unterteilt nach Input‑, Verarbeitungs- und Outputebene –, die sich aus dem Clinical-reasoning-Prozess ergibt				
	Psychologie Vgl. Online-Zusatzmaterial 6, Dokument *C11*	MASK‑P (Multiaxial Schmerzklassifikation; [12])	Die MASK‑P ist ein halbstrukturiertes Klassifikationssystem zur Diagnostik psychosozialer Dimensionen chronischer Schmerzen. In PAIN2020 wurden die ersten 8 Achsen verwendet. Die einzelnen Achsen beschreiben die übergeordnete Richtung der Schmerzverarbeitung sowie differenzierte psychosoziale Faktoren der Schmerzproblematik				
	Alle Berufsgruppen – „fachspezifische Einschätzung für die Teamsitzung“ Medizin: somatisch Physiotherapie: funktionell Psychologie: psychosozial Vgl. Online-Zusatzmaterial 6, Dokumente *C4, C8, C11*	*Schweregrad Störungsbild*	Schweregrad Störungsbild	Bedeutung für Schmerzproblematik	IMST-Ansatz erforderlich	Spezifische Behandlung erforderlich	
		*Bedeutung für die Schmerzproblematik*					
		*IMST-Ansatz erforderlich*	_____	_____	_____	_____	
		*Spezifische Behandlung in Fachbereich erforderlich*	0 = kein 1 = leicht 2 = mittel 3 = schwer	0 = keine 1 = gering 2 = moderat 3 = hoch	0 = nein 1 = fraglich 2 = ja	0 = nein 1 = fraglich 2 = ja	

Ziel dieses standardisierten Vorgehens war es, eine einheitliche Vorgehensweise innerhalb der teilnehmenden Einrichtungen (*n* = 28) zu gewährleisten und den Fokus eines jeden IMA auf die Befunderhebung bzgl. einer möglichen Schmerzchronifizierung und entsprechende Empfehlungen zu legen.

In PAIN2020 wurden im Rahmen eines umfassenden Monitoringkonzepts Struktur- und Prozessparameter zur Durchführung des IMA erhoben [11]. Diese dienten vorrangig der Prüfung und Sicherstellung der Durchführungsqualität entsprechend dem Studienprotokoll und der Betreuung der Einrichtungen bei der Durchführung als Indikatoren für Rückmeldungen vonseiten des Projektteams und später deskriptiven Analysen zur professionsspezifischen und teambezogenen Umsetzbarkeit des IMA [6, 19, 20]. Mit diesen Erkenntnissen und den Erfahrungen ergaben sich Fragestellungen zur Umsetzbarkeit des IMA im klinischen Alltag (u. a. Durchführbarkeit IMA inkl. hinsichtlich bestehender Struktur- und Prozessparameter, Umsetzung, Handhabung bzw. Praktikabilität angewandter Testverfahren, Erfahrungen in der Teamzusammenarbeit), die aus Sicht der Autor:innen mit den Durchführenden des IMA im Rahmen eines 1‑tägigen qualitativen Workshops gemeinsam reflektiert und beantwortet werden sollten^1^.

Diese Studie verfolgte entsprechend zwei Fragestellungen:

1. Ist das IMA aus klinischer Perspektive umsetzbar?
2. Welche konkreten Aspekte sollten für eine Umsetzung in der späteren Regelversorgung besonders berücksichtigt werden, um solch ein diagnostisches Angebot langfristig in der ambulanten Versorgung zu etablieren?

## Methoden

Es wurde ein qualitatives Studiendesign in Anlehnung an die Vorstufen der Partizipation (5. Stufe) des Stufenmodells zur Partizipation für Gesundheitsförderung von [22] ausgewählt, um die Erfahrungen der durchführenden Therapeut:innen im IMA zu erfassen. Durch die partizipative Herangehensweise wird eine gemeinsame Gestaltung des Untersuchungsprozesses ermöglicht, in welchem Teilnehmende (TN) als Beratende im Sinne der informierten Mitsprache fungieren [5]. Als Erhebungsmethode wurde das Format eines 1‑tägigen moderierten und strukturierten Workshops gewählt. Dieser fand interprofessionell im September 2021 (vgl. Online-Zusatzmaterial 1 *Zeit- und Ablaufplan*) statt.

Zum Workshop wurden die Berufsgruppen der Medizin, Physiotherapie und Psychologie/Psychotherapie sowie die Dokumentationsassistenzen – der zum Zeitpunkt Mai–Juli 2021 aktiven 28 PAIN2020-Zentren – zur Teilnahme per Mail eingeladen.

### Sample-Beschreibung und Datenerhebung

**Abb. 2:**
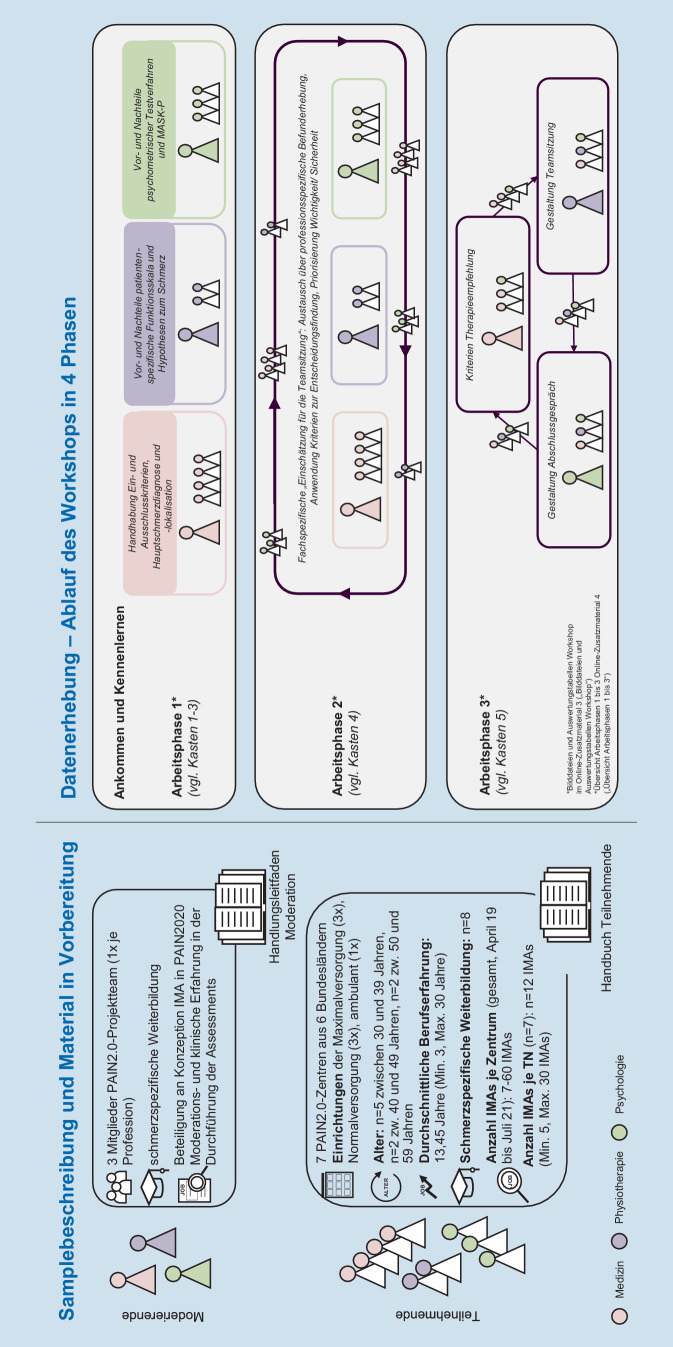
Sample-Beschreibung und Ablauf Workshop/Datenerhebung

Eine Zusammenfassung hinsichtlich der Anzahl der TN und deren soziodemografischer und beruflicher Merkmale sowie die Merkmale der Moderator:innen (Medizin: FP, Physiotherapie: LS, Psychologie: UK) können Abb. 2 entnommen werden.

**Abb. 3:**
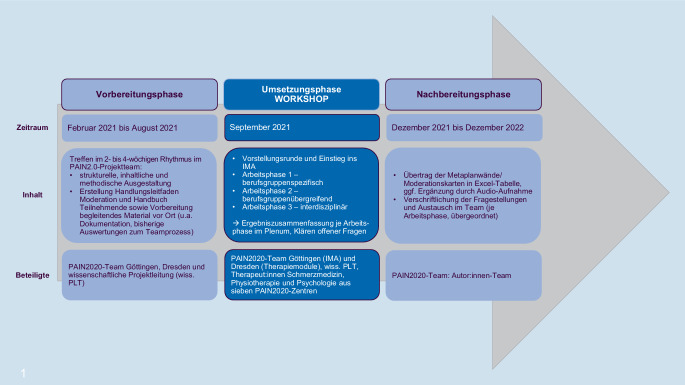
Vorbereitung, Umsetzung und Nachbereitung – Zeitraum, Inhalt und Beteiligte

In Vorbereitung auf den Workshop erfolgte eine strukturelle, inhaltliche und methodische Ausgestaltung (vgl. Abb. 3*„Vorbereitungsphase“*). Mithilfe eines Handbuchs zum Workshop wurden *die TN* im Vorfeld über die organisatorischen Rahmenbedingungen zum Ort und Ablauf des Tages sowie über die übergeordneten Ziel- bzw. Fragestellungen und Inhalte des Workshops zum IMA informiert. Ergänzend wurden die Dokumentationsbögen, das PAIN2020-Handbuch zum IMA sowie bisherige Auswertungen zum Teamprozess im IMA [6, 19, 20] gesammelt zur Verfügung gestellt.

Ein Handlungsleitfaden für die *Moderierenden* beinhaltete ergänzend folgende Informationen: detaillierter zeitlicher Ablauf, geplante Ziele und Fragestellung der jeweiligen Arbeitsphase, eine Kurzbeschreibung der ausgewählten Workshop-Methoden mit Übertrag zum inhaltlichen Bezug sowie die konkrete Planung zur Visualisierung der Ergebnisse (u. a. Metaplanwand, Moderationskarten), Hinweise zum Verhalten und der Rolle als Moderator:in (vgl. Online-Zusatzmaterial 2 *„Rollen und Aufgaben Moderation“*) und die geplante Dokumentation (u. a. Fotodokumentation, schriftliche Notizen) der jeweiligen Arbeitsphasen.

Der Workshop gliederte sich in 4 Phasen (vgl. Abb. 2 und 3*„Umsetzungsphase“*). Die dazugehörigen finalen Bilddateien und Auswertungstabellen für jede Arbeitsphase des Workshops befinden sich im Online-Zusatzmaterial 3 *(„Finale Bilddateien und Auswertungstabellen Workshop“). *Für eine Gesamtübersicht aller Arbeitsphasen verweisen wir auf Online-Zusatzmaterial 4 *(„Übersicht Arbeitsphasen 1 bis 3“)*.

### Datenerhebung und -auswertung

#### Vorbereitung

Während des Workshops wurden alle Aussagen der TN auf Moderationskarten bzw. direkt auf der Moderationswand notiert. Die einzelnen Arbeitsschritte wurden durch die Moderierenden mit (Zwischen‑)Fotos und eigenen Notizen dokumentiert. Ergänzend liefen in jeder Arbeitsphase Audioaufnahmen mit, um im Nachgang unklare Punkte auf den Moderationskarten der Fotodokumentation zu rekonstruieren. Innerhalb der Arbeitsphasen war es den Moderierenden zu jedem Zeitpunkt möglich, flexibel auf die Beiträge der TN zu reagieren und Anpassungen an der Darstellung der Wände vorzunehmen.

In Vorbereitung auf die Auswertung der Ergebnisse wurden die Fotodokumentation (Inhalte der Wände mit den Aussagen der TN) sowie Notizen und Bemerkungen der Moderierenden in eine Excel-Tabelle überführt. Dabei wurde die Gliederung des Workshops in den Arbeitsphasen beibehalten.

#### Vorgehen der Datenauswertung

Zur Auswertung wurden die Ergebnisse je Moderator:in (FP, LS, UK) und je Arbeitsphase zunächst alleinig verschriftlicht, sodass die Inhalte aus dem Workshop nach den Fragestellungen und den inhaltlichen Schwerpunkten der jeweiligen Diskussionen zusammengefasst und mit Beispielen der Aussagen der TN (via Moderationskarten/Metaplanwand) versehen wurden. Zur besseren Verständlichkeit und Nachvollziehbarkeit wurden für die Verschriftlichung der Ergebnisse ergänzend drei Zeichen verwendet, die Hinweise auf die Herkunft der Aussage der TN zulassen (vgl. Infobox 1).

##### Infobox 1

Die Darstellung von qualitativen Ergebnissen unterscheidet sich von der Darstellung der quantitativen Ergebnisse, bei denen es meist um eine statistische Auswertung geht. Für die Präsentation der inhaltlichen Erkenntnisse (qualitative Ergebnisse) werden häufig Einzelbegriffe, paraphrasierte Ausschnitte oder kurze bzw. längere Zitate (direkte Aussagen von Befragten) in den Text aufgenommen. Ergänzend werden aus den Ergebnissen auch Codes bzw. übergeordnete Kategorien gebildet. Dies ermöglicht, Ergebnisse mit Material zu begründen sowie systematisch und nachvollziehbar darzustellen, welche Ergebnisse von den TN selbst bzw. was durch die Auswertungsleistung der Autor*innen entstanden ist. In unserem Vorgehen greifen wir allerdings nicht auf individuelle Aussagen zurück. Wir verwenden Aussagen, die im Rahmen von Gruppendiskussionen entstanden sind. Wir haben uns daher entschieden, das direkte Zitat nicht anzuwenden, sondern die konkreten Äußerungen aus den Gruppen (dokumentiert auf Moderationskarten) mit schrägen Strichen als Rohergebnisse zu kennzeichnen.

**Für die Datenauswertung verwendete Zeichen**

/…/ Wiedergabe der Inhalte der Moderationskarten

[…] Ergänzungen der Autor:innen, die zum Verständnis der Moderationskarten beitragen

Die Ergebnisse für die jeweiligen Arbeitsphasen wurden dann gemeinsam im Moderationsteam (FP, LS, UK) ausgewertet. Dazu wurden die Ergebnisse einer jeden Arbeitsphase untereinander vorgestellt, diskutiert und in der Gegenüberstellung zu den Ergebnissen der anderen Gruppen abgewogen. Es wurde eine konsentierte Gesamtaussage im Hinblick auf die Fragestellung formuliert.

Bei der Auswertung der Arbeitsphase 1 stand insbesondere die Frage im Fokus, welche Anpassungen bzw. Ergänzungen der genutzten Instrumente für eine spätere Umsetzung in der Regelversorgung empfohlen werden.

Für die Arbeitsphase 2 wurden jeweils ergänzend die einzelnen, zur Entscheidungsfindung genutzten (impliziten) Kriterien der drei Berufsgruppen zu den Einschätzungen *„Schweregrad Störungsbild“, „Bedeutung für die Schmerzproblematik“* und *„IMST-Ansatz vs. spezifische Behandlung in Fachbereich“* (vgl. Tab. 1) gesammelt und hinsichtlich Gemeinsamkeiten und Unterschieden zusammengeführt und dargestellt.

Bei der Arbeitsphase 3 wurden zunächst Teamsitzung und Abschlussgespräch voneinander getrennt betrachtet und in einem folgenden Schritt ebenfalls hinsichtlich Gemeinsamkeiten und Unterschieden in Durchführung, Hürden und förderlichen Faktoren zusammengeführt. Ergänzend wurden für die Arbeitsphase 3 die Ergebnisse der Arbeitsphasen 1 und 2 erneut gesichtet, um Zusammenhänge bzw. Einflüsse auf die Umsetzung von Teamsitzung und Abschlussgespräch bzw. Kriterien für Therapieempfehlung zu identifizieren (vgl. Abb. 3*„Nachbereitungsphase“*).

## Ergebnisse

### Arbeitsphase 1: Aufnahmesituation und Befunde

#### Medizin – Ein- und Ausschlusskriterien sowie Hauptschmerzlokalisation und -diagnose

In der ärztlichen Kleingruppe lag der Fokus auf den Erfahrungen der schmerztherapeutischen Einrichtungen im Umgang mit den Ein- und Ausschlusskriterien sowie dem Vorgehen zur Festlegung einer Hauptschmerzlokalisation und -diagnose (vgl. Infobox 2).

##### Infobox 2 Methode, Ziel- und Fragestellung Arbeitsphase 1 (berufsgruppenspezifisch): Medizin

**Zielstellung:** Handhabung der Ein- und Ausschlusskriterien sowie der Hauptschmerzlokalisation und -diagnose

**Methode:** Einzel‑/Gruppenarbeit, Kartenfrage

**Fragestellungen:**

*Ein- und Ausschlusskriterien (B5):*

- Ist die Formulierung des Kriteriums 2 („in kürzeren Phasen innerhalb der letzten beiden Jahre mehrfach auftretend“) zur Beurteilung der Schmerzdauer klinisch geeignet?
- Gab es allgemeine Auffälligkeiten/Schwierigkeiten in der Beurteilung im Studienverlauf?
- War B4 (Screeningfragebogen für die Patient:innen) hilfreich bei der Beurteilung von vorliegenden Chronifizierungsrisiken?

*Hauptschmerzlokalisation und -diagnose* ^2^ *(C5):*

- Wie wurde die Hauptschmerzlokalisation festgelegt?
- Welche Angaben/Informationen wurden dafür genutzt?
- Welche Problemkonstellationen bei der Codierung gab es?
- Wie wurde die Hauptschmerzdiagnose festgelegt (insbesondere, wenn keine F45.41- oder primär psychiatrische Diagnose vorlag) – im Rahmen des Teamprozesses oder durch ärztliche Einschätzung?

*Dokumente* „*B4 Fragebogen für Patienten mit länger anhaltenden Schmerzen“, „B5 Aufnahmebogen Zentrum“ und „C5 Ergänzende Angaben KEDOQ-Schmerz“ im Online-Zusatzmaterial 6 „Dokumentationsunterlagen PAIN2020“*

^2^ *Dieser Teil konnte aufgrund der zeitlichen Vorgaben in der ärztlichen Kleingruppe nicht vollständig beantwortet werden.*

##### Ein- und Ausschlusskriterien.

Die entsprechende Dokumentation zu den Ein- und Ausschlusskriterien (vgl. Online-Zusatzmaterial 6 „B5 Aufnahmebogen Zentrum“) wurde als /gutes Screening/ eingeschätzt, dennoch nutzten die TN zusätzlich Informationen aus dem Deutschen Schmerzfragebogen (DSF) (/Alle DSF genutzt/), der auch in den hier repräsentierten Einrichtungen bereits zum Info-Termin für PAIN2020 vorliege. Unsicherheit bestand dahingehend, ob Patient:innen mit einem hohen Chronifizierungsstadium nach Gerbershagen (Mainz Pain Staging System, MPSS, [7]) grundsätzlich auszuschließen seien (/MPSS III als Ausschlusshinweis/), auch wenn die Ein- und Ausschlusskriterien nicht verletzt wären. Diese Herausforderung in der Einschätzung wurde auch in der Einordnung der Ein- und Ausschlusskriterien mit der Formulierung /Spielraum nötig/ deutlich, sowie in den folgenden Kommentaren:

- Für die /Erhebung Risikofaktoren [einer Chronifizierung]/ und der /Dauer subakut auf chronisch/ sei bei den meist unbekannten Patient:innen neben DSF und dem Fragebogen für Patient:innen (vgl. Online-Zusatzmaterial 6 „*B4 Fragebogen für Patienten mit länger anhaltenden Schmerzen*“) auch ein /subjektiver Eindruck/ berücksichtigt worden.
- Die im Projektverlauf vorgenommene Präzisierung des Einschlusskriteriums „Schmerzen“ (von „in kürzeren Phasen innerhalb der letzten beiden Jahre mehrfach auftretend“ zu „seit einer längeren Zeit wiederholt auftretend oder anhaltend“) sei als hilfreich und klarstellend angesehen worden.
- Auch die im Projektverlauf vorgenommene Präzisierung des Ausschlusskriteriums „Vorliegen einer manifesten chronischen Schmerzerkrankung“ in eine „bekannte chronische Schmerzerkrankung“ wurde unterstützt (/J[a]/), da Letzteres leichter aus der Anamnese abgleitet werden könne (z. B. stattgehabte IMST etc.).
- Zur Einschätzung der /Beeinträchtigung/ bei unbekannten Patient:innen sei das Ergebnis des /von Korff [Fragebogens aus dem DSF] genutzt/ worden, um die Sicherheit der Einschätzung von /zu viel/ versus /zu wenig/ [Beeinträchtigung] zu stärken.

##### Hauptschmerzlokalisation.

Die Zuordnung wurde als /oft klar/ eingeschätzt.

##### Hauptschmerzdiagnose.

Als entscheidend wurde die /klinische Gewichtung/ gesehen, eine ICD R52 (Schmerz, andernorts nicht klassifiziert) wurde nur bei multilokulären Schmerzen als Hauptschmerzdiagnose als geeignet angesehen, die ICD F45.41 (chronische Schmerzstörung mit somatischen und psychischen Faktoren) entsprechend auch als unscharf, da wenig spezifisch in Bezug auf die Lokalisation. Es wurde kritisch angemerkt, dass die Festlegung der Reihenfolge der Diagnosen nicht sicher im Team erfolgt, da sie als Teil der ärztlichen Dokumentation angelegt sei. Daraus lässt sich der Wunsch ableiten, die Diagnosen gemeinsam im Team festzulegen.

#### Physiotherapeutische Einschätzung – Nutzen der PSFS, Einschätzung Hypothesen zum Schmerz und sonstiger Assessmentverfahren

In der physiotherapeutischen Kleingruppe lag der Fokus auf den Erfahrungen der schmerztherapeutischen Einrichtungen in der Handhabung zu Vor- und Nachteilen der Patientenspezifischen Funktionsskala (PSFS, [8]) sowie zur Einschätzung der vorrangigen Hypothesen zum Schmerz ([14]; vgl. Infobox 3).

##### Infobox 3 Methode, Ziel- und Fragestellung Arbeitsphase 1 (berufsgruppenspezifisch): Physiotherapie

**Zielstellung:** Nutzen (Vor- und Nachteile) der PSFS und Hypothesen zum Schmerz im Hinblick auf den physiotherapeutischen Entscheidungs- bzw. Teamprozess

**Methode:** Einzel‑/Gruppenarbeit, Vierfeldertafel (Vor‑/Nachteile vs. Erhebung ja/nein)

**Fragestellungen:**

- Wie praktikabel (Vor- und Nachteile) sind die im IMA eingesetzten Verfahren (1) Patientenspezifische Funktionsskala und (2) Hypothesen zum Schmerz und (3) sonstige Verfahren, die von den Therapierenden angewendet wurden?
- Welchen Nutzen haben die zuvor genannten Verfahren hinsichtlich des physiotherapeutischen Entscheidungsprozesses?

*Dokumente „C7 IMA_Patientenspezifische Funktionsskala“ und „C8 IMA physiotherapeutische Dokumentation“ im Online-Zusatzmaterial 6 „Dokumentationsunterlagen PAIN2020“*

##### PSFS.

Für den Einsatz der PSFS (vgl. Tab. 1) sprächen aus Sicht der TN zahlreiche *Vorteile*. Die PSFS sei /schnell durchführbar/, /praktikabel/, bedarf /wenig Einarbeitung/ und helfe den /Therapierenden einen Eindruck von den [Aktivitäts-]Einschränkungen der Patient:innen zu bekommen/. Der /Fokus [liege] auf der Funktion [und gehe] weg vom Schmerz/. Ergänzend sei die PSFS /hilfreich für die Kommunikation mit den Patient:innen/, für die /[Formulierung der] Zielstellungen/, für /[den Einsatz bei] Verlaufsmessungen/ und könne im Rahmen der /Edukation/ mit eingesetzt werden.

Als ein *Nachteil* wird benannt, dass Patient:innen Schwierigkeiten hätten, ihre Aktivitäten auf der numerischen Skala einzusortieren, da diese entgegengesetzt zu anderen bekannten Skalen (z. B. übliche Schmerzskala) skaliert sei (/Skala ungleich Schmerzskala, (10 = volle Funktion)/).

Ein weiterer *Nachteil* sei, dass es sich lediglich um eine /Momentaufnahme/ handele.

##### Vorrangige Hypothesen zum Schmerz.

*Vorteile *für den Einsatz der Einschätzung zu den vorrangigen Hypothesen zum Schmerz (vgl. Tab. 1) seien, dass es sich um ein /neues Denkmodell/ handele, was wiederum den /Blickwinkel [auf die Schmerzproblematik] erweitere (nicht nur Input-Ebene)/, dabei helfe, die /Befunde besser einzuordnen/, und /hilfreich für die [Auswahl der weiteren] Behandlungsmethoden bzw. Therapiemaßnahmen/ sein könne. Ergänzend wird benannt, dass die Einschätzung eine erste und auch bessere Vorbereitung auf die Teamsitzung biete, selbst dann, wenn die Teamsitzung die Einschätzung ergänze bzw. teilweise auch verändere (/erste Einschätzung für Teamsitzung/).

*Nachteile* seien dagegen vereinzelt die /Unwissenheit bzw. Unklarheit zur Definition der einzelnen Begrifflichkeiten/ und deren entsprechende Einschätzung/Beurteilung (i. S. v. /welche Anzeichen können für die Einschätzung hilfreich sein/). Eine intensive /Einarbeitung [in das Modell sei daher] notwendig/.

#### Psychologie/Psychotherapie – Nutzen von Testpsychometrie und Klassifikation zur Diagnostik im Rahmen des IMA

Im Rahmen der psychologischen/psychotherapeutischen Anamnese für die Befunderhebung im IMA wurden verschiedene vorbereitende testpsychometrische Verfahren (TPV: PHQ-15, SKID-Screening, DASS, PCS, VR-12 u. a.) eingesetzt (vgl. Tab. 1). Darüber hinaus wurde die Gesprächsführung am Ende anhand der MASK‑P [12] als (bisher einzig verfügbares) psychologisches Klassifikationssystem für schmerzrelevante psychische Faktoren eingeschätzt. Die Aufgabe der Arbeitsphase bestand darin, Vor- und Nachteile zum Einsatz standardisierter Verfahren im Rahmen der psychologischen/psychotherapeutischen Anamnese im IMA herauszuarbeiten (vgl. Infobox 4).

##### Infobox 4 Methode, Ziel- und Fragestellung Arbeitsphase 1 (berufsgruppenspezifisch): Psychologie

**Zielstellung:** Nutzen (Vor- und Nachteile) der psychometrischen Testverfahren (SKID-Screening, PHQ-15, DSF inkl. PCS) im Rahmen der psychologischen/psychotherapeutischen Anamnese und Befunderhebung und der MASK‑P als psychologisches Klassifikationssystem für schmerzrelevante psychische Faktoren am Ende der Gesprächsführung im Hinblick auf den psychologischen Entscheidungs- bzw. Teamprozess

**Methode:** Einzel‑/Gruppenarbeit, Vierfeldertafel (Vor‑/Nachteile vs. standardisierte Erhebung ja/nein)

**Fragestellungen:**

- Wie praktikabel (Vor- und Nachteile) sind die verwendeten psychometrischen Testverfahren, insbesondere SKID und MASK-P?
- Welchen Nutzen haben die psychometrischen Testverfahren und die MASK‑P für den Entscheidungsprozess?

*Dokumente „C9 IMA Ergänzender Fragebogen“ und „C11 IMA psychologische Dokumentation inkl. MASK-P“ im Online-Zusatzmaterial 6 „Dokumentationsunterlagen PAIN2020“*

*Vorteile* werden primär in Bezug auf die standardisierte Erhebung im Rahmen der Anamnese berichtet. Die Standardisierung und Strukturierung (/Orientierungshilfe/, /Struktur setzend/) unterstütze eine (möglichst) vollständige Erhebung in kurzer Zeit. Das helfe, die komplexen Faktoren und Strukturen, die an der Erkrankung beteiligt seien, im Blick zu behalten (/„vollständige“ Erhebung S[chmer]z-verarbeitung/, /strukturierte Schmerzanamnese/, /Vielschichtigkeit/) sowie Problembereiche nicht zu übersehen (/Aufmerksamkeit auf wichtige Aspekte [richtend]/, /guter Überblick über Einflussfaktoren/, /Gedächtnisstütze/, /Unterstützung psych[ischer] Eindruck, z. B. Depressivität, Katastrophisierung/). Berufseinsteiger:innen erführen eine Orientierung über relevante Informationen für die spätere Teamsitzung, Diagnostik und Therapieempfehlung (/hilfreich auch bei Berufseinstieg/). Das Vorgehen vermittle somit Sicherheit und wirke zusätzlich unterstützend für Priorisierung, Therapieplanung und -empfehlung (/Wie „tickt“ der Patient/, /Tool zur Therapieplanung/Empfehlung/, /Gewichtung zur Reflexion/).

Durch die Verwendung einheitlicher Begrifflichkeiten entstehe eine Erleichterung in der Formulierung von Befunden (/Hilfreiche Begrifflichkeiten/), die wiederum die Kommunikation mit Teammitgliedern und Nachbehandelnden positiv beeinflusse. Durch die Verwendung der MASK‑P (Multiaxiale Schmerzklassifikation) werde auch die Berücksichtigung nonverbalen Verhaltens erleichtert (/nonverbale Kommunikation im Fokus/). Strukturierende standardisierte Erhebungsinstrumente sollten vor Beginn des Gesprächs vorliegen, sodass die Fachkolleg:innen in der Anamnese Fragen zur Klärung ggf. inkonsistenter Informationen bzw. bei Unvollständigkeit stellen und andere überspringen können. Damit sei eine Zeitersparnis verbunden.

Aus Sicht der TN ergänzten sich vorbereitende standardisierte Erhebung und Anwendung der MASK‑P. Dabei verwendeten alle TN die im Rahmen des IMA vorgegebenen Instrumente konstant und erlebten sie als praktikabel. Einzig der PHQ (Patient Health Questionnaire) wurde als unnötig erlebt. Die TN schätzten die integrative Sicht durch die für PAIN2020 bereitgestellte standardisierte Erhebung und den klinischen Eindruck im Sinne einer gegenseitigen Validierung, die bei nichtstandardisierter Erhebung verloren ginge.

*Nachteile:* Standardisierte Erhebung wird als /zeitintensiv/ und /zeitkritisch/ wahrgenommen. Die Fülle an Informationen aus den vorbereitenden Erhebungsinstrumenten müsse vorher gesichtet und interpretiert werden. Insbesondere die Bearbeitung der MASK‑P erfordere Zeit im Anschluss an die Anamnese. Der Aufwand beim Bearbeiten steige mit der Stärke der Chronifizierung der Erkrankung an. Das erzeuge gelegentlich auch ein /Überforderung[sgefühl]/ bei den Behandelnden, insbesondere dann, wenn Anspruch auf Vollständigkeit bestehe. Gewünscht wurden in diesem Zusammenhang digitale Lösungen (/[zu Doku ergänzend], hilfreich wäre eine Eingabe-Maske mit Textbausteinen/).

Die TN drückten Sorge aus, dass durch die intensive und detaillierte Vorbereitung eine Voreingenommenheit den Patient:innen gegenüber entstehe (im Sinne eines konfirmatorischen Bias; die /innere Offenheit [sinke]/) – mit eingeschränkter Möglichkeit für Beziehungsaufbau und freie Gesprächsgestaltung.

Die Validität von testpsychometrischen Instrumenten wurde intensiv diskutiert (/sonstige Testpsychometrie „überrascht“ oft (meist stärker als berichtet)/). Es komme vor, dass der nach Sichtung der Ergebnisse der Instrumente entstandene Eindruck zu Patient:innen im klinischen Kontext nicht bestätigt werde. Manche Testbefunde legten einen deutlich höheren Leidensdruck nahe, als im klinischen Eindruck vermittelt werde, oder umgekehrt.

Zu fest umrissene Begriffe mit einer geringen Differenzierungsmöglichkeit (MASK-P) bergen die Gefahr für eine /Ungenauigkeit/ oder /Pseudogenauigkeit/. Damit würde eine Über- oder Unterbewertung der psychischen Faktoren am Krankheitsgeschehen einhergehen, die wiederum Einfluss auf die Diagnostik und die Therapieempfehlung haben könne (/„Formulierungen“[der MASK-P] entweder – oder, statt was denkt Patient/). Die Ausformulierungen der MASK‑P wurden teilweise als wenig aussagekräftig beschrieben bzw. war die Art der Einschätzung aus Sicht der TN nicht eindeutig formuliert (/Zwischentöne [in MASK-P] fehlen; eher Kontinuum oder Dimension?/).

### Arbeitsphase 2 – berufsgruppenspezifische Entscheidungskriterien zur Vorbereitung der Teamsitzung

Im Ergebnis der Arbeitsphase 2 liegen jeweils zu den vier Kriterien *„Schweregrad Störungsbild“, „Bedeutung für die Schmerzproblematik“ und „IMST-Bedarf“ vs. „spezifischer Behandlungsbedarf“* berufsgruppenspezifische Aspekte zur Einschätzung sowie eine entsprechende Priorisierung der Wichtigkeit und Sicherheit der benannten Aspekte hinsichtlich der Handhabbarkeit und Unterstützung in Vorbereitung auf die Teamsitzung vor (vgl. Infobox 5).

#### Infobox 5 Methode, Ziel- und Fragestellung Arbeitsphase 2 (berufsgruppenspezifisch/-übergreifend): fachspezifische Einschätzung in Vorbereitung auf Teamsitzung

**Zielstellung:** fachspezifische „Einschätzung für die Teamsitzung“ (vgl. Tab. 1): Austausch über professionsspezifische Befunderhebung und Anwendung der Kriterien zur Entscheidungsfindung in Vorbereitung auf die Teamsitzung, inkl. Priorisierung Wichtigkeit/Sicherheit

**Methode:** Einzel‑/Gruppenarbeit, Markt der Ideen

**Fragestellungen:**

- Welche Kriterien ziehen die Berufsgruppen zur Beurteilung der jeweiligen Einschätzungen heran?
- Welcher Zusammenhang besteht zwischen den vier Einschätzungen?
- Wie beschreiben die Berufsgruppen jeweils die vier übergeordneten Einschätzungen?
- Wie praktikabel ist die Einschätzung in Vorbereitung auf die Teamsitzung?

*Dokumente „C4 IMA ärztliche Dokumentation“, „C8 IMA physiotherapeutische Dokumentation“ und „C11 IMA psychologische Dokumentation inkl. MASK-P“ im Online-Zusatzmaterial 6 „Dokumentationsunterlagen PAIN2020“*

#### Medizinische Aspekte in der Entscheidung

Die Einschätzung des Kriteriums *„Schweregrad Störungsbild“* aus *medizinischer Sicht* basierte auf den Ergebnissen der ärztlichen /Anamnese/, körperlichen Untersuchung (/Untersuchungsbefund/) und der Analyse der Vorbefunde (u. a. Bildgebung, psychiatrische Zusatzerkrankungen).

Bei der Einschätzung des Kriteriums *„Bedeutung für die Schmerzproblematik“* seien neben somatischen Aspekten (u. a. die Einschätzung von Diskrepanzen/Konsistenzen zwischen den somatischen und subjektiven Befunden, Medikamenteneinnahme) weitere psychosoziale oder funktionelle Faktoren (u. a. Bewegungsmuster/-ängste oder Vermeidungsverhalten) sowie patientenbezogene Aspekte (u. a. Störungsmodell, Beeinträchtigung oder Leidensdruck) einbezogen worden.

Sowohl für die Einschätzung des *„Schweregrads Störungsbild“* als auch für die *„Bedeutung für die Schmerzproblematik“* seien die Ergebnisse des MPSS (vgl. Tab. 1; [7]) und von Korff (Bestandteil DSF, [17]) Hilfsmittel von zentraler Bedeutung.

Die differenzierende medizinische Einschätzung zum Kriterium *„IMST/spezifischer Behandlungsbedarf“* berücksichtige vor allem die Wahrnehmung psychosozialer Aspekte, maladaptiver Krankheitsfaktoren, den Bedarf an Schulung und Edukation, ausgeprägte Diskrepanzen zwischen Schwergrad des somatischen Störungsbilds und seiner Bedeutung, allesamt als Hinweise für eine /multimodale Problematik/. Unabhängig davon werde aufseiten der Patient:innen die Notwendigkeit einer /Aufgeschlossenheit für einen interdisziplinären multimodalen Ansatz (IMST)/ gesehen.

Mit Ausnahme der ergänzenden Testverfahren (MPSS, v. Korff) wurden alle zuvor genannten Aspekte zur Einschätzung der jeweiligen Kriterien in ihrer *Wichtigkeit* für die ärztliche Einschätzung als sehr wichtig bewertet, auch wenn sie sich nicht alleinig auf den somatischen Bereich beschränken ließen.

Bei der *Sicherheit* hingegen wurden vor allem professionsspezifische „somatische“ Kriterien als sicherer in der Handhabung eingeschätzt als solche, die Zusammenhänge mit psychologischen und/oder funktionellen/physiotherapeutischen Faktoren aufzeigten. Insbesondere für die Sicherheit bei der konkreten Formulierung eines psychosozialen und funktionellen Störungsbilds, jenseits von „etwas stimmt nicht“, wurde eine zusätzliche Einschätzung der anderen Berufsgruppen mit gemeinsamem Austausch als notwendig gesehen.

#### Physiotherapeutische Aspekte in der Entscheidung

Zur Einschätzung des Kriteriums *„Schweregrad Störungsbild“* aus physiotherapeutischer Perspektive wurden die Ergebnisse der physiotherapeutischen Anamnese und Untersuchungsbefunde (/PT-Befund aktiv/passiv/resistiv/) und die /Konsistenz der Einzelbefunde/ ausgewählt, um zu beurteilen, ob eine funktionelle Problematik vorliegt. Ergänzend spiele das Ergebnis der /PSFS/ – auch für die nachfolgende „Bedeutung für die Schmerzproblematik“ – hinsichtlich der genannten Alltagsaktivitäten und deren subjektiver Einschätzung durch die Patient:innen eine Rolle.

Die rein funktionelle/strukturelle Sichtweise müsse bei der Einschätzung des Kriteriums *„Bedeutung für die Schmerzproblematik“* um die Beeinträchtigungen der Patient:innen in Abhängigkeit vom somatischen bzw. funktionellen Befund (u. a. /Arbeitsfähigkeit/, /Lebensqualität/) erweitert werden. Ergänzend seien die Bewertung der Schmerzen und die Frage des Umgangs mit den Schmerzen (u. a. /Haltung zum Gesundheitsverhalten/, /Vorhandensein funktioneller Ressourcen/) sowie weitere psychosoziale Aspekte berücksichtigt worden.

Insbesondere zwischen *„Bedeutung für die Schmerzproblematik“* und *„IMST-Bedarf/spezifischer Behandlungsbedarf“* berichteten die TN fließende Übergänge durch Überschneidung in den Kriterien. Hierzu zählten u. a. der Stellenwert des Schmerzes für den:die Patienten:in, der Leidensdruck, das Vorhandensein eines biopsychosozialen Modells, das Benennen von schmerzauslösenden Faktoren oder die Notwendigkeit einer interdisziplinären Behandlung.

Weitere Kriterien, die zur Beurteilung *„IMST-Bedarf/spezifischer Therapiebedarf“ *herangezogen worden seien, beinhalteten /spezifische Vorbehandlungen in der Vorgeschichte/, die /Dauer und Häufigkeit der Symptomatik/, das /Stadium im Chronifizierungsprozess/, die /Multilokularität/ der Schmerzen oder auch /Inkonsistenzen im Einzelbefund/.

Eine spezifische physiotherapeutische Behandlung sei immer dann indiziert, wenn sich aus Anamnese und Befunderhebung eine funktionelle Problematik identifizieren ließe (/spez. Behandlung notwendig/). Physiotherapeutisch beurteilbare sowie patientenbezogene Kriterien (/Stellenwert Schmerz für Pat./, /Leidensdruck/ oder /Lebensqualität/) wurden in ihrer *Wichtigkeit* als sehr wichtig eingeschätzt, wovon letztere bei der *Sicherheit* in Ergänzung mit medizinischen bzw. psychosozialen Aspekten gegenüber den rein physiotherapeutischen als weniger sicher in der Handhabung eingeschätzt wurden. Physiotherapiespezifische Aspekte wie der /PT-Befund/ oder die /PSFS/ wurden sowohl in der *Wichtigkeit* als auch in der *Sicherheit* hoch eingeschätzt.

#### Psychologische Aspekte in der Entscheidung

Das Kriterium *„Schweregrad Störungsbild“ *setzt sich aus psychosozialen Belastungsfaktoren, maladaptiven psychologischen Bewältigungsansätzen sowie Hinweisen auf komorbide psychische Erkrankungen zusammen, die im Rahmen der Anamnese erfasst werden. Ergänzend wurde die Dauer der Beschwerden als relevant benannt.

Die Einschätzung in Bezug auf das Kriterium *„Bedeutung für die Schmerzproblematik“* erforderte den TN zufolge zwei erweiternde Perspektiven: a) die anderen Professionen (dysfunktionale Schmerzbewältigung, Ausprägung der Faktoren), da die Befunde in ihrer Bedeutung für die Schmerzproblematik nur in Zusammenschau durch die Professionen festgelegt werden könnten; b) die Abwägung zwischen Ressourcen und Defiziten in Bezug auf eine bestehende Prognose (aus Zeitgründen konnte keine weitere Vertiefung stattfinden).

Die Kriterien *„IMST-Bedarf“ und „spezifischer Behandlungsbedarf“* würden integrativ hinsichtlich der Frage abgewogen, inwieweit Schmerzen und psychosoziale Beeinträchtigungen in relevantem Ausmaß existierten. Die Gefahr der Chronifizierung spiele in Form der prognostischen Abwägung eine wesentliche Rolle. Auch Hinweise auf anstehende therapeutische Erfordernisse würden in die Entscheidung einbezogen, bspw. inwieweit die Patient:innen bereits über ein biopsychosoziales Schmerzmodell verfügten. Abwägungen beträfen u. a. die Passung der Inhalte einer IMST zur betreffenden Person sowie das Vorhandensein von Ressourcen für den Transfer von Therapieerfahrungen in den Alltag.

Spezifischer Therapiebedarf würde vor allem dann erwogen, wenn die Patient:innen psychotherapeutische Ansätze ablehnten bzw. wenn psychische Erkrankungen im Vordergrund oder einer regelmäßigen Teilnahme an einer IMST-Behandlung entgegenstünden. Die psychologischen/psychotherapeutischen Abwägungsprozesse seien komplex und bewegten sich zwischen *allgemein als beeinträchtigend* erlebten psychosozialen Faktoren (Verhaltensweisen, emotionales Befinden, Kontextfaktoren, psychische Störungen), *schmerzbezogenen *psychosozialen Beeinträchtigungen sowie *allgemeinen und schmerzbezogenen* Ressourcen (sowohl intrapsychisch als auch sozial) in Bezug auf funktionelle oder sonstige somatische Limitationen/Ressourcen. Von größter *Wichtigkeit* für die psychologische/psychotherapeutische Entscheidung, einhergehend mit hoher *Sicherheit* in der Einschätzung, seien für die Beurteilung der subjektive Leidensdruck, Hinweise auf psychische Erkrankungen sowie psychosoziale Beeinträchtigungen infolge psychologischer Faktoren (bspw. Antriebsstörungen, Rollenkonflikte etc.).

#### Zusammenfassung

Die Ergebnisse als Integration (vgl. Online-Zusatzmaterial 5 *„Gemeinsamkeiten und Unterschiede in der Einschätzung der Kriterien“*) der drei Professionen zeigen genuin ärztliche, physiotherapeutische und psychologische Aspekte bei der Einschätzung zum Schweregrad des Störungsbilds, die sich von interdisziplinär zu verortenden Aspekten trennen lassen. Insbesondere Informationen aus Anamnese und Befunderhebung mit jeweiliger berufsgruppenspezifischer Sichtweise und Einschätzung sind handlungsleitend bei der Beurteilung des *„Schweregrads Störungsbild“*.

Beginnende Abwägungsprozesse und der Einfluss ergänzender medizinischer (somatischer), physiotherapeutischer (funktioneller) und/oder psychologischer (psychosozialer) sowie patientenbezogener Aspekte – außerhalb der eigenen Berufsgruppe – lassen sich bei der *Integration zwischen „Schweregrad Störungsbild“ und der „Bedeutung für die Schmerzproblematik“* identifizieren.

Die zusätzliche Ebene der Integration der Abwägung der therapeutischen Optionen (*„IMST vs. spezifischer Behandlungsbedarf“*) schließt sich unmittelbar an die vorherigen Einschätzungen an und zeigt neben der Einschätzung der *„Bedeutung für die Schmerzproblematik“* den Bedarf an interdisziplinärer Reflexion und integrativer Entscheidung im Team. Somit werden insbesondere die Komplexität der Abwägungsprozesse der einzelnen Berufsgruppen *und* ein gradueller Übergang zwischen professionsspezifischer hin zu interdisziplinärer Abwägung deutlich.

Hinsichtlich der *Wichtigkeit* der Kriterien zur Einschätzung „Schweregrad Störungsbild“ und „Bedeutung für die Schmerzproblematik“ lassen sich vor allem berufsgruppenspezifische Faktoren erkennen, wobei jede Berufsgruppe unterschiedliche Relevanzen festlegte (vgl. Tab. 2).

**Tab. 2:** Wichtigkeit und Sicherheit (je Berufsgruppe) in Bezug auf die Kriterien zur „Einschätzung für die Teamsitzung“; Skala 0–10, 10 = höchste Sicherheit

Wichtigkeit				Sicherheit		
Psychologie	Physiotherapie	Medizin		Medizin	Physiotherapie	Psychologie
/Subjektiver Leidensdruck/	/Biopsychosoziales Modell/ /PT-Befund passiv/aktiv/resistiv/ /Inkonsistenzen im Einzelbefund/ /Benennen schmerzauslösender Dinge/	/Anamnese/ /Untersuchungsbefund/ /Vorbefunde/	10	/Anamnese/ /Untersuchungsbefund/ /Vorbefunde/ /Medikamenteneinnahme/ /Inanspruchnahme/ /MPSS/ /v. Korff [nur Hilfsmittel]/	/PT-Befund passiv/aktiv/resistiv/ /(In‑)Konsistenz im Einzelbefund/ /PSFS/ /DSF Beeinträchtigung A/Fr/B/ /DSF AU/	/Anzahl [und] Ausprägung psychol[ogischer] Faktoren/ /Biopsychosoziale Belastungsfaktoren/ /Beeinträchtigung Alltag/Arbeit/Freizeit in Folge psychologischer Faktoren/ /Ausmaß der Einschränkungen/
/Hinweise auf psychische Erkrankung/	/Konsistenz der Einzelbefunde/ /Funktioneller Befund/ /Spez. Vorbehandlungen/	/Psychosoziale Faktoren wahrnehmen [statt wichtig]/	9		/Spez. Behandlung notwendig/ /Benennen schmerzauslösender Dinge?/	/Subjektiver Leidensdruck/ /Hinweise auf psychische Erkrankung/
/Beeinträchtigung Alltag/Arbeit/Freizeit [in Folge psychologischer Faktoren]/	/PSFS/ /Stellenwert Schmerz für Pat./ /Spez. Behandlung notwendig/	/Medikamenteneinnahme/ /Inanspruchnahme/ /Korrelation Befund/Sz [Schmerz]/ /Aus Störungsbild result[ierend]: Beeintr[ächtigung], Leidensdruck/	8	–	spez. Vorbehandlung	/[weniger] Ressourcen, innere, äußere, „wie ist der Zugang dazu“/ /Ausprägung Faktoren [und] Einfluss/ /Ausmaß Introspektions- und Reflexionsfähigkeit – [je mehr,] desto besser/
/Ausmaß der Einschränkungen/	/Leidensdruck Lebensqualität (Hobbys, Freizeitgestaltung, …)/	/Aufgeschlossenheit für Therapie (IMST)/ /Zeigt sich eine „multimodale Problematik?“ (Bereits v[or] d[em] Teamgespräch)/	7	–	–	–
/Biopsychosoziale Belastungsfaktoren/	–	–	6	/Psychosoziale Faktoren wahrnehmen [statt wichtig]/	/Lebensqualität (Hobbys, Freizeitgestaltung, …)/	/Dysfunktionale Schmerzbewältigungsart [war vorher bei Wichtigkeit nicht dabei!]/
/Anzahl [und] Ausprägung psychol[ogischer] Faktoren/	/DSF Beeinträchtigung A/Fr/B/	–	5	/Korrelation Befund/Sz [Schmerz]/ /Aus Störungsbild result[ierend]: Beeintr[ächtigung], Leidensdruck/	/Biopsychosoziales Modell/	/Dauer der Beschwerden, Funktionsniveau [war vorher bei Wichtigkeit nicht dabei!]/
/Ausprägung Faktoren [und] Einfluss/	–	/MPSS/ /v. Korff [nur Hilfsmittel]/	4	–	–	–
–	/Arbeitsfähigkeit/ /Dauer Chronifizierungsprozess/	–	3	–	/Dauer Chronifizierungsprozess/ /Stellenwert Schmerz für Pat./	–
/[weniger] Ressourcen, innere, äußere, „wie ist der Zugang dazu“/	/Hypothesen zum Schmerz: Anteil Verarbeitungsebene kognitiv/ /DSF AU/	–	2	–	/Leidensdruck/ /Hypothesen zum Schmerz: Anteil Verarbeitungsebene kognitiv/	–
/Ausmaß Introspektions- und Reflexionsfähigkeit – [je mehr,] desto besser/	–	–	1	–	/Arbeitsfähigkeit/	/Somatische Faktoren, was spielt [unleserlich] eine Rolle? [war vorher bei Wichtigkeit nicht dabei!]/
–	–	–	0	–	–	–

**Abb. 4:**
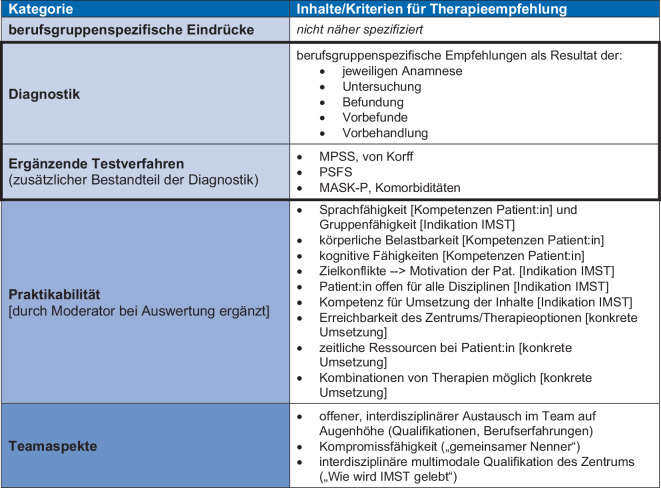
Kriterien für eine Therapieempfehlung zu der Frage: „Welche Inhalte/Kriterien braucht es?“

Eine hohe *Sicherheit* zeigt sich vor allem in Bezug auf die jeweiligen berufsgruppenspezifischen Kriterien. Je mehr Zusammenhänge mit den anderen Berufsgruppen (somatische, funktionelle oder psychosoziale Faktoren) wahrgenommen wurden, desto niedriger die Sicherheit in der Einschätzung und desto mehr wurde durch die TN deutlich gemacht, dass diese Einschätzung ausschließlich zusammen mit den jeweils anderen Berufsgruppen im Teamprozess getroffen werden kann. Auch die Einschätzung und Reflexion patientenbezogener Faktoren wurde in den Teamprozess eingeordnet (vgl. Abb. 4).

### Arbeitsphase 3 – interdisziplinäre Sicht auf die Durchführung von Teamsitzung und Abschlussgespräch

In interdisziplinär zusammengestellten Gruppen wurden in drei rotierenden Durchgängen (World Café) auf Grundlage der Fragestellungen (vgl. Infobox 6) Informationen zu den verwendeten Kriterien einer Therapieempfehlung, zur Teamsitzung (Struktur/Ablauf, Inhalt und Interaktion) sowie zum Abschlussgespräch (Struktur/Ablauf, Übermittlung Therapieempfehlung und Interaktion) auf einer Metaplanwand zusammengetragen und in erste Kategorien unterteilt. Bei den Inhalten zur Teamsitzung und zum Abschlussgespräch wurden jeweils der reale (aktuelles Erleben der TN) und der ideale (Wunschvorstellung der TN) Zustand erhoben. Als Grundlage wurden den TN die Einzeldokumentation der Berufsgruppen sowie die Teamdokumentation aus PAIN2020 zur Verfügung gestellt. Nach jeder Rotation wurden einleitend die Ergebnisse der jeweils vorherigen Gruppe(n) zusammengefasst und anschließend durch die weiteren TN ergänzt, bekräftigt oder hinterfragt (keine Zustimmung zum Ergebnis). In der Gruppe zum Abschlussgespräch beschrieben die ersten beiden Gruppen ihren Ablauf unter real. Die dritte Gruppe wurde gebeten, den idealen Ablauf auszuarbeiten. Alle weiteren Fragestellungen wurden von dieser letzten Gruppe nur ergänzend betrachtet.

#### Infobox 6 Methode, Ziel- und Fragestellung Arbeitsphase 3 (interdisziplinär/interprofessionell): Therapieempfehlung, Teamsitzung und Abschlussgespräch

**Zielstellung: **interprofessioneller Austausch über Inhalte, Abläufe und Verbesserungsvorschläge zur Gestaltung von Teamsitzung und Abschlussgespräch sowie über den Entscheidungsprozess innerhalb der Teamsitzung

**Methode:** World Café, Clustern

**Therapieempfehlung:**

- Welche Kriterien werden für die Therapieempfehlungen hinzugezogen?
- Welche Bedeutung hat in diesem Zusammenhang auch die „Einschätzung für die Teamsitzung“?
- Wie kommt es im Team zu einer Therapieempfehlung?
- Was für Informationen werden von den unterschiedlichen Berufsgruppen benötigt, um eine gemeinsame Empfehlung festzulegen?

**Teamsitzung (real vs. ideal bzw. aktuelles Erleben vs. Wunschvorstellung)**

- Wie gestaltet sich der Ablauf einer Teamsitzung?
- Welche Inhalte werden in der Teamsitzung besprochen?
- (Wie gestaltet sich die Interaktion der Berufsgruppen während einer Teamsitzung? [21])

**Abschlussgespräch (real vs. ideal bzw. aktuelles Erleben vs. Wunschvorstellung)**

- Wie gestaltet sich der Ablauf eines Abschlussgesprächs?
- Wie erfolgt die Übermittlung der Therapieempfehlung?
- (Wie gestaltet sich die Interaktion mit den Patient:innen und den Berufsgruppen? [21])

*Online-Zusatzmaterial 6 Dokumente „C12_IMA interdisziplinäre Teamsitzung und Abschlussgespräch“ und „C13_IMA Algorithmus“*

#### Kriterien für die Therapieempfehlung

Ausgehend von der fachspezifischen Dokumentation in PAIN2020 wurde die *„Einschätzung für die Teamsitzung“* (vgl. Tab. 1, Inhalte aus Arbeitsphase 2) als hilfreich angesehen, um Übereinstimmungen und Abweichungen der einzelnen Teammitglieder zu verdeutlichen und differierende Informationen zu besprechen. Dieses Kriterium und dessen spezifische Inhalte seien kein Teil der Diskussion zu den Therapieempfehlungen und daher in erster Linie /zur Vorbereitung/ sowie zur Verdeutlichung der berufsgruppenspezifischen Eindrücke und Stärkung der Bedeutung des interdisziplinären Austauschs genutzt worden.

Die in der Dokumentation vorgesehene Beurteilung der *Gruppenfähigkeit *(vgl. Online-Zusatzmaterial 6 „C12 IMA Interdisziplinäre Teamsitzung inkl. Abschlussgespräch“) sei in den einzelnen Berufsgruppen als nicht essenziell für die individuelle Einschätzung des Teams im Hinblick auf eine Therapieempfehlung angesehen worden. Dennoch wurde das Kriterium später im Rahmen von Praktikabilitätsüberlegungen als notwendig diskutiert. Diese zunächst widersprüchliche Einschätzung spiegelt am ehesten wider, dass es hier keiner differenzierten Beurteilung aller drei Berufsgruppen bedarf. Auch zeigte sich, dass diese Entscheidung primär der psychologischen/psychotherapeutischen Disziplin obliege, die ja bisher insbesondere die interaktiven Gruppenphasen betreut.

Abb. 4 zeigt die Kriterien bzw. Inhalte, welche die TN in Rotation der interprofessionellen Arbeitsgruppen im Zusammenhang mit der Gestaltung einer *Therapieempfehlung* verwendet haben. Die TN formulierten fünf übergeordnete Kategorien: berufsgruppenspezifische Eindrücke, Diagnostik mit ergänzenden Testverfahren, Praktikabilität und Teamaspekte.

Im Hinblick auf *berufsgruppenspezifische Eindrücke* wurde berichtet, dass Eindrücke auf unterschiedlichen Erfahrungshintergründen beruhen und die Wichtung ähnlicher Informationen beeinflussen. Dieser Prozess finde in der Dokumentation der Einzeleinschätzung für die jeweilige Berufsgruppe den besten Ausdruck – am ehesten im Sinne einer Unterstützung der Reflexion. Davon abgegrenzt wurden die *berufsgruppenspezifischen Empfehlungen* als Resultat disziplinärer Reflexion angesehen. Diese basieren auf der Anamnese, Informationen zur Vorbehandlung, Vorbefunden, Untersuchung und Befundung, ergänzt um *standardisierte Instrumente/Testverfahren*, wie das MPSS, den Schwergrad nach von Korff, die PSFS, die MASK‑P und die somatischen, psychologischen und psychiatrischen Komorbiditäten mit den berufsgruppenbezogenen Schwerpunkten. Alle diese Aspekte wurden unter der Kategorie /*Diagnostik*/ zusammengefasst. Die Abgrenzung der /Eindrücke/ von den Empfehlungen als Teil der /Diagnostik/ verdeutlicht am ehesten, dass jede Berufsgruppe den:die Patienten:in vermutlich breiter wahrnimmt, als das im professionellen Gerüst des IMA zunächst abgebildet ist. Dieser Aspekt der Einschätzung wird weiter unten in der Einschätzung der /Teamaspekte/ deutlich.

Neben den eher berufsgruppenbezogenen /Eindrücke[n]/ und /Diagnostik/ wurde eine Reihe übergeordneter und patientenbezogener Aspekte genannt, die unter dem Oberbegriff */Praktikabilität*/ zusammengefasst wurden. Hier finden sich zunächst ganz grundsätzliche Kompetenzen der Patient:innen [*Kompetenzen Patient:in*], wie /Sprachfähigkeit/, /kognitive Fähigkeiten/, /körperliche Belastbarkeit/. Ebenfalls als notwendig wurden implizitere Voraussetzungen für eine mögliche Wirksamkeit des gemeinsamen interdisziplinären Konzepts [*Indikation IMST*] gesehen: /Patient offen für alle Disziplinen/, /Kompetenz für Umsetzung der Inhalte/, /Gruppenfähigkeit/ vorhanden, geringe /Zielkonflikte/ oder vorhandene /Motivation des Patienten/. Der letzte Aspekt beziehe sich auf die [*konkrete Umsetzung*], wie die /Erreichbarkeit des Zentrums/ oder anderer /Therapieoptionen/, die möglichen /Kombinationen von Therapien/ und /zeitliche Ressourcen beim Patienten/. Der Fokus dieser Dimension der /Praktikabilität/ liegt hier für alle Berufsgruppen zunächst auf der Prüfung einer Indikation für eine IMST, es werden aber im zweiten Schritt auch darüber hinausgehende und sich ableitende Erwägungen berichtet, bezogen sowohl auf den individuellen Patient:innen-„Fall“ als auch auf die lokalen Rahmenbedingungen.

Die Rolle der Interaktion des Teams im Hinblick auf /Eindrücke/, /Diagnostik/ und /Praktikabilität/ bei der Entwicklung konkreter Therapieempfehlungen wird in den /*Teamaspekten*/ deutlich. Hier wurden im Grunde notwendige Voraussetzungen erarbeitet, ohne dass der eigentliche Prozess hätte konkret beschrieben werden können. Ein /offener, interdisziplinärer Austausch im Team auf Augenhöhe/ unter Berücksichtigung von Qualifikationen und Berufserfahrungen der Teammitglieder wurde als Grundlage definiert, ebenso wie /Kompromissfähigkeit/, beschrieben als /kleinster, gemeinsamer Nenner/. Als übergeordneter Rahmen sei dabei die /interdisziplinäre multimodale Qualifikation des Zentrums/, im Sinne von: „Wie wird IMST gelebt“ anzusehen (Weiteres zur Interaktion des Teams findet sich bei [21]).

#### Reale vs. ideale Teamsitzung

##### Struktur und Ablauf Teamsitzung.

Die Aussagen der TN zur Struktur/zum Ablauf lassen sich übergeordnet in drei Kategorien zusammenfassen: *zeitlicher Ablauf, strukturelle Hürden und Gesprächsführung*.

Pro Patient:in würden im Rahmen des IMA für den *zeitlichen Ablau*f aktuell (*real*) /20 bis 30 min/ für die Teamsitzung eingeplant, die (meist) /direkt im Anschluss an die letzte Befunderhebung/ der letzten Berufsgruppe stattfinde und damit schon länger geplant sei als die für die Teamsitzung im Projektprotokoll veranschlagte Zeit (ca. 15 min). Für die TN *ideal* sei ein zeitlicher Rahmen von /mindestens 30 min/, der (1) individuell an die Befunde der Patient:innen angepasst werden könne und (2) abhängig von der gleichen bzw. unterschiedlichen Sichtweise der Berufsgruppen auf die Patient:innen sei (/Differenzen benötigen mehr Zeit/).

Vereinzelt werde die Durchführung der Teamsitzung (*real*) durch das Vorhandensein *struktureller Hürden* in den Einrichtungen erschwert. Insbesondere für die Berufsgruppen, die ihre Befunderhebung zuletzt durchführten, werden Schwierigkeiten berichtet, die spezifischen Befunde für die Teamsitzung zu „integrieren“ bzw. vorzubereiten. Dies betreffe im Besonderen die psychologische/psychotherapeutische Berufsgruppe, da (1) vereinzelt die psychologischen Ergebnisse der Testverfahren des DSF inkl. PCS (Pain Catastrophizing Scale) nicht vorlägen (/psychologische Ergebnisse der Testverfahren teilweise nicht vorliegend, wodurch der […] Abgleich der psychologischen Befunderhebung mit den Werten aus DSF und PCS nicht möglich/) und (2) der Übertrag der Ergebnisse der Befundung in die MASK‑P (/keine Zeit für MASK‑P zuvor/) behindert sei. Als Strategie sei in den Einrichtungen die letzte Befunderhebung auf 45 min verkürzt worden, um in den verbleibenden 15 min die Dokumentation in Vorbereitung auf die Teamsitzung umzusetzen (/45 min Befund + 15 min Doku am Dokument/).

Weiter wurden /Schwierigkeiten im Termin- und Pünktlichkeitsmanagement/ der beteiligten Berufsgruppen zum Start bzw. Ende einer Teamsitzung berichtet. Für die TN *ideal* wären ausreichend inhaltliche und dokumentarische Vor- und Nachbereitungszeit auf die bzw. nach der Teamsitzung und die pünktliche bzw. dauerhafte Anwesenheit aller Berufsgruppen.

Die *Gesprächsführung* werde *real* aktuell fast ausschließlich vom Arzt/der Ärztin übernommen. Diese/r beginne mit der Vorstellung der somatischen Befunde /als Basis, mit der wir starten/ und habe während des Gesprächs eine moderierende Funktion inne (/Arzt beginnt/moderierende Funktion/). Auch in der *idealen* Teamsitzung bestehe der Wunsch nach einer grundlegenden Gesprächsführung (Einführung in das Gespräch und Moderation) durch den:die Arzt:Ärztin. Die TN betonen, dass die Gesprächsleitung grundsätzlich Gesprächsführungskompetenzen erfordert. Diese beträfen unter anderem die Gesprächskultur (z. B. /Raum für Nachfragen/, /Vertrauen/, /Wertschätzung/) und die /Offenheit für fachübergreifende Interaktion/ (z. B. Umgang mit Anmerkungen oder Meinungen einer anderen Berufsgruppe). Ergänzend wird von den TN angeführt, dass die Gesprächsführung nicht durch Rollenbilder beeinflusst werden solle.

##### Inhalte Teamsitzung.

**Abb. 5:**
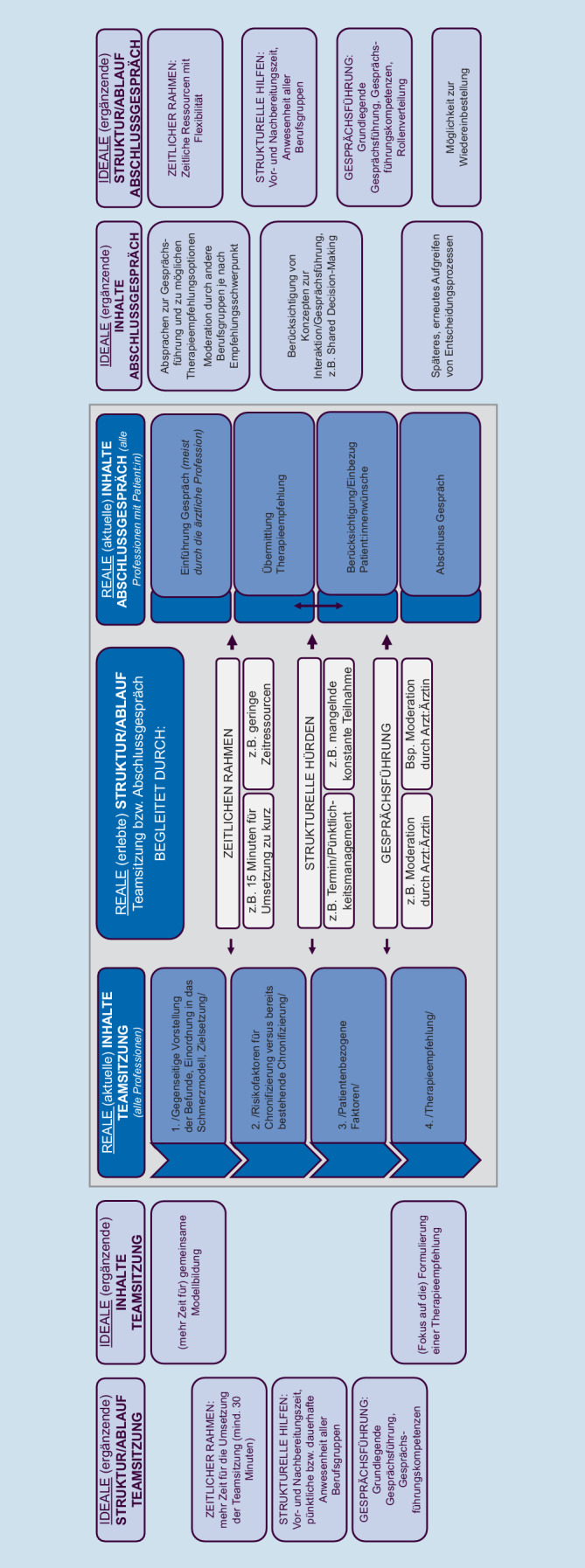
Struktur, Ablauf und Inhalte der Teamsitzung (real vs. ideal) und des Abschlussgesprächs

Die Inhalte der Teamsitzung wurden durch die TN in vier inhaltliche Bausteine eingeteilt (*real*, vgl. Abb. 5), die nacheinander abliefen und je nach Patient:in individuell vertieft würden bzw. optional seien. Sie richteten sich insbesondere nach der im Verlauf der Teamsitzung zur Verfügung stehenden Zeit. Insbesondere der reale Zustand wurde beschrieben, ergänzt um Aspekte für eine ideale Umsetzung. Begleitet werden die Inhalte der Teamsitzung durch die zuvor beschriebenen Strukturen und Abläufe.

1. **/Gegenseitige Vorstellung der Befunde, Einordnung in Schmerzmodell, Zielsetzung/**
  Die Teamsitzung starte meist mit der Vorstellung der /somatische[n] Befunde/ durch die ärztlichen Kolleg:innen, ergänzt um die zusätzlichen Befunde der Physiotherapie und der Psychologie/Psychotherapie. Der Schwerpunkt bei der Vorstellung der Befunde liege auf den jeweiligen /berufsgruppen-spezifischen Auffälligkeiten/. Gelegentlich würden /Vorbefunde/ in die Vorstellung der ärztlichen Kolleg:innen einbezogen, die sich auf Diskrepanzen bei Vorbefunden und/oder vorherigen Diagnosen bezögen. Insbesondere der Frage nach /Komorbiditäten/ (vorwiegend Medizin) sowie der /Lebenssituation/ und /psychosozialen Faktoren/ (vorwiegend Psychologie/Psychotherapie) komme zu diesem Zeitpunkt eine bedeutende Rolle zu.
  Psychologische Testverfahren (v. a. DSF, PCS, SKID – Strukturiertes Klinisches Interview), ärztliche Testverfahren (u. a. MPSS, von Korff) und physiotherapeutische Verfahren (u. a. PSFS, Hypothesen zum Schmerz) flössen indirekt in die Befundvorstellung ein (vgl. Arbeitsphase 1 und 2), würden aber nicht konkret für jede:n Patienten:in berücksichtigt. Die Beurteilung der vorrangigen Hypothesen zum Schmerz durch die Physiotherapeut:innen würden größtenteils nicht angeführt, da Unsicherheiten hinsichtlich der Einschätzung bestünden, die sich zum Teil erst durch die Teamsitzung ergäben (/keine Hypothesen zum Schmerz → Unsicherheit/). Je nach Patient:in würden zusätzlich Diskrepanzen im /Befund versus Verhalten/ und/oder /Testverfahren versus Anamnese/ besprochen.
  Abschließend würden die Befunde durch die Berufsgruppen hinsichtlich deren subjektiver Wahrnehmung der Patient:innen eingeordnet (/Wie „wirkt“ der:die Patient:in?/), um eine erste Beurteilung vorzunehmen (/Welches Schmerzmodell hat der:die Patient:in?/). Jede Berufsgruppe stelle dazu aus der persönlichen Sicht das Modell für den:die Patienten:in vor. Dabei erfolge eine erste Integration aller Sichtweisen. Eine vollständige Integration innerhalb der Teamsitzung sei nach Aussagen der TN aufgrund der zeitlichen Limitation nur selten möglich, da jede Berufsgruppe zum Zeitpunkt der Teamsitzung ein eigenes Modell über den:die Patienten:in habe, das sich meist erst vollständig beim Schreiben des Befundberichts entwickele. Die TN wünschen sich *ideal*erweise – auch im Hinblick auf die Formulierung der Therapieempfehlung – mehr Zeit für eine gemeinsame Modellbildung.
  Zum Abschluss des ersten Bausteins würden aus jeder Berufsgruppe die Ziele der Patient:innen vorgestellt und die wichtigsten Teamziele zusammengefasst. Diese setzten sich meist aus Einzelzielen der Berufsgruppen zusammen.
2. **/Risikofaktoren für Chronifizierung versus bereits bestehende Chronifizierung/**
  Nach Vorstellung der Befunde, einer ersten Entwicklung eines Schmerzmodells und Formulierung der Teamziele erfolge eine /Integration der Befunde/ zu der Frage /Risiko einer Chronifizierung vs. bereits chronifiziert?/. Als Beurteilungskriterien wurden berichtet: /Umgang mit Schmerzbewältigung/, /Verhalten/ (u. a. persönlich, aktivitätsbezogen), Vorhandensein eines ausgeprägten /Schonverhalten[s]/ oder das Vorhandensein von /Durchhaltestrategien/.
3. **/Patienten-bezogene Faktoren/**
  Im nächsten Schritt würden patientenbezogene Faktoren einbezogen. Hierzu zählten u. a. der /Leidensdruck/ der Patient:innen, die /Motivation und Compliance/ der Patient:innen (/Was traut man dem:der Patienten:in zu, was kann er:sie umsetzen?/) sowie die Frage nach Unterstützungsbedarf durch die jeweiligen Berufsgruppen (/Wie viel Unterstützung braucht er:sie in den verschiedenen Bereichen?/).
4. **/Therapieempfehlung/**
  Zum Abschluss – und nach Aussage der TN eher beiläufig – erfolge die Formulierung einer Therapieempfehlung, die sich am /Schwerpunkt/Bedarf der Patient:innen/ (i. S. der zuvor angeführten Punkte, z. B. Befunde), den /individuellen Möglichkeiten der Patient:innen/ (u. a. Anreise, Wartezeit für Empfehlung) und den Möglichkeiten der Einrichtung bzw. der Indikationsstellung orientiere mit dem /Ziel der „besten“ Behandlungsempfehlung/. Einzelne Therapieempfehlungen würden gegenübergestellt und durch das Team abgewogen. Gelegentlich würden vorherige Therapien miteinbezogen.
  Die Formulierung einer Therapieempfehlung gehe laut Aussage der TN mit Unsicherheit und Uneinigkeit innerhalb der Teamsitzung sowie bei der Vermittlung im anschließenden Abschlussgespräch einher. Insbesondere in Bezug auf das Erfordernis eines IMST-Ansatzes zeige sich Unsicherheit (v. a. Physiotherapeut:innen) bzw. Uneinigkeit zwischen den Berufsgruppen.
  Da die Empfehlung bisher in der Teamsitzung keinen direkten Fokus habe, wünschen sich die TN dies als gemeinsames Ziel der Teamsitzung (/die Empfehlung im Fokus/).

#### Reales vs. ideales Abschlussgespräch

##### Struktur, Ablauf und Inhalte – Abschlussgespräch.

In der *Realität* berichteten die Teams eine unterschiedliche Gestaltung im Ablauf des Abschlussgesprächs vor allem in drei Bereichen: die Reihenfolge ähnlicher Inhalte (Erklärung IMST, Profession etc.), die explizite Berücksichtigung von Patient:innenwünschen sowie beim Abschluss des Gesprächs (Fragen/Zusammenfassung vs. Missverständnisse klären, Verabschiedung) (vgl. Abb. 5).

Einig waren sich die Gruppen hinsichtlich der *Hürden* (v. a. mangelnde Konstanz der Teilnahme vonseiten der Professionen, geringe Zeitressourcen). Der Zeitmangel wurde insbesondere dann als hinderlich wahrgenommen, wenn viele Optionen zur Auswahl stünden oder Patient:innen sehr klare Vorstellungen hätten, die jedoch den Empfehlungen aus dem Team entgegenstünden. Als limitierend wurde auch erlebt, dass Entscheidungsprozesse nicht noch einmal aufgegriffen oder ihnen mehr Zeit durch eine Wiedereinbestellung eingeräumt werden konnten.

Für den *idealen* Ablauf ergaben sich vier Bereiche, die mit den Ergebnissen der Interaktion der Berufsgruppen untereinander zusammenhängen. Neben den erforderlichen zeitlichen Ressourcen, die eine gewisse Flexibilität zuließen (je nach Bedarf vonseiten der Patient:innen und/oder Komplexität der Erkrankung und Therapieempfehlungen), bedürfe es jedoch auch einer gewissen Qualifikation in der Gesprächsführung vonseiten der Mitarbeitenden, eines klaren, wenig hierarchischen Rollenverständnisses im Team, Absprachen zur Gesprächsleitung und -verteilung sowie der Berücksichtigung des Konzepts des *Shared Decision-Making*.

##### Übermittlung der Therapieempfehlung – Abschlussgespräch.

Erfahrungen aus der *real*en Umsetzung in Bezug auf die Übermittlung von Therapieempfehlungen lassen sich in drei Kernbereiche gliedern (mit Überschneidungen zur Interaktion im Abschlussgespräch, die ausführlich bei [21] beschrieben werden): a) Voraussetzungen für die Entscheidungsfindung mit Patient:innen, b) Struktur der Informationsvermittlung und c) Rolle (des:der Einzelnen des Teams) bei der Vermittlung der Therapieempfehlungen.

Es wurde empfohlen (*ideal*), für die gemeinsame Entscheidungsfindung mit den Patient:innen zunächst an die Befunde anzuknüpfen. Darüber hinaus wurde als hilfreich erlebt, wenn verschiedene Empfehlungsoptionen im Vorfeld im Team vorbereitet und abgestimmt werden oder Folgetermine vereinbart werden konnten.

Eine Priorisierung und Abwägung notwendiger Informationen wurde im Rahmen der Teamsitzung in Vorbereitung für das Abschlussgespräch als unabdingbar, aber noch nicht regelhaft üblich angesehen.

Für diese komplexen Anforderungen an das Abschlussgespräch (in Ergänzung Teamsitzung) erscheine es den TN als notwendig, Zeit in der Vorbereitung einzuplanen (Vorstrukturierung, Priorisierung der Informationen und Empfehlungen, nächste Schritte; wer sagt im Team was wann zu wem). Es wurden auch schriftliche Empfehlungen direkt für die Patient:innen angedacht, die als Gedankenstütze dienen sollen [21].

## Diskussion

Im Rahmen des hier dargestellten Workshops zu Ablauf und Entscheidungskriterien und -prozessen des interdisziplinären multimodalen Assessments (IMA) wurden Mitarbeitende der drei beteiligten Fachprofessionen befragt, die an der Umsetzung des IMA im Rahmen von PAIN2020 aktiv beteiligt waren. Das inhaltliche Vorgehen basierte einerseits auf den Empfehlungen von Casser et al. [4], geht aber in vielen Aspekten darüber hinaus, da spezifische Neuerungen in der Anpassung an die Zielgruppe und zur spezifischen physiotherapeutischen und psychologischen Diagnostik (PSFS, SKID, PHQ-15) ergänzt wurden.

Die **Ein- und Ausschlusskriterien aus PAIN2020** (aus Arbeitsphase 1 – Medizin) wurden von ärztlicher Seite als tauglich für die intendierte Patientengruppe eingeschätzt, auch wenn im IMA eine nicht immer eindeutige Abgrenzung zu höher chronifizierten Patient:innen möglich war. Dies stellt im Sinne einer ergebnisoffenen, frühzeitigen Therapiesteuerung kein grundsätzliches Problem dar. In solchen Fällen wurden entsprechenden Patient:innen (frühzeitig) intensive Angebote der IMST (stationär/teilstationär) empfohlen.

**Die in PAIN2020 verwendeten etablierten und ergänzenden physiotherapeutischen und psychologischen Verfahren zur Befundung** (aus Arbeitsphase 1 – Erhebung Vor- und Nachteile) wurden vor dem Hintergrund der ressourcenorientierten Informationssammlung komplexer Konstellationen durch die TN als bedeutsam und im Wesentlichen auf vielen Ebenen hilfreich geschildert (u. a. PSFS bei der Physiotherapie). Im Prinzip überwiegen aus Sicht der TN die Vorteile eines standardisierten Einsatzes von Erhebungs- und Testinstrumenten für das IMA als auch der Einzelbefunderhebung (i. S. v. Schwerpunktsetzung und Strukturierung in der inhaltlichen Ausgestaltung).

Die Anwendung vereinfachender Modelle und Vorgehensweisen als Standardvorgehen bei der Befundung und der im Nachgang erstellten professionsspezifischen Befundintegration sollte aber, wie am Bsp. der Physiotherapie diskutiert wurde (Anwendung der Hypothesen zum Schmerz), intensiv geschult werden. Dabei wären Formate wie Tutorien oder Fallbesprechungen hilfreich. Es gab Überlegungen, ob die Einschätzung der vorrangigen Hypothesen zum Schmerz, die im Moment eher vonseiten der Physiotherapie bearbeitet wird, auch ein geeignetes Schema für die anderen Professionen sei und damit als eine Möglichkeit zur Entwicklung einer gemeinsamen Sprache im Team dienen könne.

Für die psychologischen/psychotherapeutischen Aufgaben lässt sich zusammenfassen, dass die in PAIN2020 gewählten Methoden zur Befundung im Wesentlichen hilfreich erlebt wurden. Es erscheint jedoch sinnvoll, für die Rolle des:der Psychologen:in/Psychotherapeuten:in im IMA Schwerpunkte zu definieren bzw. herauszuarbeiten, welche wesentlichen Kerninformationen erhoben und auf welche Aspekte (sozial, intrapsychisch) hier das Augenmerk gelegt werden sollte. Das setzt eine fachbezogene, evidenzbasierte Auseinandersetzung mit der Bedeutung von psychosozialen Risikofaktoren voraus. Aktuell setzen Psycholog:innen/Psychotherapeut:innen die Schwerpunkte in der Fülle und Komplexität der Risikofaktoren letztlich subjektiv, mit der Gefahr der Fehl- oder Überbewertung psychischer Einflussfaktoren. Ergänzende Verfahren wie die PCS, das SKID-Screening oder die Anwendung der MASK‑P wurden begrüßt, die PHQ allerdings als verzichtbar angesehen. Die TN nahmen in der Diskussion wenig divergente Perspektiven ein. Die Balance zwischen Vollständigkeit und Richtigkeit von Informationen zur Beurteilung psychischer Faktoren am Krankheitsgeschehen einerseits und ausreichend Offenheit und Zeit zur Entwicklung einer vertrauensvollen Beziehung andererseits erschien von zentraler Bedeutung.

Mit den in PAIN2020 vorgesehenen **professionsspezifischen Abwägungs- und Entscheidungsprozessen** der Berufsgruppen (vgl. Arbeitsphase 2) lassen sich neben genuin professionsspezifischen Entscheidungskriterien durch die anderen Berufsgruppen jeweils ergänzende somatische, funktionelle und/oder psychosoziale sowie patientenbezogene Aspekte identifizieren. Entsprechend den Rückmeldungen handelt es sich einerseits um einen komplexen Entscheidungsprozess, der nicht linear verstanden werden kann und zur differenzierten Abwägung vorhandener therapeutischer Optionen (IMST-Bedarf vs. spezifischer Therapiebedarf) eines integrativen Teamprozesses bedarf. Zum anderen wird deutlich, dass es neben berufsgruppenspezifischen Kernkriterien (u. a. Anamnese, Befunderhebung) auch berufsgruppenübergreifende Kernkriterien (u. a. Lebensqualität, Stadium Chronifizierungsprozess) gibt, die wiederum Einfluss auf den Ablauf des Teamprozesses haben. In jedem Fall spielt aus Sicht der TN eine an die Bedarfe und Zielstellungen der jeweiligen Zielgruppe angepasste standardisierte Erhebung (auch der jeweiligen professionsspezifischen Befundung) eine große Rolle für die hochqualitative Umsetzung interdisziplinärer Schmerztherapie.

Die **strukturellen Abläufe einer Teamsitzung** in den Einrichtungen werden als prinzipiell klar und etabliert beschrieben. Die Umsetzung selbst wird durch verschiedene Barrieren begleitet, die vor allem in der Begrenztheit von Zeit für Teamsitzungen und Absprachen und in den strukturellen Hürden liegen. Diese Besonderheiten interdisziplinärer Zusammenarbeit wurden offensichtlich bisher auch in PAIN2020 nicht ausreichend berücksichtigt, erscheinen aber von großer Bedeutung für das Gelingen interdisziplinärer Zusammenarbeit.

Die **Inhalte der Teamsitzung** gestalten sich individuell je Patient:in und werden durch die TN auf zwei Ebenen verortet: Ebene der Therapeut:innen (u. a. Schwerpunktsetzung, Auffälligkeiten der Befunde) und Ebene der Patient:innen (u. a. Bedürfnisse der Patient:innen). Es zeigt sich in Übereinstimmung mit den Ergebnissen der Arbeitsphase 1, dass die berufsgruppenspezifischen „Fakten“ aus der Diagnostik mit den jeweiligen Eindrücken und der Einschätzung der patientenindividuellen Faktoren (primär aus der Sicht des IMST-Konzepts) in die Teamsitzung einfließen. In einem „idealerweise“ offenen, konsensorientierten und interdisziplinären Austausch werden dann die Therapieempfehlungen entwickelt. Gleichzeitig wird von den TN benannt, dass die „differenzierte“ Therapieempfehlung eher beiläufig erfolgt und noch kein integraler Bestandteil der Teamsitzung als solches ist. Häufig besteht bei den TN der Eindruck einer verstärkten „Detailorientierung“, da zu viele (nichtrelevante) Aspekte besprochen werden, ohne dass eine Priorisierung der Inhalte stattfindet. Wünschenswert wären hier möglicherweise Modelle zur Priorisierung, die unter Berücksichtigung der multidisziplinären Perspektiven und knapper Zeitressourcen umsetzbar sind und eine gemeinsame Formulierung der Therapieempfehlung und Diagnosen ermöglichen. Eine begleitende Teamdokumentation sollte daher sowohl die Inhalte als auch Prozesse einer Teamsitzung abbilden.

Die TN des Workshops betonen, dass sie das **gemeinsame Abschlussgespräch** aller Disziplinen für ein wichtiges Instrument halten, die biopsychosoziale Sicht, Diagnostik und die daraus resultierenden Empfehlungen in kurzer Zeit den Patient:innen zu vermitteln. Es wurde berichtet, dass die gemeinsame Durchführung zu einer höheren Akzeptanz der Empfehlungen durch die Patient:innen führt, Fragen schnell geklärt und Wechselwirkungen in den Risikofaktoren besser dargestellt werden konnten.

Unsicherheit zeigt sich in der **Durchführung des Abschlussgesprächs**, das bisher mit Teilnahme aller Berufsgruppen am Abschlussgespräch in der Regelversorgung noch nicht etabliert ist. Die Unsicherheit wird bedingt durch die mangelnde Vorbereitung des Abschlussgesprächs im Rahmen der Teamsitzung, die bisher offensichtlich noch kein inhaltlicher Bestandteil der Teamsitzung ist. Insbesondere die Befunde werden zwar im Team abgewogen und priorisiert, die Therapieempfehlung jedoch nur bedingt. Die Rollenverteilung für das Abschlussgespräch wird im Vorfeld nur gelegentlich abgesprochen und entsteht eher spontan und unstrukturiert. Eine Vermittlung der gemeinsam erarbeiteten Diagnosen und Therapieempfehlungen gegenüber den Patient:innen erscheint somit erschwert. Ungeklärt ist auch der Umgang mit besonderen Situationen im Abschlussgespräch (bspw. wenn keine gemeinsamen Entscheidungen mit den Patient:innen getroffen werden konnten oder diese mehr Zeit zum Verstehen oder zur Entscheidung benötigten). Das Abschlussgespräch erfordert demzufolge Erfahrungen und Qualifikationen, die im Team gemeinsam und nicht allein in einer Profession geschult werden sollten.

Auch die **Inhalte des Abschlussgesprächs** werden als weniger klar berichtet. Die Ausgestaltung, die den Teams in PAIN2020 selbst überlassen war, wurde sehr unterschiedlich vorgenommen, die Folge waren erhöhte Unsicherheiten und Unzufriedenheiten. Es wurden Konzepte wie das Shared Decision-Making als Ideen für eine bessere Umsetzung genannt. Hinderlich war auch eine in der Teamsitzung noch nicht bis ins Detail geklärte Diagnostik oder Empfehlung, wodurch die Vermittlung des Schmerzmodells als auch der Empfehlungen nur unzufriedenstellend dem:der Patienten:in gegenüber gelang.

Ein immer wiederkehrendes Thema im Rahmen der Arbeitsphasen waren die **Interaktion und sich daraus ableitende Schritte für die interdisziplinäre Zusammenarbeit **(siehe ausführlicher [21]).

Hinsichtlich der eingangs formulierten Fragestellungen zeigt sich, dass (1) das IMA mit kleineren Anpassungen und konkreten Empfehlungen zur Umsetzung aus klinischer Perspektive umsetzbar ist; (2) Struktur- und Prozessparameter in der Umsetzung weiterhin ausgebaut und berücksichtigt werden müssen und (3) darüber hinaus weitere Aspekte von Bedeutung sind, u. a. Qualifikation, Methoden der Gesprächsführung [21].

Ergänzend ergab sich aus diesen Diskussionen wiederholt die Frage, wie die Patient:innen inhaltlich und kommunikativ in den interdisziplinären Teamprozess „integriert“ werden können. Themen wie Motivation, Verhaltensänderung, „der mündige Patient“, Health Literacy, Gesundheitskompetenz oder Shared Decision-Making wurden als Konzepte von Relevanz durch die TN in den Raum gestellt. Die TN drückten ein ausgeprägtes Bedürfnis nach evidenzbasierten, (teil-)standardisierten inhaltlichen Konzepten für die Umsetzungen von Teamprozess und Abschlussgespräch im Rahmen des IMA aus. Unserem aktuellen Kenntnisstand nach liegen solche Konzepte für die Schmerzmedizin derzeit nicht vor. Gleichzeitig leisten die Ergebnisse des Workshops einen ersten Beitrag – neben bestehenden Struktur- und Prozessparametern – zum Wirkfaktor Teamzusammenarbeit bzw. deren einzelnen Mitgliedern im Rahmen des IMA. Um zukünftig förderliche bzw. hinderliche Wirkfaktoren zu identifizieren, muss die IMST zukünftig selbst Gegenstand der Forschung werden.

### Limitationen

Die Ergebnisse unserer Arbeit geben einen Einblick in die Handhabung und Umsetzbarkeit des IMA bei Patient:innen mit wiederkehrenden Schmerzen und Chronifizierungsrisiko. Sie weisen eine große Nähe zur klinischen Erfahrung im Versorgungsalltag auf. Das gewählte Format bot einerseits eine gute Struktur zur Bearbeitung der gesetzten Fragestellungen, es bot aber auch eine gewisse Flexibilität in der Anwendung, wenn Inhalte von den TN selbst weiterentwickelt werden wollten. Diese Flexibilität führte allerdings zu einer teilweise verminderten Vergleichbarkeit der Ergebnisse zwischen den Berufsgruppen und in der Folge zu einer leicht eingeschränkten Integration der Ergebnisse über die Bearbeitungsschritte (s. z. B. Infobox 2). Dieser Nachteil wurde jedoch aus Sicht der Autor:innen damit aufgehoben, dass wir einen intensiven, neuartigen Einblick in die Umsetzung des IMA und der Teamarbeit aus Sicht von Tätigen erhalten haben. Ein weiterer, aus unserer Sicht limitierender Faktor besteht in der begrenzten TN-Anzahl. Daher sehen wir die Ergebnisse unserer Arbeit in erster Linie als explorativ. Eine Sättigung der Ergebnisse war nicht vorgesehen und wurde ganz sicher auch nicht erreicht.

## Fazit

Hinsichtlich der Fragestellungen kann zusammenfassend sowohl die inhaltliche Ausgestaltung des IMA durch ein standardisiertes Vorgehen (Einschlusskriterien für die Zielpopulation, Vorbereitung, Befundung, Vorbereitung zur Teamsitzung sowie die Entscheidungskriterien für die Ableitung von Empfehlungen) als auch die Umsetzbarkeit generell bestätigt werden. Aus dem Material des Workshops ergaben sich allerdings vielfältige Anregungen, die bei der Übernahme des IMA in die Regelversorgung in Ergänzung zu bereits bestehenden Empfehlungen zu Struktur- und Prozessparametern der Deutschen Schmerzgesellschaft e. V. aufgegriffen werden sollten.

Die für PAIN2020 entwickelten standardisierten Befundungs- und Testverfahren wurden von den TN mehrheitlich als hilfreich und unterstützend erlebt. Ein Ausrollen dieser Befunde für die Anwendung dieses IMA in der Regelversorgung wurde mit einigen Anpassungen für das A‑IMA (www.a-ima.de) aus dem Workshop abgeleitet und umgesetzt.

Trotz ausführlicher Empfehlungen zu Struktur- und Prozessparametern [1, 2, 4, 16, 18] sind die Teams in der inhaltlichen und prozessbezogenen Ausgestaltung der Teamsitzung und des Abschlussgesprächs noch auf ihre eigene Kreativität angewiesen. Modelle oder standardisierte Vorgehensweisen (einschl. Gesprächs- und Reflexionskultur) sollten in Fachgremien in Form von Konzepten erarbeitet und wissenschaftlicher Überprüfung zugänglich gemacht werden. Darüber hinaus sollten in Schulungen zur Schmerzmedizin entsprechende Kompetenzen, einschließlich der Gesprächsführung, adressiert werden.

## Supplementary Information

1. Zeit- und Ablaufplan

2. Rollen und Aufgaben Moderation

3. Bilddateien und Auswertungstabellen Workshop

4. Übersicht Arbeitsphasen 1 bis 3

5. Gemeinsamkeiten und Unterschiede in der Einschätzung der Kriterien

6. Dokumentationsunterlagen PAIN2020

## Footnotes

1 Einzelne Bestandteile des Workshops sind bereits im eingeladenen Schwerpunktheft „Interdisziplinäre multimodale Schmerztherapie“ erschienen (10.1007/s00482-023-00740-7). Daher kann es zu inhaltlichen Überschneidungen in Einleitung und Methodik kommen.
