# Supplementary material for: Feasibility of the interdisciplinary multimodal assessment—The team perspective
Source: Schmerz. 2024 Apr 9;39(6):398–416. [Article in German] doi: 10.1007/s00482-024-00796-z (PMC12627191; doi:10.1007/s00482-024-00796-z)
Supplement: Supplementary file 1 — 1. Zeit- und Ablaufplan [file 482_2024_796_MOESM1_ESM.pdf]

Online-Zusatzmaterial 1 – Zeit- und Ablaufplan Workshop IMA

Zeit	Ablauf und Inhalt
10:00 Uhr	**Ankommen**
10:30 Uhr	**Eröffnung und Begrüßung,** inkl.<br>• Vorstellung Projektteam<br>• Ablauf heutiger Tag<br>• Gruppenaufteilung ärztlich, physiotherapeutisch und psychologisch
10:50 Uhr	**Vorstellung innerhalb der Kleingruppen (berufsgruppenbezogen) und Einstieg in den Tag**<br>• Kurzvorstellung der Teilnehmenden (Name, Einrichtung, Motivation der Teilnahme am Workshop)<br><br>Kurzeinstieg ins IMA:<br>• Wir im IMA …<br>• Das IMA ist …<br>• Unsere Rolle heute …<br>• Wir wünschen uns für heute …
11:15 Uhr	**Arbeitsphase I***(berufsgruppenbezogen)*
12:15 Uhr	**Mittagspause**
13:15 Uhr	**Arbeitsphase II** *(berufsgruppenbezogen/-übergreifend*
15:00 Uhr	**Arbeitsphase III – Teil 1** *(interprofessionell)*<br><br>3 Thementische:<br>• Teamsitzung<br>• Therapieempfehlung<br>• Abschlussgespräch
16:00 Uhr	**Kaffeepause**
16:30 Uhr	**Arbeitsphase III – Weiterführung Teil 1** *(interprofessionell)*
17:00 Uhr	**Arbeitsphase III – Teil 2** *(interprofessionell)*
18:00 Uhr	**Gemeinsamer Abschluss/Ausklang**
18:30 Uhr	**Ende**
