# Supplementary material for: Feasibility of the interdisciplinary multimodal assessment—The team perspective
Source: Schmerz. 2024 Apr 9;39(6):398–416. [Article in German] doi: 10.1007/s00482-024-00796-z (PMC12627191; doi:10.1007/s00482-024-00796-z)
Supplement: Supplementary file 2 — 2. Rollen und Aufgaben Moderation [file 482_2024_796_MOESM2_ESM.pdf]

Online-Zusatzmaterial 2: Rollen und Aufgaben Moderation

Rolle/Aufgaben in Vorbereitung auf die Arbeitsphase
• Vorbereitung des benötigten Materials (u.a. zur Visualisierung, als Hilfsmaterial) und weiterer organisatorischer Maßnahmen<br>• Inhaltliche Vorbereitung mit Fokussierung auf die vorformulierten Fragestellungen<br>• ggf. räumliche Gestaltung
**Rolle/Aufgaben während der Arbeitsphase**
Umsetzung/Organisation:<br>• Einführung in die Durchführung und der Inhalte der Arbeitsphase<br>• Einhaltung des festgelegten Zeitplans und der geplanten Struktur, ggf. flexible Anpassung<br>• Geplante Ziele im Blick behalten und erreichen, Fragestellungen für die Arbeitsphase vorstellen; Klarheit der Aufgabenstellung erfragen<br>• Dokumentation/Visualisierung aller benannten Aspekte bzw. konkreten Ergebnisse (z.B. Karteikarten, Metaplanwand), auch durch die Teilnehmenden möglich<br>• Regeln erklären (z.B. ein Gedanke pro Karte, Schrift, Kurzsatz...)<br><br>Gesprächsführung – Leiten der Diskussion:<br>• Fokussierung der Teilnehmenden auf die Fragestellung<br>• Abweichler und Vielredner „einfangen“ und auf den Punkt bringen<br>• stille TN einbeziehen, dominante Teilnehmende bremsen<br>• mögliche Widerstände konstruktiv bearbeiten<br>• Einladen zum Einkreisen, Markieren oder Hervorheben von wichtigen Aspekten und Durchstreichen von als unwichtig empfundenen Aspekten<br>• Eigene Meinung und Informationen zurückhalten; den Prozess nicht stören, sondern begleiten; jede Meinung als wichtig bewerten und die Gruppe entscheiden lassen
**Rolle/Aufgaben zum Ende der Arbeitsphase**
• Zusammenfassung der besprochenen Aspekte und Ergebnisse, ggf. inkl. Überleitung zur nächsten Gruppe<br>• Dokumentation aller Arbeitsschritte (z.B. schriftlich, via Audio, via Foto)
