# Supplementary material for: Feasibility of the interdisciplinary multimodal assessment—The team perspective
Source: Schmerz. 2024 Apr 9;39(6):398–416. [Article in German] doi: 10.1007/s00482-024-00796-z (PMC12627191; doi:10.1007/s00482-024-00796-z)
Supplement: Supplementary file 3 — 3. Bilddateien und Auswertungstabellen Workshop [file 482_2024_796_MOESM3_ESM.pdf]

Online-Zusatzmaterial 3 – Bilddateien und Auswertungstabellen

Dokumentation der Arbeitsphase 1

Medizin

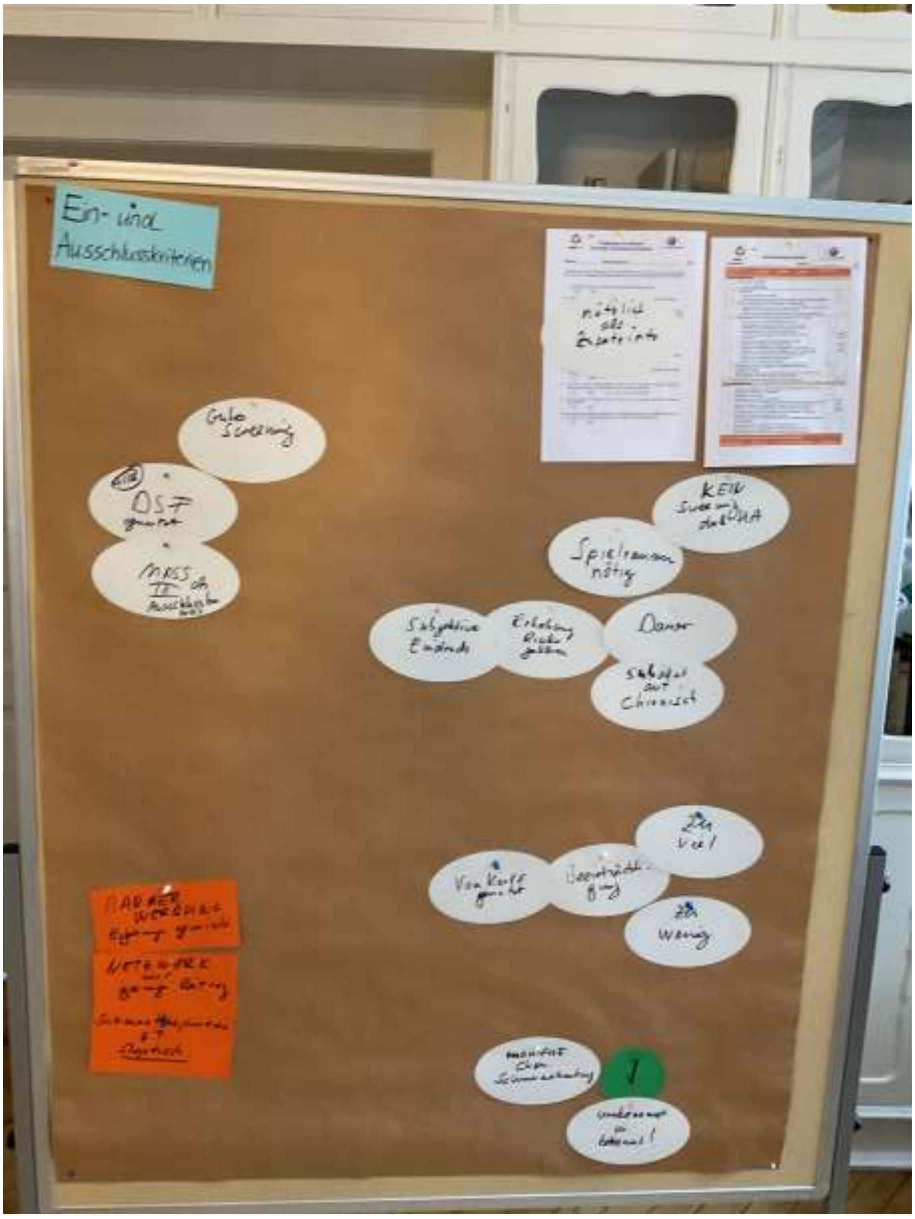

Physiotherapie

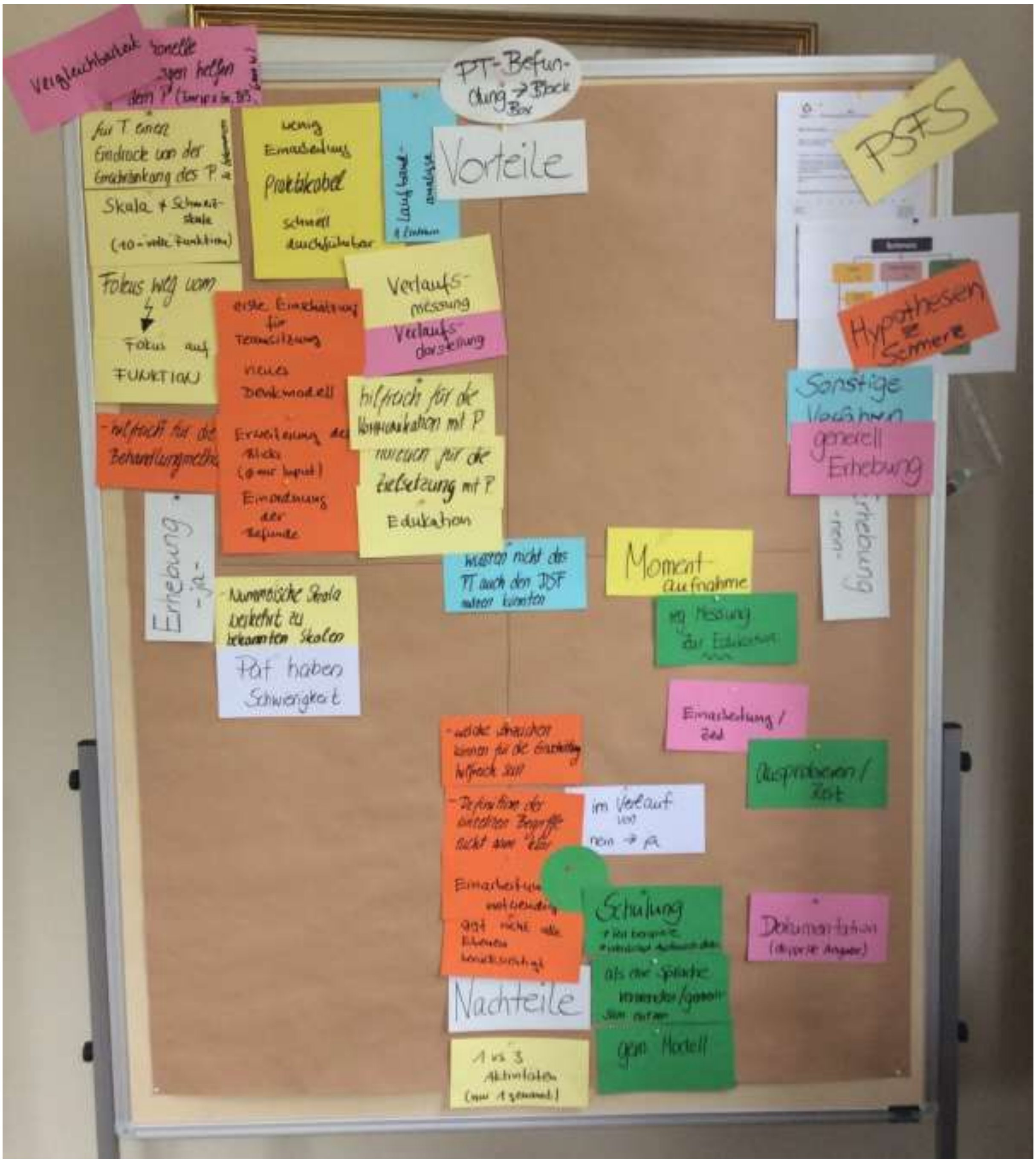

Vor-/ und Nachteile Patientenspezifische Funktionsskala (PSFS)

<table>
<tr><th></th><th>Erhebung -ja-</th><th>Erhebung -nein-</th></tr>
<tr><td>Vorteile</td><td>*für TH einen Eindruck von der Einschränkung des P. zu bekommen
*Skala ungleich Schmerzskala (10=volle Funktion)
*Fokus weg vom Schmerz
*Fokus auf Funktion
*wenig Einarbeitung
*praktikabel
* schnell durchführbar
* Verlaufsmessung
*hilfreich für die Kommunikation mit P.
*nützlich für die Zielstellung mit P.
*Edukation</td><td rowspan="2">Momentaufnahme</td></tr>
<tr><td>Nachteile</td><td>*Numerische Skala verkehrt zu bekannten Skalen → Pat. haben Schwierigkeit</td></tr>
</table>

Vor-/ und Nachteile der Einschätzung zu den vorrangigen Hypothesen zum Schmerz

<table>
<tr><th></th><th>Erhebung -ja-</th><th>Erhebung -nein-</th></tr>
<tr><td>Vorteile</td><td>*erste Einschätzung für Teamsitzung – Teamsitzung ergänzt bzw. ändert teilweise die Einschätzung; ermöglicht bessere Vorbereitung
*neues Denkmodell
*Erweiterung des Blicks (nicht nur Input)
*hilfreich für die Behandlungsmethode
*Einordnung der Befunde</td><td></td></tr>
<tr><td>Nachteile</td><td colspan="2">*welche Anzeichen können für die Einschätzung hilfreich sein
*Definition der einzelnen Begriffe nicht ganz klar (Zusatz: im Verlauf von nicht verständlich → verständlich)
*Einarbeitung notwendig **
*ggf. nicht alle Ebenen berücksichtigt</td></tr>
</table>

Psychologie

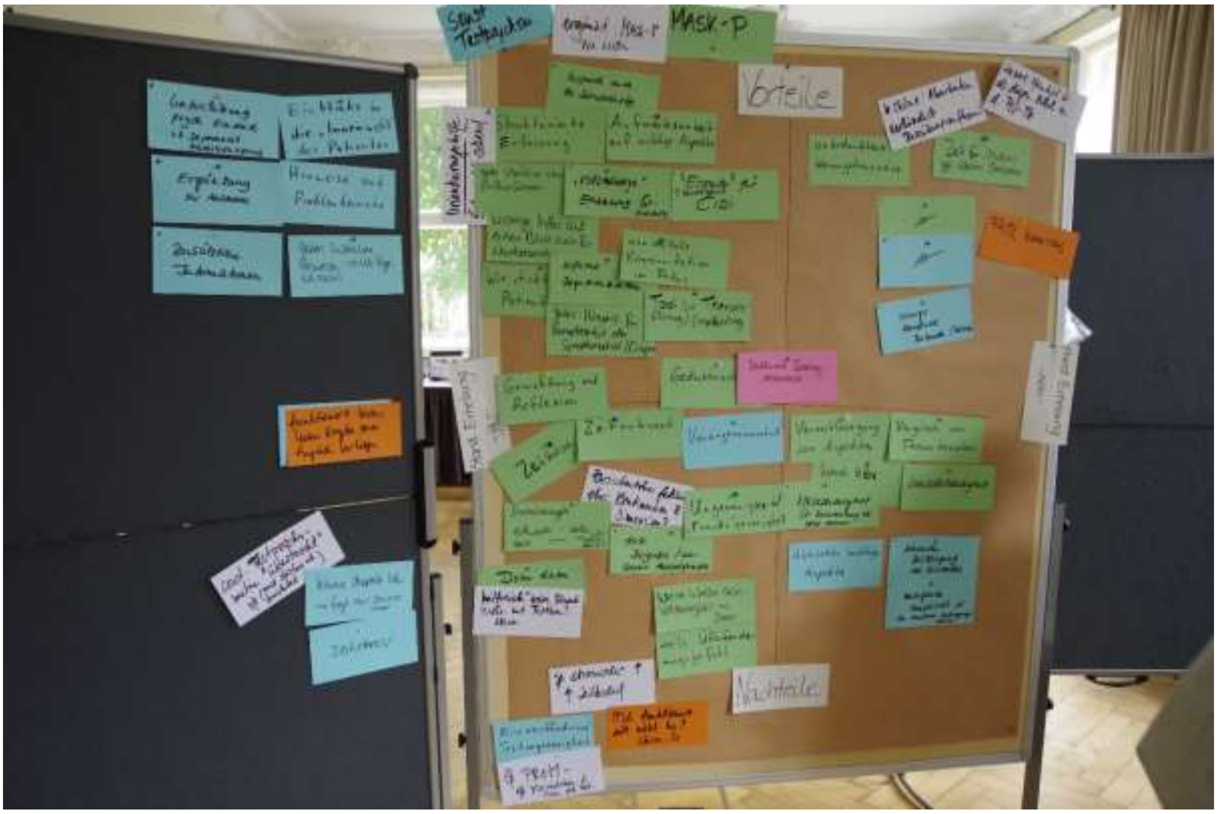

Vorteile standardisierte Erhebung vs. nicht-standardisierte Erhebung im Rahmen der psychologischen Anamnese im IMA

Standardisierte Erhebung -ja- [sowohl MASK-P, als auch sonstige TPV]	standardisierte Erhebung -nein-
Orientierungshilfe/Struktur setzend	unbedarfte Herangehensweise
hilfreich auch bei Berufseinstieg	[kein] sklavisches Abarbeiten/verhindert [sonst] Beziehungsaufbau
Strukturierte Erfassung	innere Offenheit [sinkt]; [innere Offenheit] als diagnostisches Mittel in der Psychotherapie
Aufmerksamkeit auf wichtige Aspekte [richtend]	Zeit für Anderes [,] ggf. offenes Gespräch
guter Überblick über Einflussfaktoren	PHQ kann raus
"vollständige" Erhebung S[chmer]z-verarbeitung	weniger komplexe Befunde (Zeiters[parnis])
"Ersatz" [entspricht] Wichtigkeit für CIDI	[keine]
Wichtige Infos auf einen Blick auch für Nachbehandler	[keine]
Wie "tickt" der Patient	
Hilfreiche Begrifflichkeiten	
nonverbale Kommunikation im Fokus	
guter Hinweis auf Komplexität der Symptomatik/Diagnose	

Tool zur Therapieplanung/Empfehlung	
Gewichtung zur Reflexion	
Gedächtnisstütze	
strukturierte Schmerzanamnese	
[folgende Felder beziehen sich auf Testpsychometrie allgemein]	
Unterstützung psych[ischer] Eindruck, z.B. Depressivität, Katastrophisierung	
Einblick in die Innenwelt des Patienten	
Ergänzung zur Anamnese	
Hinweise auf Problembereiche	
zusätzliche Informationen	
geben zusätzliche Hinweise [Pfeil] wo frage ich nach?	

Nachteile standardisierter und nicht-standardisierter Erhebung

Standardisierte Erhebung -ja- [sowohl MASK-P, als auch sonstige TPV]	standardisierte Erhebung -nein-
Zeitkritisch	Vernachlässigung von Aspekten
Zeitaufwand	Vergessen von Themenblöcken
Voreingenommenheit	fehlende Infos
"Formulierungen" entweder - oder, statt was denkt Patient	Unvollständigkeit
Zwischentöne [in MASK-P] fehlen; eher Kontinuum oder Dimension?	Vielschichtigkeit S[chmer]z-Verarbeitung etc. geht verloren
Ungenauigkeit/Pseudogenauigkeit	Übersehen wichtiger Aspekte
feste Begriffe/sehr starke Ausprägungen [in MASK-P]	fehlende Bestätigung des Eindrucks
Doku dazu	Mangelnde Komplexität für ein komplexes Bedingungsgefüge
[zu Doku ergänzend] hilfreich wäre Eingabe-Maske mit Textbausteinen	
wenn Wunsch nach Vollständigkeit [in der Bearbeitung zur MASK-P] [dann] Stress	
[zu wenn Wunsch…] teils Überforderungsgefühl	
je chronischer [desto mehr] Zeitbedarf	
IMA funktioniert zeitlich nicht bei [zunehmendem] chronischen Schmerz	
sonstige Testpsychometrie "überrascht" oft (meist stärker als berichtet)	
teilweise doppelte Infos [Pfeil] fragt man sowieso	
zeitintensiv	

<u>Arbeitsphase 2</u>

Medizin

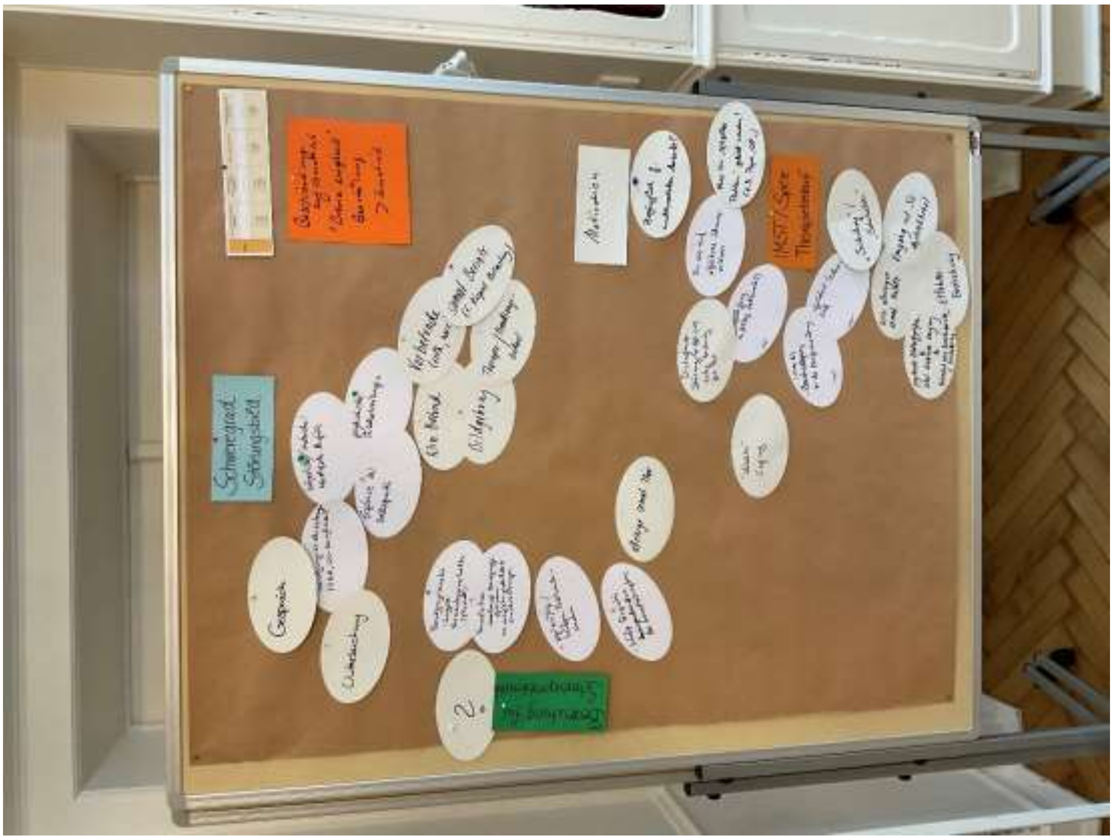

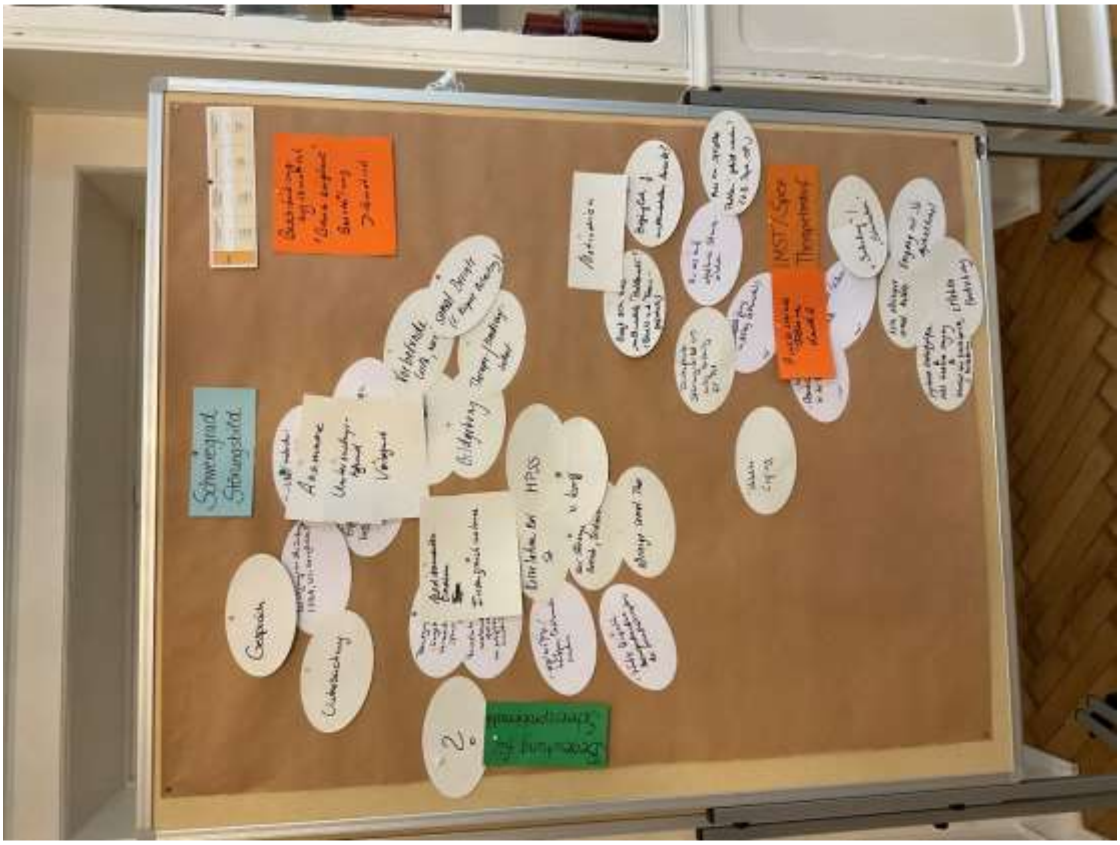

Kriterien „Schweregrad Störungsbild“, „Bedeutung für die Schmerzproblematik“ und „IMST/Spezifischer Therapiebedarf“ – Medizinische (somatische) Perspektive

**Kriterien** Schweregrad Störungsbild [hier ergänzt: „klar“ somatischl]	**Kriterien** Bedeutung für die Schmerzproblematik	**Kriterien** IMST/Spezifischer Therapiebedarf
Gespräch	Bewegungsmuster/-ängste Vermeidungsverhalten (Physisch)	Motivation
Untersuchung<br>Klin[ischer] Befund<br>Bewegungseinschränkungen (FBA [Finger-Boden-Abstand], WS [Wirbelsäulenbeweglichkeit]]<br>Körperliche/motorische/neurologische Ausfälle [in der Diskussion zusammengefasst]	Korrelation messbarer Bewegungsspielraum [mit] subjektive geschilderte[n] Einschränkungen	Zugänglich für multimodalen Ansatz
Ergebnisse der Vordiagnostik Vorbefunde (orth[opädisch] Bildgebung	Regelmäßige/häufige Medikamenteneinnahme	Zeigt sich eine „multimodale Problematik?“ (Bereits v[or] d[em] Teamgespräch
Psychiatrische Zusatzerkrankungen	Häufige Diagnostik Inanspruchnahme des Gesundheitssystems	Diskrepanz Störungsbild und subj[ektive]Bedeutung für Pat[ienten]
Therapie-/Handlungsbedarf	Alleinige somatische Therapie	Hinweis auf affektives Schmerzerleben
Somatische Beeinträchtigung (f[ür] körperl[iche] Belastung	Korrelation Befund und Sz [Schmerz]	Muss ein „spezielles Problem“ gelöst werden (z.B. Psych., OP…)
MPSS<br>v. Korff	Aus Störungs[modell], Beeintr[ächtigung], Leidensdr[uck]	Beeinträchtigung im Alltag (o[der] Hausarbeit
	MPSS<br>v. Korff	„schlechtes“ Coping Umgang mit Sz [Schmerz] dysfunktional
		(drohende) Beeinträchtigung in der Berufsausübung
		Subjektiver Leidensdruck
		„Schulung“/“Edukation“ [im Sinne von Bedarf für]
		Psychosoziale Einflussgrößen [und] fehl[ender] funktion[eller] Umgang [und] Potential über Kenntnisse der SM [*Schmerzmechanismen*] f[ür] Veränderung
		Sz [Schmerz]-Erleben: Bedrohung
**Übergeordnete Kriterien für Wichtigkeit/Sicherheit:**<br>Anamnese<br>Untersuchungsbefund	**Übergeordnete Kriterien für Wichtigkeit/Sicherheit [bei Fokussierung auf**	**Übergeordnete Kriterien für Wichtigkeit/Sicherheit:**<br>Psychosoziale Faktoren deutlich

Vorbefunde	**somatisches Störungsbild]:**<br>Medikamenten-Einnahme<br>Inanspruchnahme/ Arbeitsunfähigkeit<br>Korrelation Befund und Schmerz<br>Aus Störungsmodell result[ierend]: Beeintr[ächtigung], Leidensdruck	Aufgeschlossenheit für Therapie (IMST) [bei Diskussion der Wichtigkeit (s.u.) neu formuliert]<br>Zeigt sich eine „multimodale Problematik?“(Bereits v[or] d[em] Teamgespräch)

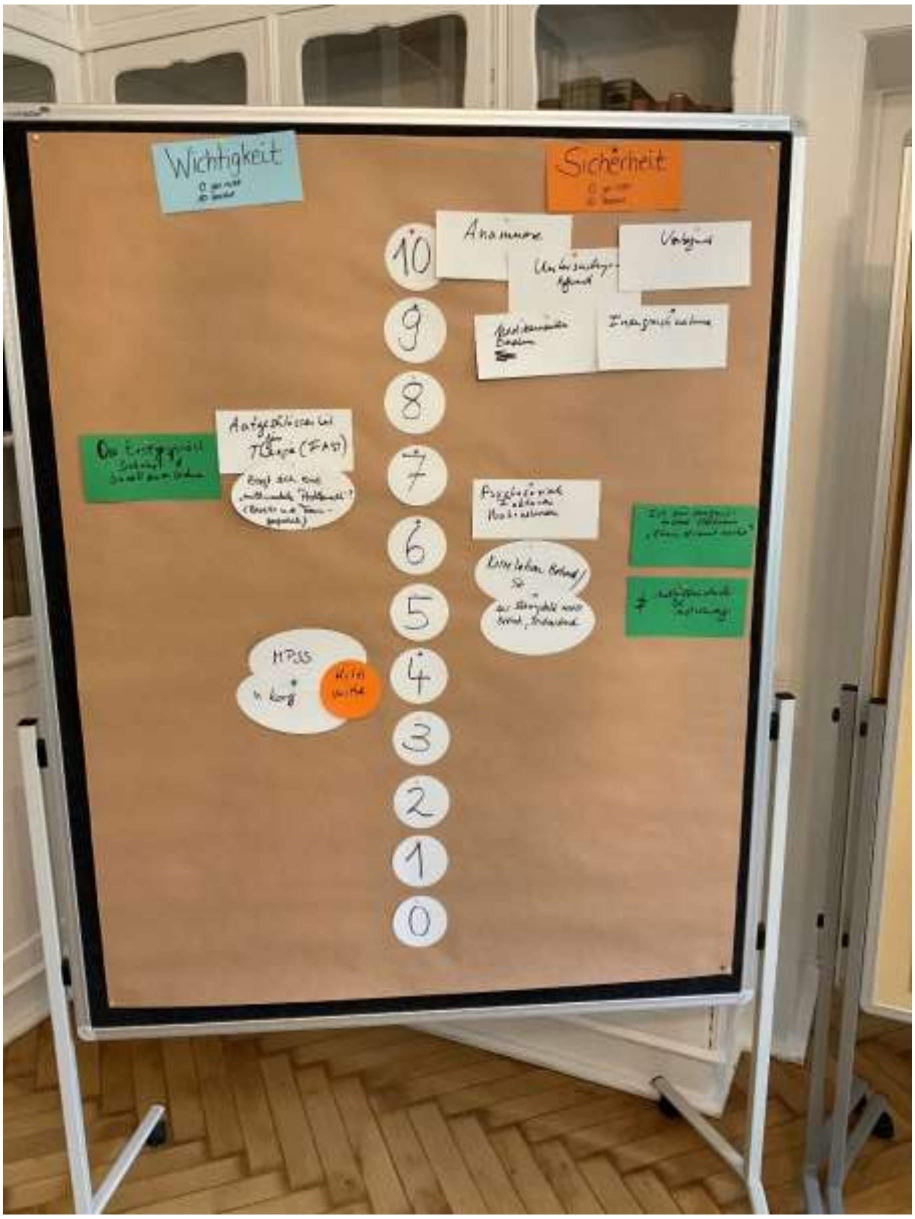

Wichtigkeit/Sicherheit – Medizinische (somatische) Perspektive

<table>
<tr><td colspan="4">Wichtigkeit [W] und Sicherheit [S] ausgewählter Kriterien
[Übergeordnete Kriterien]</td></tr>
<tr><th></th><th>Wichtigkeit</th><th>Sicherheit</th><th></th></tr>
<tr><td>10</td><td>Anamnese
Untersuchungsbefund
Vorbefunde</td><td rowspan="2">Anamnese
Untersuchungsbefund
Vorbefunde
Medikamenteneinnahme
Inanspruchnahme</td><td></td></tr>
<tr><td>9</td><td>Psychosoziale Faktoren wahrnehmen [statt wichtig]</td><td></td></tr>
<tr><td>8</td><td>Medikamenteneinnahme
Inanspruchnahme
Korrelation Befund/Sz [Schmerz]
Aus Störungsbild result[ierend]: Beeintr[ächtigung], Leidensdruck</td><td></td><td></td></tr>
<tr><td>7</td><td>Aufgeschlossenheit für Therapie (IMST)
Zeigt sich eine „multimodale Problematik?“(Bereits v[or] d[em] Teamgespräch)</td><td></td><td>[Ergänzung auf Metaebene:]
Da Erstgespräch Sicherheit Ø [nicht] sinnvoll einzuordnen</td></tr>
<tr><td>6</td><td></td><td>Psychosoziale Faktoren wahrnehmen [statt wichtig]</td><td rowspan="2">[Ergänzung auf Metaebene:]
Ist ein diagnostisches Kriterium:
„Etwas stimmt nicht“ entspricht nicht mehr somatische Dx [Diagnostik] notwendig</td></tr>
<tr><td>5</td><td></td><td>Korrelation Befund/Sz [Schmerz]
Aus Störungsbild result[ierend]: Beeintr[ächtigung], Leidensdruck</td></tr>
<tr><td>4</td><td>MPSS
v. Korff [nur Hilfsmittel]</td><td></td><td></td></tr>
<tr><td>3</td><td></td><td></td><td></td></tr>
<tr><td>2</td><td></td><td></td><td></td></tr>
<tr><td>1</td><td></td><td></td><td>[</td></tr>
<tr><td>0</td><td></td><td></td><td></td></tr>
</table>

Physiotherapie

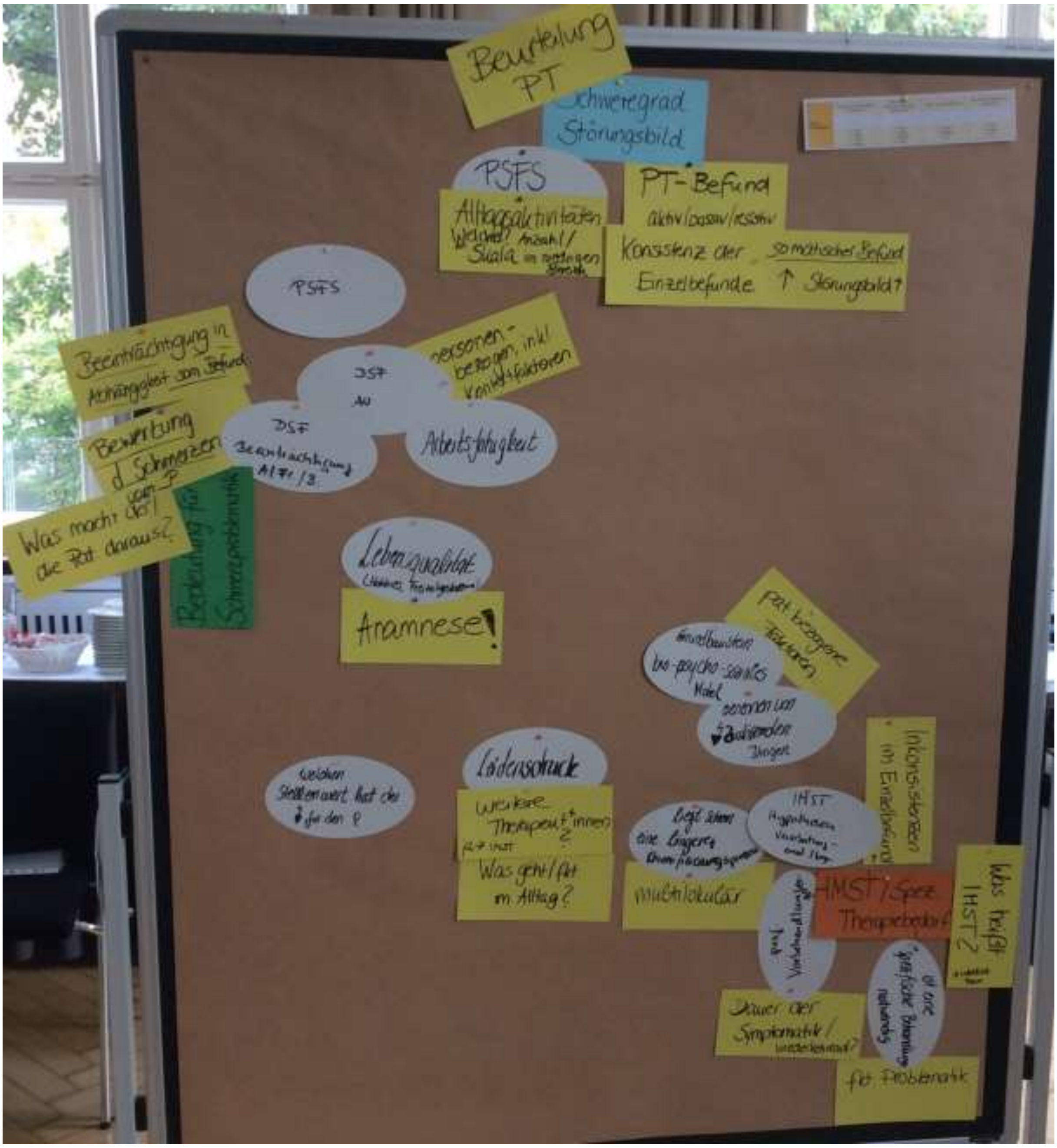

Kriterien „Schweregrad Störungsbild“, „Bedeutung für die Schmerzproblematik“ und „IMST/Spezifischer Therapiebedarf“ –Physiotherapeutische (funktionelle) Perspektive

**Kriterien** Schweregrad Störungsbild [hier ergänzt: „klar“ funktionell]	**Kriterien** Bedeutung für die Schmerzproblematik	**Kriterien** IMST/Spezifischer Therapiebedarf
PSFS<br>- Alltagsaktivitäten – Welche (Art)?<br>- Alltagsaktivitäten – Wofür? Beruf, Freizeit, Alltag	DSF Beeinträchtigung A/Fr/B	spez. Vorbehandlungen [*in der Vorgeschichte, dann eher zu IMST]*
PT-Befund aktiv/passiv/resistiv	DSF AU	Dauer der Symptomatik/wiederkehrend *[auch als Beschreibung für IMST verwendet]*
Konsistenz der Einzelbefunde	Arbeitsfähigkeit *(z.B. „Müssen Stunden reduziert werden?“, „Ist ein 8-Stunden-Tag zu bewältigen?“, „Muss am Arbeitsplatz häufig die Tätigkeit gewechselt werden?“, „Muss ich mich immer wieder krankmelden?“, „Wie entscheidend ist das Arbeiten für die Personen?“)*<br>- personenbezogen, inkl. Kontextfaktoren	
somatischer Befund – Störungsbild hoch	Lebensqualität (Hobbies, Freizeitgestaltung *(Freizeitaktivitäten)*, Freunde *(sozialer Rückzug)*<br><br>Haltung zum Gesundheitsverhalten/ Bewegungsverhalten<br><br>Ressourcen? – funktionell/körperlich, [*Ergänzungen zu Ressourcen auch aus dem Team*]	liegt schon ein längerer Chronifizierungsprozess vor?<br><br>multilokulär
		IMST Hypothesen Verarbeitung emot./kognitiv
		Inkonsistenzen im Einzelbefund [*würde für eine IMST sprechen, auch unterschiedliche Angaben innerhalb der drei Berufsgruppen]*
		Ist eine spezifische Behandlung notwendig? → funktionelle Problematik
PSFS (Schweregrad > Schmerzproblematik)		

	Welchen Stellenwert hat der Schmerz für den Pat.? *(Bedeutung>IMST)*
	Leidensdruck
	Pat.bezogene Faktoren<br>- Grundbaustein bio-psycho-soziales Modell<br>- Benennen von schmerzauslösenden Dingen *[in Bezug auf Bewegungen, Aktivitäten, Druck (mechanisch), Stressfaktoren, Einflussfaktoren auf den Schmerz; als Einschätzung, ob ein bio-psycho-soziales Modell vorliegt]*

**Beschreibung** (zusammenfassend) Schweregrad Störungsbild [hier ergänzt: "klar" funktionell]	**Beschreibung** (zusammenfassend) Bedeutung für die Schmerzproblematik	**Beschreibung** (zusammenfassend) IMST/Spezifischer Therapiebedarf
Beurteilung PT *(orientiert am somatischen Befund)*	Beeinträchtigung des Pat. in Abhängigkeit som. Befund	- keine Beschreibung aus PT-Kleingruppe vorhanden, da Teil vom Teamprozess<br>- mehrfach beschrieben als *"Brauche ich noch eine weitere Person?"*<br>- *"übergeordneter Bedarf", multilokuläre Schmerzen, Länge der Symptomatik (wiederkehrend?), Übertrag in den Alltag nicht gegeben, vorherige Therapien nicht hilfreich (weitere Strategien und Tipps der anderen Berufsgruppen notwendig), fkt. Problematik gering (Störungsbild und Bedeutung für die Schmerzproblematik niedrig und trotzdem hohe Schmerzen --> eher Empfehlung für IMST*<br>- *Unterscheidung zwischen Durchhalter/Vermeider? --> kein zwingendes Kriterium für IMST*

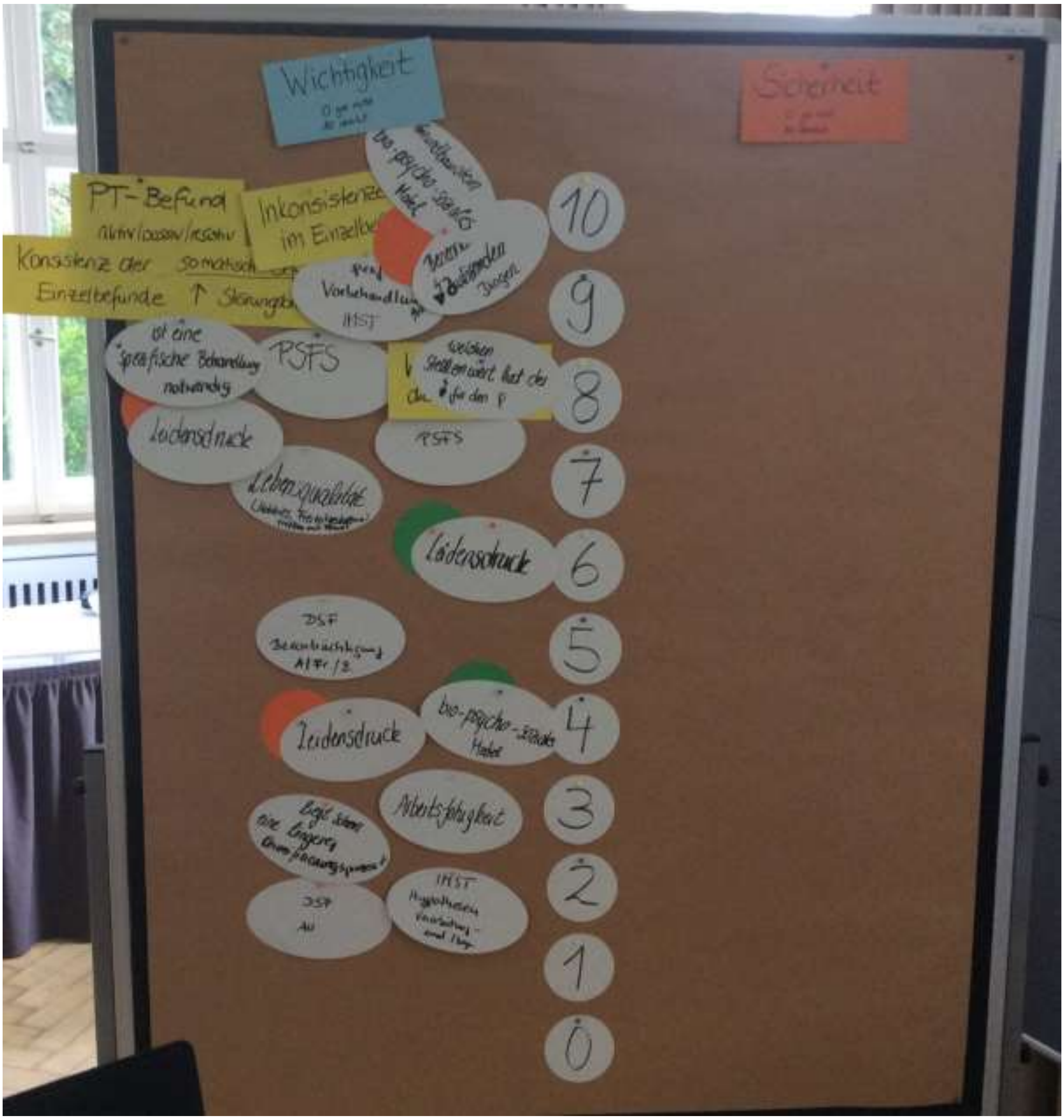
Wichtigkeit
PT-Befund
ist eine spezifische Behandlung notwendig
PSFS
Leidensdruck
Lebensqualität
DSF
Arbeitsfähigkeit
IMST
10
9
8
7
6
5
4
3
2
1
0

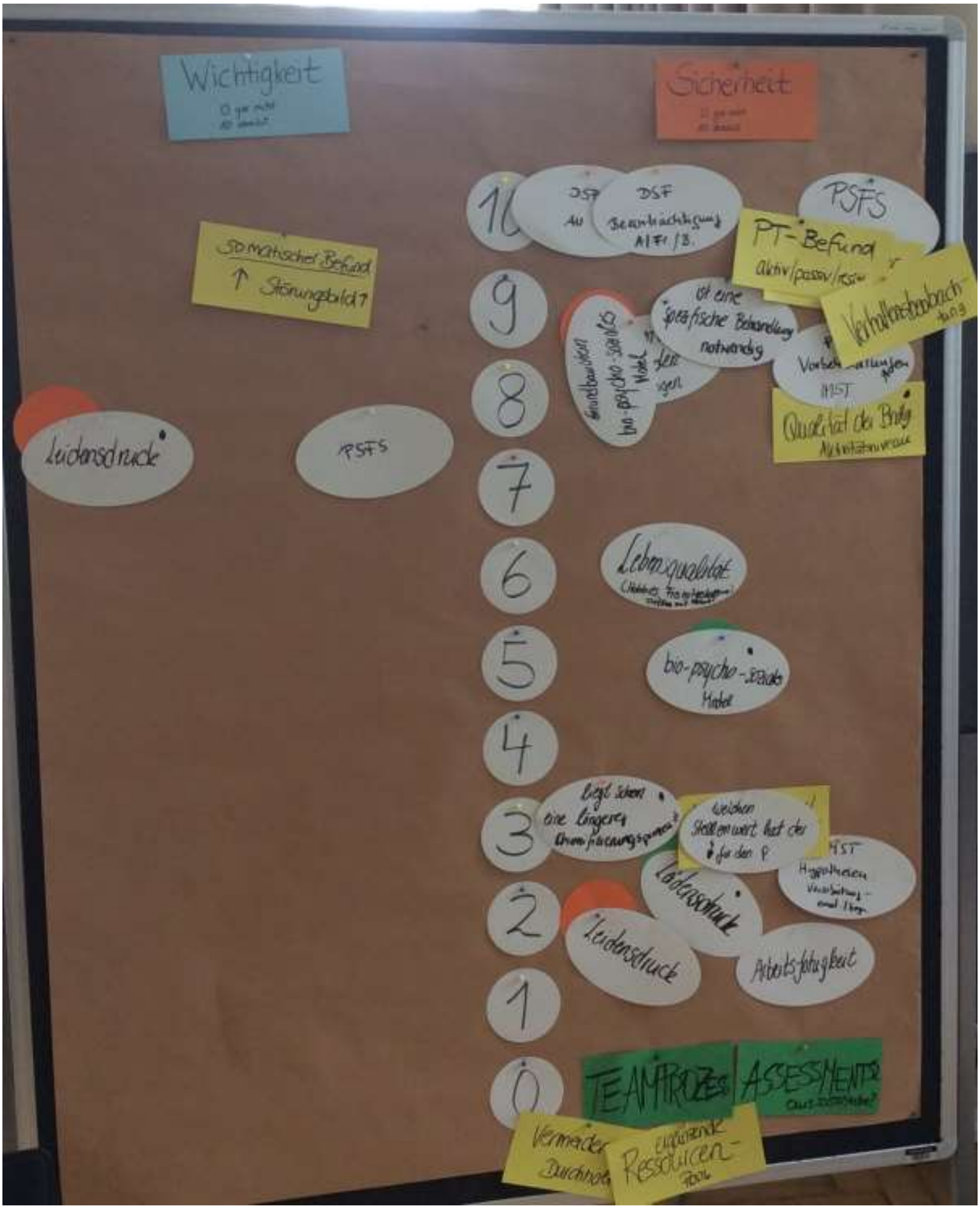
Wichtigkeit
Sicherheit
somatischer Befund
↑ Störungsbild?
Leidensdruck
PSFS
10
9
8
7
6
5
4
3
2
1
0
DSF
AU
DSF
Beeinträchtigung
PSFS
PT-Befund
aktiv/passiv
ist eine spezifische Behandlung notwendig
Verhaltensbeobachtung
bio-psycho-soziales Modell
IMST
Qualität der Bfg
Lebensqualität
bio-psycho-soziales Modell
liegt schon eine längere Chronifizierungsphase
Welchen Stellenwert hat der
für den P
IMST
Hypothesen
Leidensdruck
Leidensdruck
Arbeitsfähigkeit
TEAMPROZESS
ASSESSMENTS
Vermeiden
Durchhalten
Ressourcen-
Pool

Wichtigkeit/Sicherheit – Physiotherapeutische (funktionelle) Perspektive

	Wichtigkeit	Sicherheit
10	*Grundbaustein bio-psycho-soziales Modell *[aus IMST-Bedarf]*<br>*PT-Befund aktiv/passiv/resistiv<br>*Inkonsistenzen im Einzelbefund	*DSF AU [*aus dem DSF zu entnehmen]*<br>*DSF Beeinträchtigung A/Fr/B [*aus dem DSF zu entnehmen]*<br>*PT-Befund aktiv/passiv/resistiv [darunter: Inkonsistenzen im Einzelbefund) + Konsistenz der Einzelbefunde]<br>*Neu: Verhaltensbeobachtung<br>*PSFS
9/10	*Bennen schmerzauslösender Dinge	
9	*Konsistenz der Einzelbefunde<br>*somatischer Befund – Störungsbild hoch<br>*spez. Vorbehandlung (gehört zu IMST)	*Ist eine spezifische Behandlung notwendig?
8/9		*Grundbaustein bio-psycho-soziales Modell *[aus IMST-Bedarf]*<br>*Benennen von schmerzauslösenden Dingen
8	*PSFS *[Störungsbild]*<br>*ist eine spezifische Behandlung notwendig<br>*Welchen Stellenwert hat der Schmerz für den P.?/Was macht der/die Pat. daraus	*spez. Vorbehandlung; Neu: Qualität der Behandlung, Aktivitätsniveau
7/8	*PSFS *[Bedeutung für die Schmerzproblematik]*<br>*Leidensdruck	
7	*Lebensqualität (Hobbys, Freizeitgestaltung, Treffen mit Freunden)	
6	*Leidensdruck	*Lebensqualität (Hobbys, Freizeitgestaltung, Treffen mit Freunden)
5	*DSF Beeinträchtigung A/Fr/B	*bio-psycho-soziales Modell [aus Bedeutung für Schmerzproblematik]
4	*bio-psycho-soziales Modell [aus Bedeutung für Schmerzproblematik]	
3	*Arbeitsfähigkeit<br>*liegt schon ein längerer Chronifizierungsprozess vor	*liegt schon ein längerer Chronifizierungsprozess vor<br>*Welchen Stellenwert hat der Schmerz für den P.?/Was macht der/die Pat. daraus
2	*DSF – AU<br>*IMST Hypothesen: Verarbeitung emot./kognitiv	*Leidensdruck<br>*IMST Hypothesen Verarbeitung emot./kognitiv
1		*Arbeitsfähigkeit
0		

Psychologie

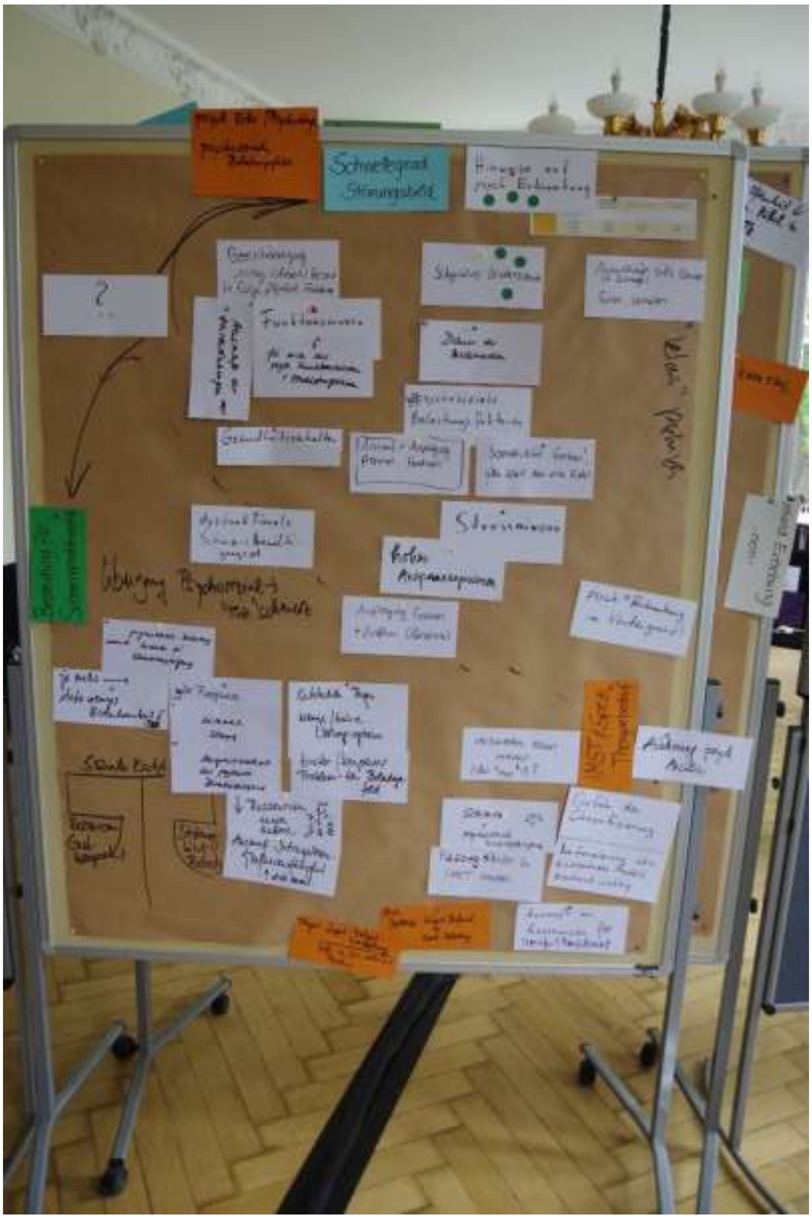

Kriterien „Schweregrad Störungsbild“, „Bedeutung für die Schmerzproblematik“ und „IMST/Spezifischer Therapiebedarf“ – Psychologische (psychosoziale) Perspektive

Schweregrad Störungsbild [hier ergänzt: "klar" Psychisch]	Bedeutung für die Schmerzproblematik	IMST/Spezifischer Therapiebedarf
[eindeutig; absteigend von Nähe in die Distanz; dieselbe Ebene in einem Feld]	Psychosoziale Belastung wesent[licher] Mediator für Schmerzausprägung	verschiedene Ebenen relevant oder "nur" 1?
psychosoziale Belastungsfaktoren<br><br>Hinweise auf psychische Erkrankungen	sozialer Kontext: [Waage zwischen] Ressourcen/Gesundheitskompetenz [und] Störungsbild/Belastung	[Waage] Schmerz [und] psychosoziale Beeinträchtigung
subjektiver Leidensdruck Auswirkungen auf das Denken (d[en] Schmerz); Fühlen und Verhalten Beeinträchtigung Alltag/Arbeit/Freitzeit [dazu sortiert:] Funktionsniveau; für mich eher Funktionsniveau und Erschöpfungserleben; Ausmaß der Einschränkungen	je mehr [der Ressourcen; siehe Waage] desto weniger Bedeutsamkeit f[ür] S[chmerz]P[roblematik]	Passung Person zu IMST Inhalten
Dauer der Beschwerden	[Bezug zu Prognosen- gegenüber gestellt]	Gefahr der Chronifizierung
Biopsychosoziale Belastungsfaktoren [hier sortiert:] Anzahl [und] Ausprägung psychol[ogischer] Faktoren; somatische Faktoren/was spielt [unleserlich]eine Rolle?	Gute Prognose [dazu sortiert:] zeitnahe Lösung; Abgrenzbarkeit der psychosoz[ialen] Beeinträchtigung	Informierung über biopsychosoziales Schmerzmodell erscheint wichtig
[auf derselben Ebene aber zu Beeinträchtigung sortiert:] Gesundheitsverhalten	schlechte Prognose [dazu sortiert:] wenig/keine Lösungsoption; breites/komplexes Problem- bzw. Belastungsfeld	Ausmaß an Ressourcen für den Transfer
Stressniveau/hohes Anspannungsniveau	[zusammenfassend zur Prognose:]	[konkret für spezifischen Therapiebedarf ausgewiesen:]
[direkt im Übergang von Schweregrad Störungsbild zu Bedeutung für die Schmerzproblematik:]	[wenig] Ressourcen; innere, äußere; "wie ist der Zugang dazu"	Ablehnung psychische Ansätze [meint ANDERE Angebote als Psycho]
Dysfunktionale Schmerzbewältigungsart	Ausmaß Introspektions- und Reflexionsfähigkeit -[je mehr] desto besser-	psych[ische] Erkrankung im Vordergrund
Ausprägung Faktoren [und] Einfluss (Beispiele)	[Prognose und Gewichtung der Ressourcen reicht zu IMST/Spezifischer Therapiebedarf]	
[Metaebene: Übergang Psychosozial -->"rein Schmerz" zu Bedeutung für die Schmerzproblematik		[RM Ärzte aus MDI] Dieskrepanz körperl[icher] Befund vs. emot[ionale] Belastung

		[RM Physio aus MDI:] Input-Output-Verarbeitung: Bitte in Zus[ammen]-Arbeit mit Psychos

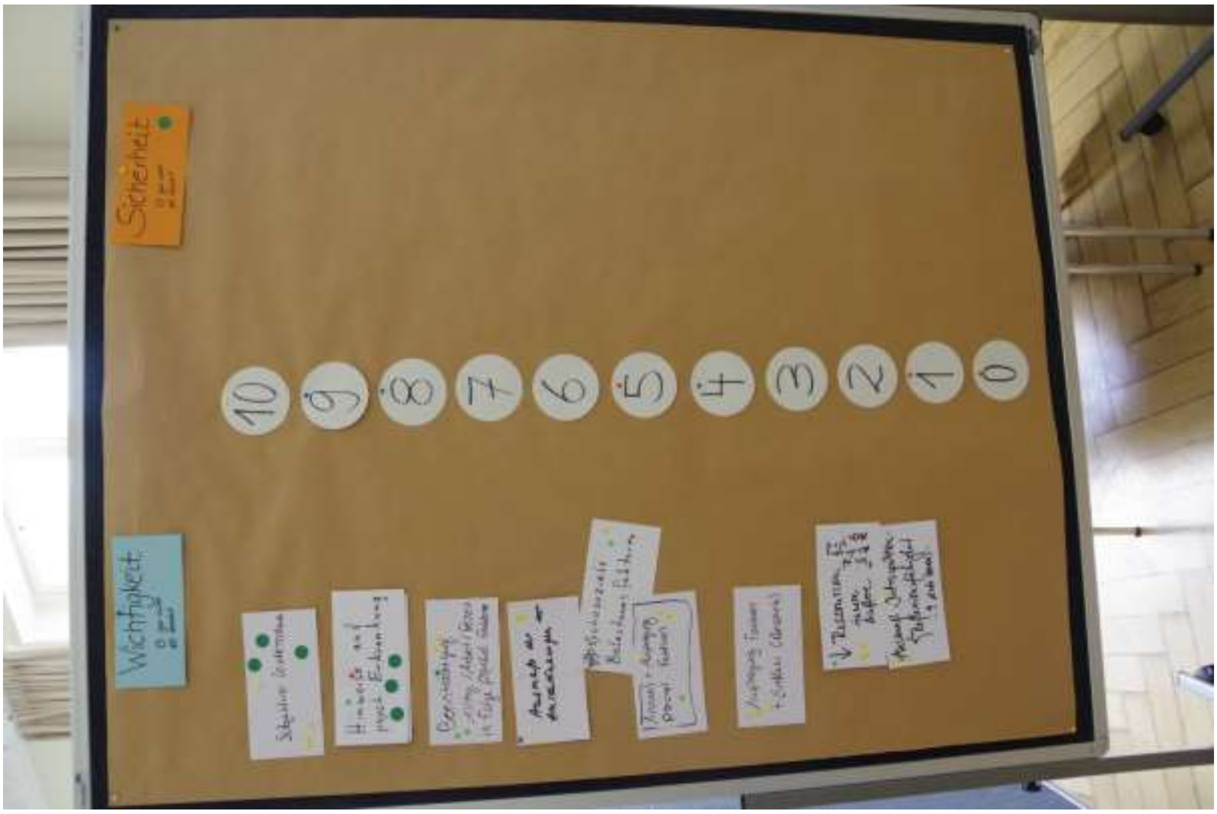

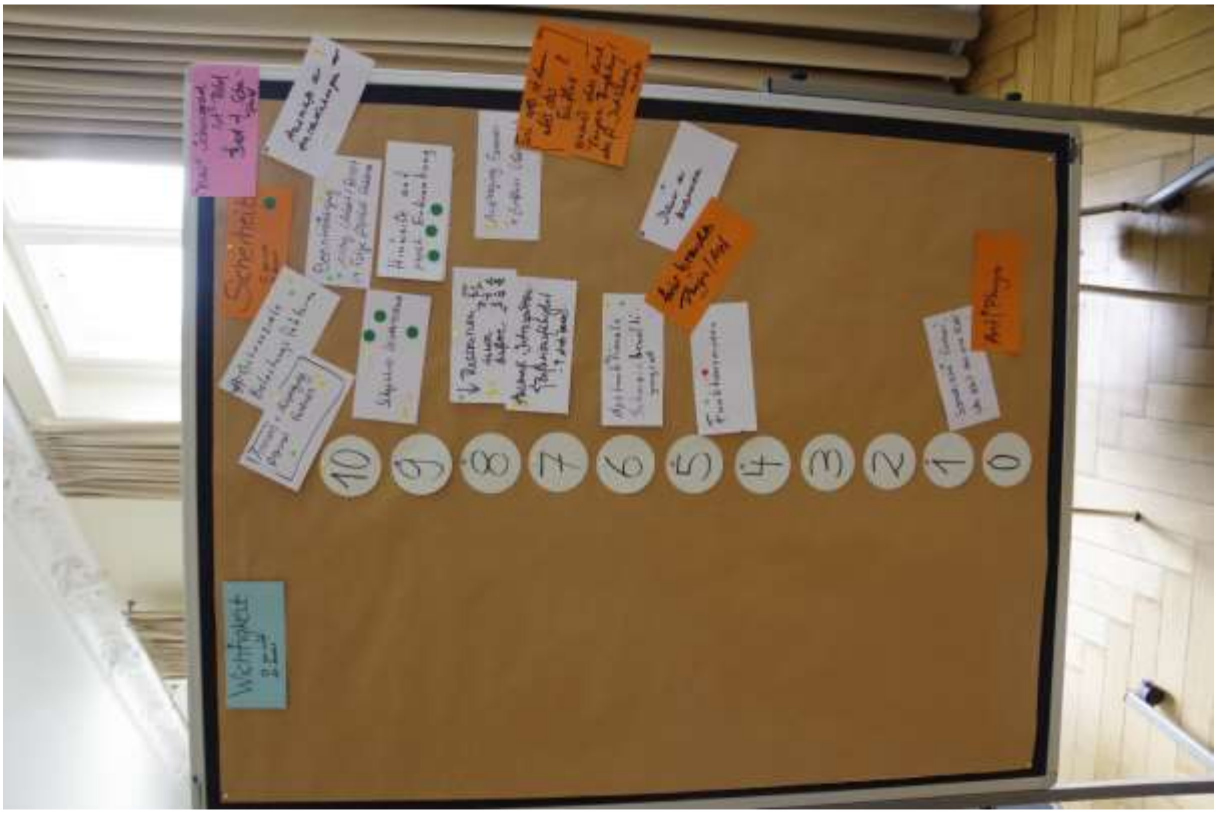

Wichtigkeit/Sicherheit – Psychologische (psychosoziale) Perspektive

Wichtigkeit [W] und Sicherheit [S] ausgewählter Kriterien<br>["nur" Schweregrad Störungsbild und Bedeutung für die Schmerzproblematik]			
	Wichtigkeit	Sicherheit	
10	Subjektiver Leidensdruck	Anzahl [und] Ausprägung psychol[ogischer] Faktoren; Biopsychosoziale Belastungsfaktoren; Beeinträchtigung Alltag/Arbeit/Freizeit in Folge psychologischer Faktoren; Ausmaß der Einschränkungen;	
9	Hinweise auf psychische Erkrankung	Subjektiver Leidensdruck; Hinweise auf psychische Erkrankung	
8	Beeinträchtigung Alltag/Arbeit/Freizeit in Folge psychologischer Faktoren	[weniger] Ressourcen, innere, äußere, "wie ist der Zugang dazu"; Ausprägung Faktoren [und] Einfluss (Beispiele); Ausmaß Introspektions- und Reflexionsfähigkeit -[je mehr] desto besser-	[ergänzung auf Metaebene:] Wie groß ist dann aber der Einfluss? Genauer eher durch längere Begleitung/aber für Indikation reichts
7	Ausmaß der Einschränkungen		
6	Biopsychosoziale Belastungsfaktoren	dysfunktionale Schmerzbewältigungsart [war vorher bei Wichtigkeit nicht dabei!]	[Ergänzung auf Metaebene:] Hier brauchts Physio/Arzt
5	Anzahl [und] Ausprägung psychol[ogischer] Faktoren	Dauer der Beschwerden, Funktionsniveau	[Ergänzung auf Metaebene:] Hier brauchts Physio/Arzt
4	Ausprägung Faktoren [und] Einfluss (Beispiele)		
3			
2	[weniger] Ressourcen, innere, äußere, "wie ist der Zugang dazu"		
1	Ausmaß Introspektions- und Reflexionsfähigkeit -[je mehr] desto besser-	Somatische Faktoren, was spielt [unleserlich]eine Rolle?	[ergänzung auf Metaebene:] Physio/Arzt
0			

Arbeitsphase 3

Kriterien Therapieempfehlung

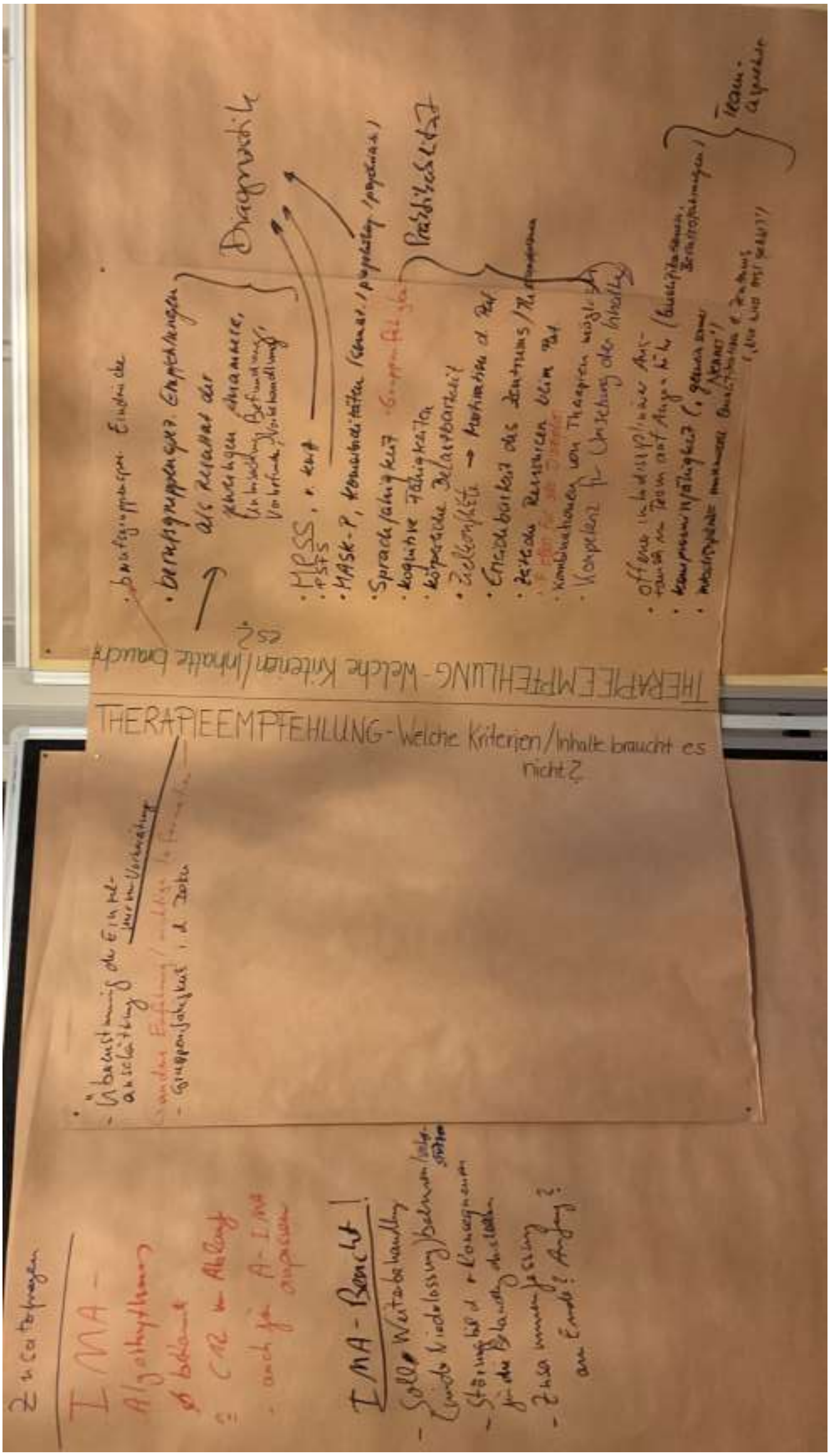

Teamsitzung

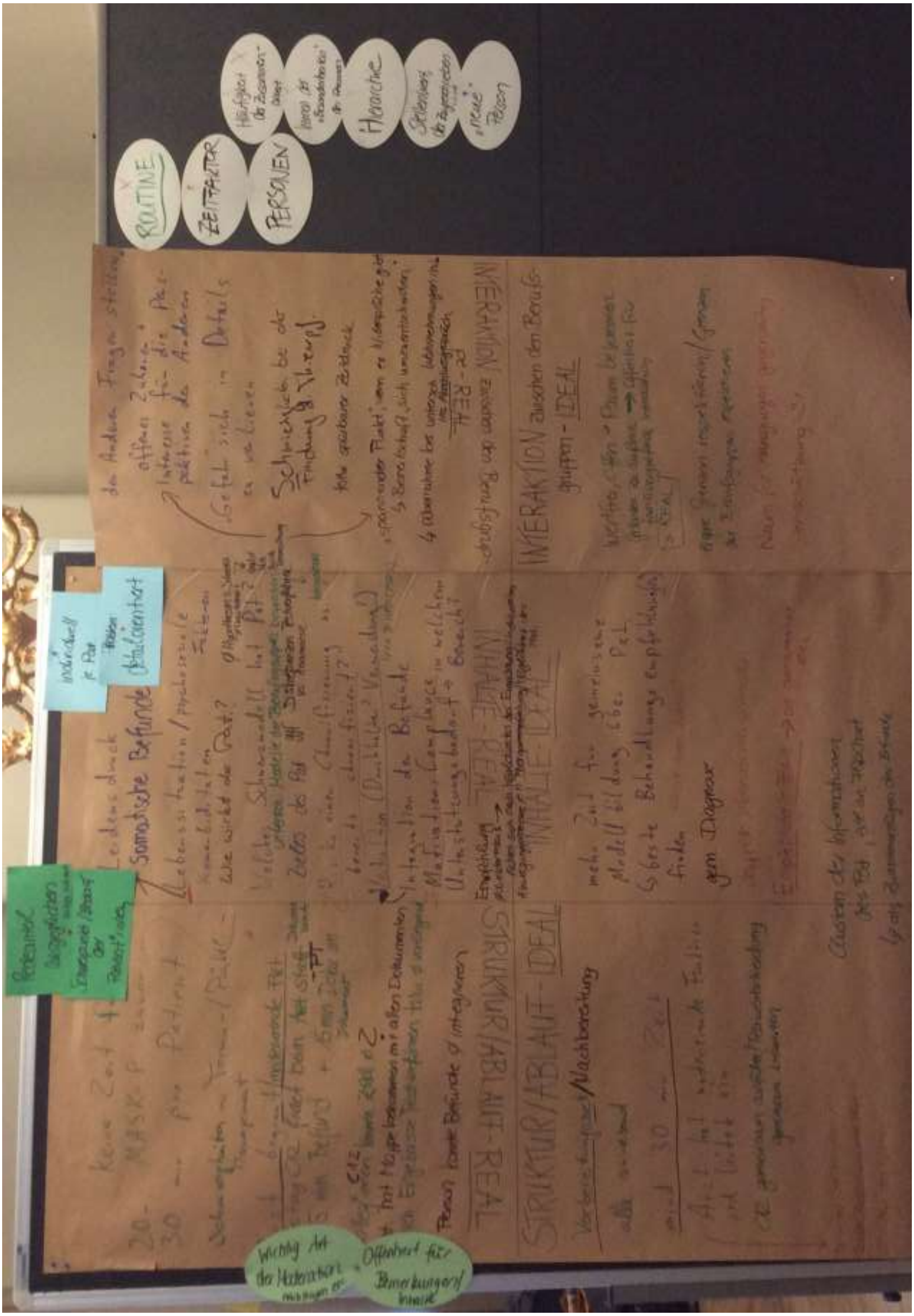

Teamsitzung - Interaktion

**Interaktion** zwischen den Berufsgruppen im Team<br>-**REAL**-	**Interaktion** zwischen den Berufsgruppen im Team<br>-**IDEAL**-
den anderen Fragen stellen [*u.a. zur Wahrnehmung der Patient*innen]*	s. REAL *(u.a. Offenheit, Respekt)*
"Gefahr“ sich in Details zu verlieren<br>“im Kreis drehen“	
offenes Zuhören+Interesse für die Perspektiven der Anderen [Respekt vor der Kompetenz des anderen, Wertschätzung]	wertfrei, wertschätzend, offen → Raum bekommen Gedanken zu äußern *(auch bei Abweichung der eigenen Gedanken)* → Offenheit für fachübergreifende Interaktion
	Raum für Anregungen gegenseitig *(fachübergreifend)*
Schwierigkeiten bei der Findung der Therapieempfehlung	
teilweise spürbarer Zeitdruck [führt dazu, dass das Gespräch „verbal“ limitiert wird – „müssen jetzt mal zum Punkt kommen“; „Zeitdruck spielt immer eine Rolle, teilweise wird dadurch das Gespräch unterbrochen, da noch offene Punkte wie Dokumentation etc. anstehen“]	
„spannender Punkt“, wenn es Widersprüche, ganz unterschiedliche Meinungen gibt, u.a. „Bereitschaft sich um zu entscheiden“ [z.B. aufgrund von mehr Informationen, Pat. sich anders geäußert, schlüssige Schlussfolgerung; Einlassen auf die Meinung des Anderen]<br>--> Übernahme bei untersch. Wahrnehmungen mit ins Abschlussgespräch"	
	eigene Grenzen respektieren/Grenzen der Berufsgruppen respektieren
**Einfluss auf den Teamprozess - Übergeordnet**	
• **Routine**<br>• **Zeitfaktor** *(Zeitdruck teilweise spürbar bzw. Relevanz für eigene Themen wird ggf. nicht gesehen, kein Raum für Unsicherheit in der eigenen Einschätzung)*<br>• **Personen** *(Charakter/Persönlichkeiten)*<br>- Häufigkeit der Zusammenarbeit<br>- Kennen der „Besonderheiten“ der Personen *(Eigenschaften)*<br>- Stellenwert, der *einer Person* zugeschrieben wird<br>- „neue“ Person<br>• **Hierarchie** *(in Bezug auf die Stellung der eigenen Person, u.a. auch die Kultur/der Umgang in der Einrichtung)*	

Abschlussgespräch

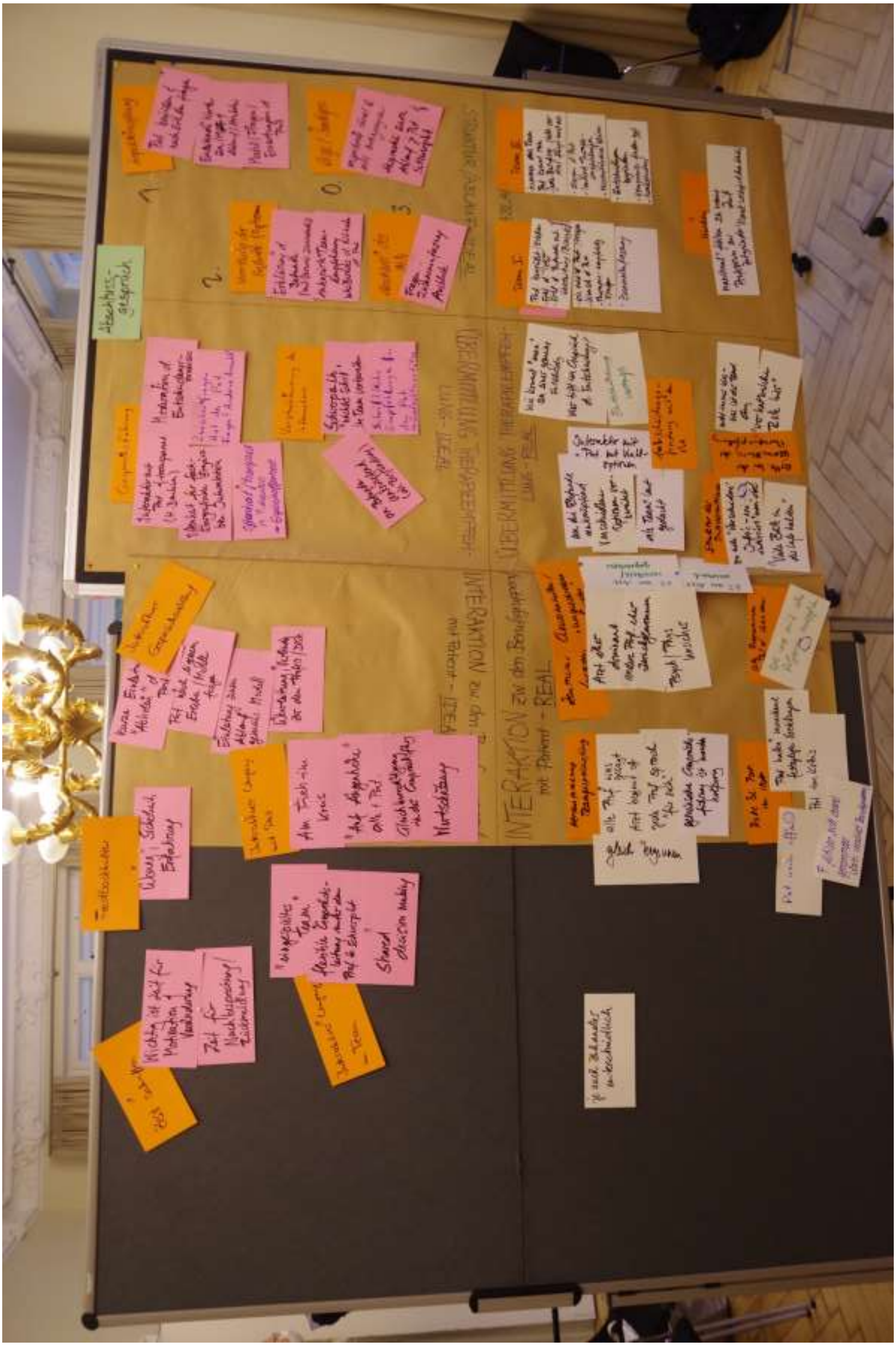

Abschlussgespräch – Struktur/Ablauf

**STRUKTUR/ABLAUF** zwischen den Berufsgruppen/mit Patient -**REAL**-	**STRUKTUR/ABLAUF** zwischen den Berufsgruppen/mit Patient -**IDEAL**-
Team 1 [übergeordnet zu folgenden]	0. Orga[nisation]/Sonstiges [übergeordnet zu folgenden]
Pat[ient] begrüßt/Erleben [erfragt]	ansprechende räuml[iche] & zeitl[iche] Bedingungen
Erkl[ärung] IMST	Absprache zum Ablauf je Pat[ient] [und] Schwerpunkt
Erkl[ärung] d[er] Befunde mit Überleitung/Bezug zueinander	1. Gesprächseröffnung [übergeordnet zu folgenden]
Wie sieht d[er] Pat[ient das]/Fragen	Pat[ient] begrüßen [und] nach Erleben fragen
Wunsch d[es] Pat[ienten]	Einleitende Worte zu M[it]A[rbeitern] [und] Ablauf/Struktur
Therapie-empfehlung	Modell/Fragen/Erwartungen d[es] Pat[ienten]
Fragen	2. Vermittlung der Befunde [und] Optionen
Zusammenfassung	Erklärung d[er] Befunde (mit Verweis zueinander)
Team 2 [übergeordnet zu folgenden]	integrierte Teamempfehlungen unter Berücks[ichtigung] d[er] Wünsche d[es] Pat[ienten]
Kommen aus Team	3. Abschluss des IMAs [übergeordnet zu folgenden]
Pat[ient] kommt rein	Fragen
jede Berufsgruppe stellt sich vor, Arzt fängt meist an	Zusammenfassung
Fragen d[es] Pat[ienten]	Ausblick
lenkend Therapieempfehlungen	
Missverständnisse klären	
Entscheidungen begründen	
Kompromisse finden g[e]g[ebenen]f[alls]	
Verabschiedung	
Hürden [übergeordnet zu folgenden]	
manchmal fehlten Professionen aus Zeitgründen	
zu wenig Zeit	
konnte wenig stehen bleiben	

Abschlussgespräch – Interaktion

**INTERAKTION** zwischen den Berufsgruppen/mit Patient -**REAL**-	**INTERAKTION** zwischen den Berufsgruppen/mit Patient -**IDEAL**-
je nach Behandler unterschiedlich	
Herausforderung Teamgesprächsführung [übergeordnet zu folgenden]	Zeit schaffen [übergeordnet zu folgenden]
alle Prof[essionen haben] was gesagt; Arzt beginnt oft	Wichtig ist Zeit für Motivation und Veränderung
jede Prof[ession] sprach für sich	Zeit für Nachbesprechung/Rückmeldung
gemeinsame Gesprächsführung ist manchm[al] holperig	
[bei uns haben die Professionen] gleich begonnen	
Zentrumshierarchie /Gewohnheiten/"ungeschriebene Gesetze" [übergeordnet zu folgenden]	Interaktiver Umgang im Team [übergeordnet zu folgenden]
Arzt eher dominant	"eingespieltes" Team
Andere Prof[essionen] eher zurückgenommen	flexible Gesprächsleitung unter den Prof[essionen] [entspricht] Schwerpunkt
Psych[ologe]/Physio[therapeut] unsicher	Shared decision making
z.T. nur Arzt anwesend	
z.T. nur Arzt vermittelt/gesprochen	
Rolle d[es] Pat[ienten] im IMA [übergeordnet zu folgenden]	Feedbackkultur [übergeordnet zu folgenden]
Pat[ienten] eher offen	[erfordert] Übung, Sicherheit, Erfahrung
Pat[ienten] hatten manchmal festgelegte Vorstellungen	
Pat[ienten] im Kreis	
P[atienten] fühlen sich ernst genommen (Zeit, verschied[ene] Berufsgruppen)	
	Interaktiver Umgang mit Patient [übergeordnet zu folgenden]
	Am Tisch in einem Kreis
	"auf Augenhöhe" alle [und] Pat[ient]
	Gleichberechtigung in der Gesprächsführung
	Wertschätzung
	Interaktiver Gesprächsablauf [übergeordnet zu folgenden]
	Kurze Einleitung, "Abholen" d[es] Pat[ienten]
	Pat[ient] nach eigenem Erleben/Modell fragen
	Einleitung zum Ablauf gemeins[ames] Modell
	Überleitung, Verbindung zw[ischen] den Profess[ionen]/Disz[iplinen]

Abschlussgespräch – Übermittlung Therapieempfehlung

**ÜBERMITTLUNG THERAPIEEMPFEHLUNG** zwischen den Berufsgruppen/mit Patient -**REAL**-	**ÜBERMITTLUNG THERAPIEEMPFEHLUNG** zwischen den Berufsgruppen/mit Patient -**IDEAL**-
Für Entscheidungsfindung mit Pat[ienten] [übergeordnet zu folgenden]	Gesprächsführung [übergeordnet zu folgenden]
an die Befunde anknüpfend	Interaktiv mit Pat[ient] [und] transparent (l[au]t[es] Denken)
Verschiedene Optionen [vorher im Team] vorbereitet	Offenheit für Fachübergreifende Ergänz[ungen] bzw. Interaktion
als Team laut gedacht	Offenheit/Transparenz im P[atienten]-Gespräch [ergibt] Ergebnisoffenheit
Interaktiv mit Pat[ienten] mit Wahloptionen	Moderation d[es] Entscheidungsprozesses
Wer trifft im Gespräch d[ie] Entscheidung?	Zwischenfragen: Hat der Pat[ient] Fragen? Andere Ansicht?
Entscheidung [manchmal] vertagt	Vorstrukturierung der Information [übergeordnet zu folgenden]
Struktur der Infovermittlung [übergeordnet zu folgenden]	an Befunde ankúpfend (als Begründung)
zu viele "verschiedene" Infos- wie integriert man das?	Schwerpunkte, "nächster Schritt" im Team vorbereiten
"viele Bälle in der Luft halten"	Schriftliche Empfehlungen für den Pat[ient] [erzeugt] Gedächtnisstütze
Rolle bei der Vermittlung der Therapieempfehlung [übergeordnet zu folgenden]	
nicht immer klar- wer ist der Bad Guy	
"wer hat welche Rolle hier"	
