# Supplementary material for: Feasibility of the interdisciplinary multimodal assessment—The team perspective
Source: Schmerz. 2024 Apr 9;39(6):398–416. [Article in German] doi: 10.1007/s00482-024-00796-z (PMC12627191; doi:10.1007/s00482-024-00796-z)
Supplement: Supplementary file 4 — 4. Übersicht Arbeitsphasen 1 bis 3 [file 482_2024_796_MOESM4_ESM.pdf]

Online-Zusatzmaterial 4 – Übersicht Arbeitsphasen 1 bis 3

Arbeitsphase 1-3: Zielsetzung und Fragestellung, Workshop-Methode (konkrete und inhaltliche Umsetzung)

	Zielsetzung und *Fragestellung(en)*	Workshop-Methode	Konkrete Umsetzung
**Arbeitsphase 1**<br>berufsgruppen-spezifisch	Sicherheit im Vorgehen bei der Beurteilung der Ein- und Ausschlusskriterien sowie der Hauptschmerzlokalisation und -diagnose<br><br><u>Ein- und Ausschlusskriterien:</u><br>• *Ist die Formulierung des Kriteriums 2 („in kürzeren Phasen innerhalb der letzten beiden Jahre mehrfach auftretend“) zur Schmerzdauer klinisch geeignet?*<br>• *Gab es Auffälligkeiten/Schwierigkeiten in der Beurteilung im Studienverlauf?*<br>• *War B4 (Screening Fragebogen für die Patienten) hilfreich bei der Beurteilung von vorliegenden Chronifizierungsrisiken?*<br><br><u>Hauptschmerzlokalisation und –diagnose:</u><br>• *Wie wurde die Hauptschmerzlokalisation festgelegt?*<br>• *Welche Angaben/Informationen wurden dafür genutzt?*<br>• *Welche Problemkonstellationen bei der Kodierung gab es?*<br>• *Wie wurde die Hauptschmerzdiagnose festgelegt (insbesondere, wenn keine F45.41 oder primär psychiatrische Diagnose vorlag) – im Rahmen des Teamprozesses oder durch ärztliche Einschätzung?*	**Einzel-/ Gruppenarbeit Schmerzmedizin**<br><br>**Kartenfrage**	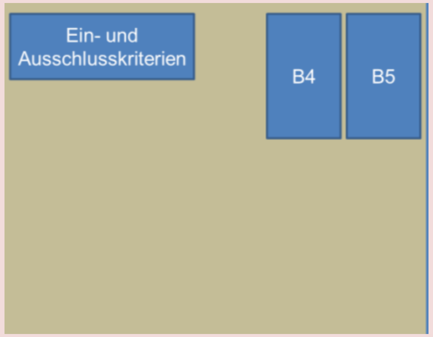<br>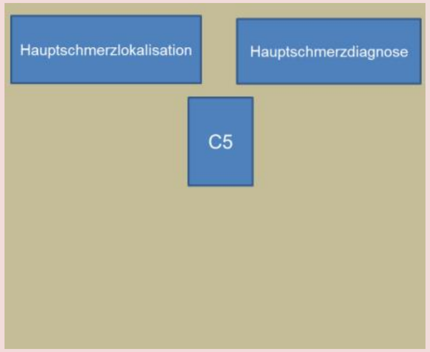
	Nutzen der psychometrischen Testverfahren (C9, DSF inkl. PCS) und der MASK-P (C11) für den eigenen Entscheidungsprozess im Hinblick auf die psychologische Beurteilung und für den Teamprozess	**Einzel-/ Gruppenarbeit Psychologie bzw. Physiotherapie**	• General: standardisierte Erhebung<br>• MASK-P<br>• Sonst. PAIN2020-Testpsychometrie

<table>
<tr>
<td></td>
<td>

Wie praktikabel (Vor- und Nachteile) sind die verwendeten psychometrischen Testverfahren, insbesondere SKID und MASK-P?
Welchen Nutzen haben die psychometrischen Testverfahren und der MASK-P für den Entscheidungsprozess?

Nutzen der PSFS und Hypothesen zum Schmerz für den eigenen Entscheidungs-prozess im Hinblick auf die physiotherapeu-tische Beurteilung und für den Teamprozess

Wie praktikabel (Vor- und Nachteile) sind die im IMA eingesetzten Verfahren (1) Patientenspezifische Funktionsskala und (2) Hypothesen zum Schmerz und (3) sonstige Verfahren, die von den Therapeut:innen angewendet wurden?
Welchen Nutzen haben die zuvor genannten Verfahren hinsichtlich des physiotherapeutischen Entscheidungsprozess?

</td>
<td>Vierfeldertafel
(Vorteile/Nachteile)</td>
<td>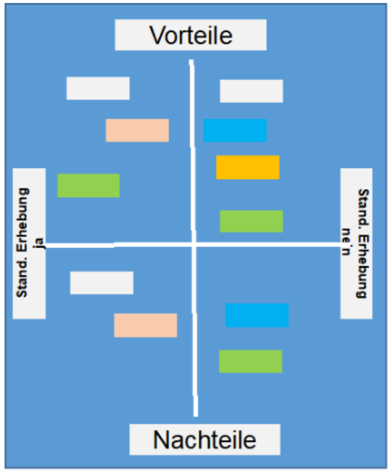
</td>
<td>

Evtl. andere Testpsychometrie
Sonstige Anmerkungen

General: standardisierte Erhebung
PSFS
Hypothesen zum Schmerz
Evtl. andere Testpsychometrie
Sonstige Anmerkungen

</td>
</tr>
<tr>
<td>Arbeitsphase 2
Berufsgruppen-spezifisch/
-übergreifend</td>
<td>
Erfassung des Verständnisses zu den Kriterien und dem Nutzen der fachspezifischen Einschätzung für die Teamsitzung

Welche Kriterien ziehen die Berufsgruppen zur Beurteilung der jeweiligen Einschätzungen heran?
Welcher Zusammenhang besteht zwischen den vier Einschätzungen?
Wie beschreiben die Berufsgruppen jeweils die vier übergeordneten Einschätzungen?
Wie praktikabel ist die Einschätzung in Vorbereitung auf die Teamsitzung?

</td>
<td>Einzelarbeit
(inkl. vorbereiteter Materialien, z.B. DSF, Häufigkeitsver-teilung)

Gruppenarbeit 1 – Kriterien & Verständnis
(Zuordnung & Sortierung Nähe/Distanz)

Gruppenarbeit 2</td>
<td colspan="2">
Fachspezifische Einschätzung für die Teamsitzung:
<table>
<tr><td>Schweregrad Störungsbild</td><td>Bedeutung für Schmerz-problematik</td><td>IMST-Ansatz erforderlich</td><td>Spezifische Behandlung erforderlich</td></tr>
<tr><td>_____</td><td>_____</td><td>_____</td><td>_____</td></tr>
<tr><td>0 = kein
1 = leicht
2 = mittel
3 = schwer</td><td>0 = keine
1 = gering
2 = moderat
3 = hoch</td><td>0 = nein
1 = fraglich
2 = ja</td><td>0 = nein
1 = fraglich
2 = ja</td></tr>
</table>
Fokus Medizin: somatisch
Fokus Psychologie: psycho-sozial
Fokus Physiotherapie funktionell
</td>
</tr>
</table>

<table>
<tr>
<td></td>
<td></td>
<td>Übernahme der Kriterien aus Gruppenarbeit 1 und Einstufung nach:
(1) Wichtigkeit und
(2) Sicherheit

Markt der Ideen
(2-mal)</td>
<td>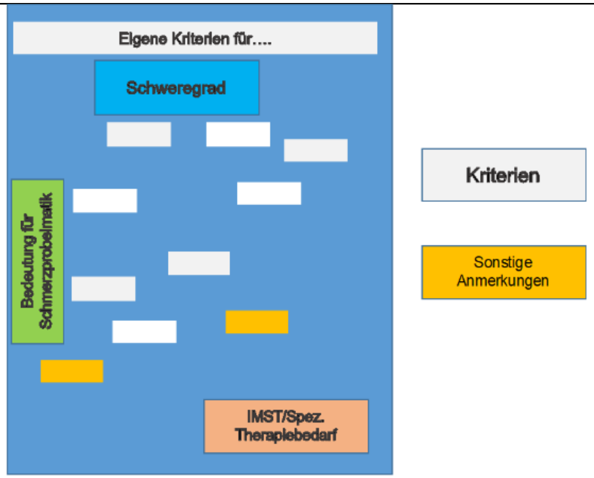

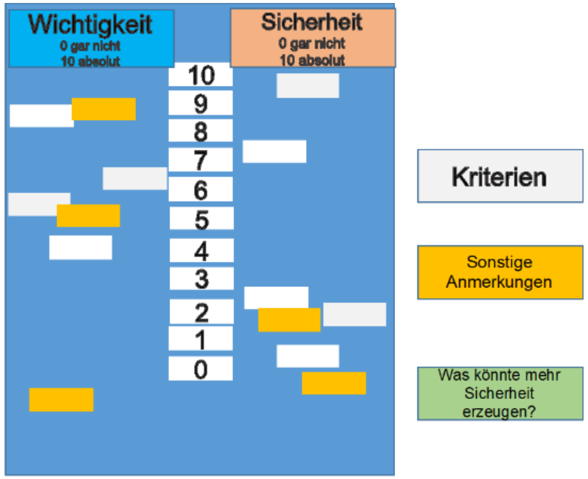
</td>
</tr>
<tr>
<td>Arbeitsphase 3
Interdisziplinär/
Interprofessionell</td>
<td>Therapieempfehlung
• Welche Kriterien werden für die Therapieempfehlungen hinzugezogen?
• Welche Bedeutung hat in diesem Zusammenhang auch die „Einschätzung für die Teamsitzung“?</td>
<td>World Café/ Clustern
(1x60 Minuten, 2x30 Minuten)</td>
<td></td>
</tr>
</table>

	• *Wie kommt es im Team zu einer Therapieempfehlung?*<br>• *Was für Informationen werden von den unterschiedlichen Berufsgruppen benötigt, um eine gemeinsame Empfehlung festzulegen?*		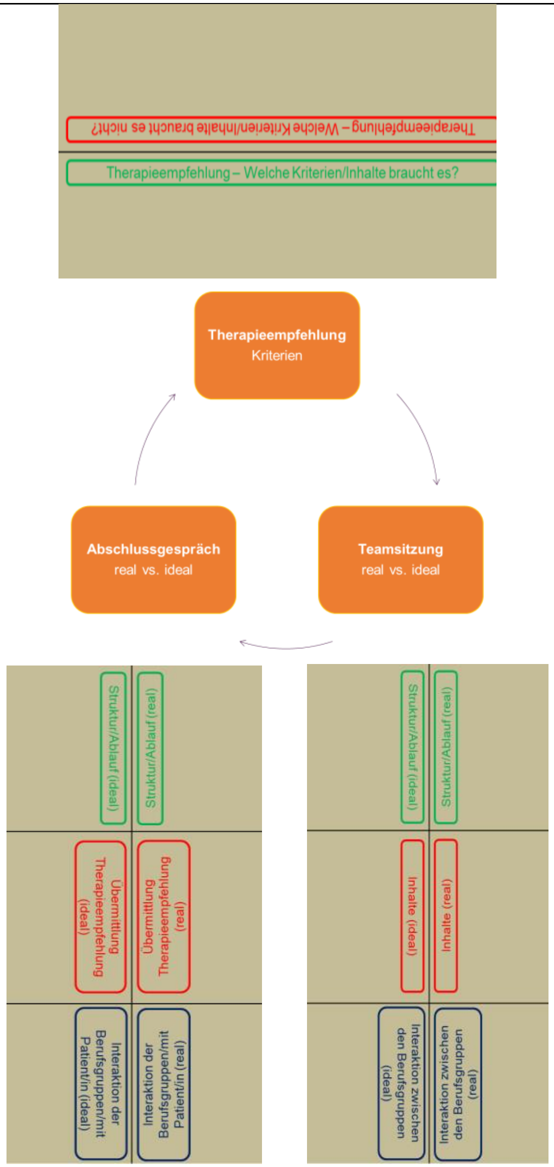
	**Teamsitzung (real vs ideal)**<br>• Wie gestaltet sich der Ablauf einer Teamsitzung?/Wie ist die Teamsitzung strukturiert? (real vs. ideal) → *Struktur und Ablauf einer Teamsitzung*<br>• Welche Inhalte werden in der Teamsitzung besprochen? (real vs. ideal) → *Inhalte einer Teamsitzung*<br>• Wie gestaltet sich die Interaktion der Berufsgruppen während einer Teamsitzung? (real vs. ideal) → *Interaktion innerhalb einer Teamsitzung*		
	**Abschlussgespräch (real vs ideal)**<br>• *Wie gestaltet sich der Ablauf eines Abschlussgesprächs? (real vs. ideal)*<br>• *Wie gestaltet sich die Interaktion mit den Patient:innen und den Berufsgruppen? (real vs. ideal)*<br>• *Wie erfolgt die Übermittlung der Therapieempfehlung? (real vs. ideal)*		
