# Supplementary material for: Feasibility of the interdisciplinary multimodal assessment—The team perspective
Source: Schmerz. 2024 Apr 9;39(6):398–416. [Article in German] doi: 10.1007/s00482-024-00796-z (PMC12627191; doi:10.1007/s00482-024-00796-z)
Supplement: Supplementary file 5 — 5. Gemeinsamkeiten und Unterschiede in der Einschätzung der Kriterien [file 482_2024_796_MOESM5_ESM.pdf]

Online-Zusatzmaterial 5 – Gemeinsamkeiten und Unterschiede in der Einschätzung der Kriterien in Vorbereitung auf die Teamsitzung (ausgewählte Aspekte)

Schweregrad Störungsbild			Bedeutung für die Schmerzproblematik			IMST-Bedarf vs. Spezifische Behandlung		
Medizin	Physiotherapie	Psychologie	Medizin	Physiotherapie	Psychologie	Medizin	Physiotherapie	Psychologie
**Anamnese/Befunderhebung und dessen Interpretationen (eher bei Bedeutung für die Schmerzproblematik) u.a.**								
/Gespräch/ /Klin[ischer] Befund/	/PT-Befund aktiv/passiv/resistiv/	/psychosoziale Belastungsfaktoren/	/Medikamenteneinnahme/ /Korrelation Befund und Schmerz/ /Beeinträchtigung/	/Benennen von schmerzauslösenden Aspekten/ /Lebensqualität: Hobbies, Freizeitgestaltung{	/Psychosoziale Belastung/ /sozialer Kontext: [Waage zwischen] Ressourcen/Gesundheitskompetenz [und] Störungsbild			
**Test- und Assessmentverfahren**								
/MPSS/ /von Korff/	/PSFS/	[fließen unspezifisch mit ein, nicht konkret benannt]	/MPSS/ /von Korff/	/PSFS/	[fließen unspezifisch mit ein, nicht konkret benannt]			
/Vorbefunde/ /Vordiagnostik/			**Patientenbezogene Aspekte**					
			/Arbeitsunfähigkeit/ /häufige Diagnostik bzw. Inanspruchnahme des Gesundheitssystems/ /Leidensdruck/	/Haltung zum Gesundheitsverhalten/Bewegungsverhalten/ /Ressourcen funktionell/körperlich/ /Stellenwert Schmerz für den Pat./ /Leidensdruck/	/Ausmaß Introspektions- und Reflexionsfähigkeit/ /Ressourcen: innere, äußere; „wie ist der Zugang dazu“/	/Zugänglichkeit für multimodalen Ansatz/ /Aufgeschlossenheit für Therapie/ /Vorliegen multimodaler Problematik/ /Motivation/ /Beeinträchtigung im Alltag/ /subjektiver Leidensdruck/	/Stellenwert Schmerz für den Pat./	/Passung Person zu IMST Inhalten/ /Ausmaß an ressourcen für den Transfer/
						**Chronifizierungsprozess**		
					/gute Prognose [dazu sortiert:] zeitnahe Lösung, Abgrenzbarkeit der psychosoz[ialen] Beeinträchtigung/	/psychosoziale Faktoren deutlich/	/Dauer Chronifizierungsprozess/ /Dauer der Symptomatik bzw.	/Gefahr der Chronifizierung/

/schlechte Prognose [dazu sortiert:] wenig/keine Lösungsoption; breites/komplexes Problem- bzw. Belastungsfeld		wiederkehrend[e Schmerzen]/	
		**Frage nach IMST-Ansatz**	
	/Diskrepanz Störungsbild und subj[ektiver] Eindruck/<br>/Hinweis auf affektives Schmerzerleben/<br>/schlechtes Coping bzw. umgang mit Schmerz dysfunktional/<br>/Schulung/Edukation [im Sinne Bedarf von]…/	/spez. Vorbehandlungen/<br>/Biopsychosoziales Modell/<br>/Inkonsistenzen im Einzelbefund/	/Informierung über biopsychosoziales Schmerzmodell erscheint wichtig/<br>/verschiedene Ebenen relevant oder „nur“ 1?/
		**Spezifischer Therapiebedarf**	
	/muss ein „spezielles Problem“ gelöst werden/	/spez. Behandlung bei funktioneller Problematik/	/Ablehnung psychische Ansätze [meint ANDERE Angebote als Psycho]/<br>/psych[ische] Erkrankung im Vordergrund/

Legende:

Gemeinsamkeiten Unterschiede

Medizin Physiotherapie Psychologie
