# Supplementary material for: Feasibility of the interdisciplinary multimodal assessment—The team perspective
Source: Schmerz. 2024 Apr 9;39(6):398–416. [Article in German] doi: 10.1007/s00482-024-00796-z (PMC12627191; doi:10.1007/s00482-024-00796-z)
Supplement: Supplementary file 6 — 6. Dokumentationsunterlagen PAIN2020 [file 482_2024_796_MOESM6_ESM.pdf]

PAIN2020 B4

Fragebogen für Patienten mit länger anhaltenden Schmerzen

Deutsche Schmerzgesellschaft e.V.

Datum: ___________ **Name Patient/in:** ____________________________

Bitte füllen Sie diesen Fragebogen aus und beantworten jede der Fragen, indem Sie entweder „ja" oder „nein" ankreuzen. Die Fragen helfen uns bei der Einschätzung Ihres Krankheitsbildes.

1. Ich befürchte, dass ich eine ernste, bedrohliche Erkrankung habe.

☐ ja ☐ nein

2. Wegen der Schmerzen habe ich meine körperlichen Aktivitäten sehr stark reduziert.

☐ ja ☐ nein

3. Ich leide neben den Schmerzen auch noch an anderen körperlichen Symptomen (wie z.B. Magen- und Darmverstimmungen, Schwächegefühle, Schwindel oder Hitzewallungen, Luftnot, Herzrasen, Enge in der Brust, Schwitzen, Kälteschauer, Übelkeit, Kribbeln, Taubheitsgefühle, Schlafstörungen etc.).

☐ ja ☐ nein

4. Ich war **in den letzten beiden Wochen** meistens traurig oder niedergeschlagen.

☐ ja ☐ nein

5. Ich habe mich **in den letzten beiden Wochen** meistens angespannt oder besorgt gefühlt.

☐ ja ☐ nein

6. Ich fühle mich in Bezug auf meine Schmerzen sehr stark verunsichert und weiß überhaupt nicht mehr, was richtig oder falsch ist.

☐ ja ☐ nein

7. Seit einiger Zeit gibt es bei mir Probleme in der Partnerschaft und/oder in der Familie und/oder dem engeren Freundeskreis.

☐ ja ☐ nein

8. Falls Sie in einem Arbeitsverhältnis stehen: Es gibt momentan erhebliche Belastungen/Schwierigkeiten im Zusammenhang mit meinem Arbeitsplatz.

☐ ja ☐ nein ○ Ich bin derzeit nicht berufstätig

9. Auch bei starken Schmerzen neige ich dazu, alles durchzuziehen und meine Aufgaben abzuschließen.

☐ ja ☐ nein

10. Ich habe in der letzten Zeit häufiger an schreckliche Ereignisse von **früher** denken müssen oder Träume darüber gehabt (wie z.B. Unfall oder Gewalterfahrung).

☐ ja ☐ nein

PAIN2020 **B5**

Deutsche Schmerzgesellschaft e.V.

Aufnahmebogen Zentrum

Patient/in: ______________________________ **Datum:** ______________________

Zuweisung durch:	☐ Arzt/Ärztin	☐ BARMER	☐ Patient/in	☐ andere Kasse: ____________________

**Einschlusskriterien**			
1	○ Alter: mind. 18 Jahre<br>• gesetzlich krankenversichert		☐
2	• Schmerzen<br>– seit mind. 6 Wochen und/oder<br>– in kürzeren Phasen innerhalb der letzten beiden Jahre mehrfach auftretend		☐
3	• Schmerz-bedingte Einschränkungen im Lebensvollzug<br>– Schmerz-bedingte AU seit mind. 4 Wochen oder kumulierte AU von mind. 6 Wochen in den vergangenen 12 Monaten und/oder<br>– Schmerz-bedingte deutliche Beeinträchtigung in Familie/Freizeit/Arbeit		☐
4	• Risikofaktoren für Chronifizierung erkennbar (mind. 1 Punkt)<br>– bzgl. Lokalisation: sich ausbreitende Schmerzen<br>– ausgeprägtes (verbales/non-verbales) Schmerzverhalten<br>– ungünstige Schmerzverarbeitung (Fokussierung, Ängste, …)<br>– ausgeprägtes Schon- und Vermeidungsverhalten<br>– Hinweise auf Stressbelastung in Familie/Partnerschaft/soz. Umfeld/Beruf<br>– depressive Symptome im Erleben und/oder Verhalten<br>– Befindlichkeit geprägt durch Frustration/Ärger<br>– ungünstige Verhaltensweisen (Überforderung, „Durchhalten“)<br>– Hinweise auf Somatisierung (z.B. vielfältige, „bunte“ Symptomatik)<br>– hohes Inanspruchnahmeverhalten im Versorgungssystem<br>– Wunsch nach fortgesetzter Krankschreibung<br>– Wunsch nach fortgesetzter Diagnostik	☐	Wenn mind. 1 Kreuz in li. Spalte<br>☐
	• Mindestens 3 positive Angaben im Patienten-Fragebogen	☐	
	• Ungeklärte Fragen<br>(über Diagnose, Pathologie, über erforderlichen Krankenhausaufenthalt oder über Erfordernis operativen Vorgehens, Reha-Bedarf)	☐	
5	Erreichbarkeit: Behandlungszentrum ist für Pat. erreichbar		☐
**Ausschlusskriterien**			
6	• Andere, akut wirksame schwerwiegende Erkrankungen, die z.B. eine aktivierende Behandlung verhindern	☐	Wenn kein Punkt erfüllt<br>☐
	• Eindeutige „Red Flags“	☐	
	• Manifeste chron. Schmerzerkrankung<br>(z.B. AU wg. Schmerzen länger als 6 Monate, Schmerz-relevante Diagnose über mehr als 4 Quartale, vorhergehende Therapie mit starken Opioiden über 3 Monate Dauer, vorhergehende IMST in letzten beiden Jahren; FMS)	☐	
	• Lfd. Diagnostik und/oder Therapie bzgl. schmerzspezif. Pathologie	☐	
	• Schwere und aktive psychiatrische Störung<br>(Persönlichkeitsstörung, schwere Depression oder Angsterkrankung, Hinweise auf Suizidalität)	☐	
	• Laufendes Renten- oder Reha-Verfahren (beantragt)	☐	
	• Sprachliche und / oder kognitive Einschränkungen, die eine Durchführung des IMA/der SRV unmöglich erscheinen lassen	☐	

Zuweisung in Studie, falls alle Punkte in rechter Spalte zutreffend ↑

Studienteilnahme:	☐ Einschluss	☐ Nicht-TN trotz E-Möglichkeit	☐ Ausschluss

B13
Zusatz zur Routine mit DSF: PCS (Papierversion)

Patienten-ID:_____________________________ Datum beim Ausfüllen: |__|__| |__|__| |__|__|__|__|
Tag Monat Jahr

Schmerz-Bewertungs-Skala (PCS)

Die folgenden dreizehn Sätze beschreiben verschiedene Gedanken und Gefühle, die bei Schmerzen auftreten können. Bitte markieren Sie auf der folgenden Skala eine Zahl bei jeder Frage, wie stark diese Gedanken und Gefühle auf Sie zutreffen, wenn Sie Schmerzen haben.

Bewertung:
0 = trifft überhaupt nicht zu, 1 = trifft eher nicht zu, 2 = teils-teils, 3 = trifft eher zu, 4 = trifft immer zu

1. Ich mache mir ständig Sorgen, ob die Schmerzen wohl jemals wieder aufhören werden.
   |__0__|__1__|__2__|__3__|__4__|H

2. Ich denke, ich kann nicht mehr.
   |__0__|__1__|__2__|__3__|__4__|H

3. Der Zustand ist schrecklich und ich denke, dass es nie mehr besser wird.
   |__0__|__1__|__2__|__3__|__4__|H

4. Der Zustand ist furchtbar und droht mich zu überwältigen.
   |__0__|__1__|__2__|__3__|__4__|H

5. Ich habe das Gefühl, ich halte es nicht mehr aus.
   |__0__|__1__|__2__|__3__|__4__|H

6. Ich bekomme Angst, dass die Schmerzen noch stärker werden.
   |__0__|__1__|__2__|__3__|__4__|M

7. Ich denke ständig an andere Situationen, in denen ich Schmerzen hatte.
   |__0__|__1__|__2__|__3__|__4__|M

8. Ich wünsche mir verzweifelt, dass die Schmerzen weggehen.
   |__0__|__1__|__2__|__3__|__4__|R

9. Ich kann nicht aufhören, an die Schmerzen zu denken.
   |__0__|__1__|__2__|__3__|__4__|R

10. Ich denke ständig daran, wie sehr es schmerzt.
    |__0__|__1__|__2__|__3__|__4__|R

11. Ich denke ständig daran, wie sehr ich mir ein Ende der Schmerzen herbeiwünsche.
    |__0__|__1__|__2__|__3__|__4__|R

12. Es gibt nichts, was ich tun kann, um die Schmerzen zu lindern.
    |__0__|__1__|__2__|__3__|__4__|R

13. Ich mache mir Sorgen, dass die Schmerzen auf etwas Schlimmes hindeuten.
    |__0__|__1__|__2__|__3__|__4__|M

Diese Zeile bitte nicht ausfüllen: H: _______ M: _______ R: _______ G: _______

Schmerz-Bewertungs-Skala Pain Catastrophizing Score © Meyer et al. 2008

C4

IMA
ärztliche Dokumentation

IMA-Termin Datum: __________________ Pat-ID: __________________

Befunddokumentation

Kriterium	Befund/ Handlungsbedarf	Beurteilung	Kommentar
Morphologische, strukturelle Schädigung		spezif. fachmediz. Therapie indiziert/erforderlich?<br>Ja ☐ Fraglich ☐ Nein ☐	
Weitere Diagnostik vor IMST notwendig		Ja ☐ Nein ☐	
Medikation		Gruppenfähigkeit für IMST<br>Ja ☐ Fraglich ☐ Nein ☐	
Allgemeine Dekonditionierung		Gruppenfähigkeit für IMST<br>Ja ☐ Fraglich ☐ Nein ☐	
Kardiovaskuläre Belastbarkeit		Kontraindikationen für IMST<br>Ja ☐ Fraglich ☐ Nein ☐	
Weitere relevante Komorbiditäten		Kontraindikationen für IMST<br>Ja ☐ Fraglich ☐ Nein ☐	

Ziele Patient/in:

____

____

____

Ziele aus ärztlicher Sicht:

____

____

____

C4

IMA
ärztliche Dokumentation

Deutsche Schmerzgesellschaft e.V.

Pat-ID: ________________

Einschätzung für Teamsitzung

	Schweregrad Störungsbild (somatisch)	Bedeutung f. Schmerz-problematik	Sicherheit der Ein-schätzung	IMST-Ansatz erforderlich	Spezifische Behandlung erforderlich	Gruppen-fähigkeit für IMST	Kontra-indikation für IMST
**Ärztlich**	________	________	________	________	________	________	________
	0 = kein<br>1 = leicht<br>2 = mittel<br>3 = schwer	0 = keine<br>1 = gering<br>2 = moderat<br>3 = hoch	1 = gering<br>2 = mittel<br>3 = hoch	0 = nein<br>1 = fraglich<br>2 = ja	0 = nein<br>1 = fraglich<br>2 = ja	0 = nein<br>1 = fraglich<br>2 = ja	0 = nein<br>1 = fraglich<br>2 = ja

Bitte bewerten Sie im Anschluss an das IMA folgende Aussagen und notieren Sie gerne Ihre Anmerkungen im Freitext:

**Vom Arzt auszufüllen**	stimme voll und ganz zu	stimme eher zu	weder noch	stimme eher nicht zu	stimme überhaupt nicht zu
Es lagen relevante Vorbefunde in ausreichendem Maß vor.	☐	☐	☐	☐	☐
Es war ausreichend Zeit vorhanden, die vorliegenden Befunde zu sichten.	☐	☐	☐	☐	☐
Die Zeit für die Umsetzung des ärztlichen IMA war ausreichend.	☐	☐	☐	☐	☐
Freitext:					

C5

Ergänzende Arztangaben (KEDOQ-Schmerz)

IMA

Deutsche Schmerzgesellschaft e.V.

Assessment-Termin Datum: ____________ Pat-ID: __________

B3. Setting der eingeleiteten bzw. veranlassten Behandlung

O ambulant	O teilstationär	O stationär	O keine Therapie
			O geplanter einmaliger Kontakt<br>O ausschließlich Diagnostik<br>O Gutachten

B4. Ergänzende Informationen zum Schmerz

Hauptschmerzlokalisation (mindestens 1 Oberbegriff)

Kopf	Gesicht	Nacken/ Hals	Schulter/ Arm
O Gesamter Kopf bzw. Kopfhälfte<br>O occipital<br>O parietal<br>O Vertex<br>O Stirn	O Gesamtes Gesicht bzw. Gesichtshälfte<br>O Oberkiefer<br>O Unterkiefer	O Nacken<br>O Hals	O Schulter<br>O Gesamter Arm<br>O Oberarm<br>O Unterarm<br>O Ellenbogen<br>O Handgelenk<br>O Hand<br>O Finger, Fingergelenke

Thorax	O Rücken, BWS	O Kreuz, LWS	Abdomen
O Gesamter Thorax<br>O Thorax lateral<br>O Thorax ventral			O Gesamtes Abdomen<br>O Oberbauch<br>O Unterbauch<br>O Flanke

Becken	O Genital-/ Perianalregion	Bein	O Wide spread pain
O Gesamtes Becken<br>O Leiste<br>O Hüftbereich<br>O Kreuzbein<br>O Steißbein		O Gesamtes Bein<br>O Oberschenkel<br>O Unterschenkel<br>O Knie<br>O Sprunggelenk<br>O Fuß<br>O Zehen, Zehengelenke	
O Polyarthralgien	O Hemi-/ Panalgesie	O Gesamter oberer Quadrant	O Gesamter unterer Quadrant

O Sonstige Schmerzverteilung: ____

Ergänzende Arztangaben (KEDOQ-Schmerz)

IMA

Mainz Pain Staging System (MPSS)

Pat-ID: ________________

Achse 1: Zeitliche Aspekte	Wert		Achsensumme	Achsenstadium
**Auftretenshäufigkeit**: einmal täglich o. seltener	1			
mehrmals täglich	2			3=I
dauernd	3			4-6=II
**Dauer**: bis zu mehreren Stunden	1			7-9=III
mehrere Tage	2		Σ	
länger als eine Woche oder dauernd	3			
**Intensitätswechsel**: häufig	1			
gelegentlich	2			
nie	3			
**Achse 2: Räumliche Aspekte**				
monolokulär	1			
bilokulär	2			
multilokulär oder Panalgesie	3			
**Achse 3: Medikamenteneinnahmeverhalten**				
**Medikamenteneinnahme**: unregelmäßiger Gebrauch von max.2 peripheren Analgetika	1			
max. 3 periphere Analgetika, höchstens 2 regelmäßig	2			2=I
regelmäßig mehr als 2 periphere Analgetika oder zentralwirkende Analgetika	3			3-4=II<br>5-6=III
**Anzahl der Entzugsbehandlungen:** keine	1		Σ	
eine	2			
mehr als eine	3			
**Achse 4: Patientenkarriere**				
**Wechsel des persönlichen Arztes:** kein Wechsel	1			4=I
max. 3 Wechsel	2			5-8=II
mehr als 3 Wechsel	3			9-12=III
**Schmerzbedingte Krankenhausaufenthalte**: bis 1	1		Σ	
2 bis 3	2			
mehr als 3	3			
**Schmerzbedingte Operationen**: bis 1	1			
2 bis 3	2			
mehr als 3	3			Summe der Achsenstadien
**Schmerzbedingte Rehabilitationsmaßnahmen**: keine	1			I = 4 - 6
bis 2	2			II= 7 - 8
mehr als 2	3			III=9 - 12

Hauptschmerzdiagnose (ICD-Code, CRPS I und II ergänzen):______________________________

Weitere somatische Diagnosen (keine oder ICD-Code):______________________________

Psychologisch/Psychiatrische Diagnosen (keine, oder ICD-Code): ______________________________

B5. Medikamentöse Behandlung vor Therapiebeginn

O **keine Medikamente**

Oder Medikamente aus folgenden Gruppen…

O NSAR, Coxibe	O Andere Nichtopioide	O Mischpräparat	O Triptane
O WHO II	O WHO III	O Antidepressiva	O Antikonvulsiva
O Muskelrelaxantien	O Benzodiazepine	O Antiemetika	O Laxantien

Sonstige schmerzbezogene Substanzen: ______________________________

C7

IMA
Patientenspezifische Funktionsskala (PSFS)

Deutsche Schmerzgesellschaft e.V.

IMA-Termin Datum: ______________ **Pat-ID: ____________________**

Dieser Fragebogen soll dazu verwendet werden, individuelle Aktivitätseinschränkungen im Alltag zu quantifizieren.

Procedere: Am Ende der Anamnese und vor der körperlichen Untersuchung auszufüllen.

Leitfrage an Patienten in der physiotherapeutischen Untersuchung:
„Ich möchte Sie bitten, drei wichtige Aktivitäten zu nennen, welche Sie aufgrund Ihres Schmerzproblems nicht ausführen können oder bei welchen Sie Schwierigkeiten bei der Ausführung haben."

Anschließend dem Patienten die 10-stufige Skala zeigen, erklären und um Einschätzung bitten.

Bewertungsschema für die Ausführung patientenspezifischer Aktivitäten

0	1	2	3	4	5	6	7	8	9	10
Unfähig, die Aktivität auszuführen										Kann die Aktivität auf dem gleichen Niveau wie vor der Verletzung oder dem Problem ausführen

Individuelle Aktivität	Punktzahl 0 - 10
1.	
2.	
3.	

Stratford, P, Gil, C, Westaway M, & Binkley J (1995) Assessing disability and change on individual patients: a report of a patient specific measure. Physiotherapy Canada 47, 258-263. © P. Stratford 1995, reprinted with permission.
© Kulturell adaptiert ins Deutsche: Heldmann 2012, HAWK Hildesheim. (modifiziert)

C8

IMA
physiotherapeutische Dokumentation

Deutsche Schmerzgesellschaft e.V.

IMA-Termin Datum: ____________________ Pat-ID: ______________________

Kriterium	Befund	Beurteilung	Kommentar
Funktionsstörung	Unilokulär: ___________<br>Bi-/Multilokulär: ______ Orte	Spezifische physio-therapeutische Behandlung, Intervention erforderlich<br>Ja ☐ Fraglich ☐ Nein ☐	
	Verkettung: Ja ☐ Nein ☐		
	komplexeres Bild: Ja ☐ Nein ☐		
Spezifische Einschränkungen		Gruppenfähigkeit für IMST<br>Ja ☐ Fraglich ☐ Nein ☐	
Gehstrecke		Gruppenfähigkeit für IMST<br>Ja ☐ Fraglich ☐ Nein ☐	
Transfer		Gruppenfähigkeit für IMST<br>Ja ☐ Fraglich ☐ Nein ☐	
Kardiovaskuläre Belastbarkeit		Gruppenfähigkeit für IMST<br>Ja ☐ Fraglich ☐ Nein ☐	

C8

IMA
physiotherapeutische Dokumentation

Deutsche Schmerzgesellschaft e.V.

Pat-ID: ____________________

Allg. Dekonditionierung		Gruppenfähigkeit für IMST<br>Ja ☐ Fraglich ☐ Nein ☐	
Alltagsbelastbarkeit Ergebnis im **PSFS**: Summe der Punktzahl der jeweiligen Aktivitäten	1. ________________/___<br>2. ________________/ ___<br>3. ________________/ ___<br>Mittelwert NRS aus 1.-3. ___		
Sinne (sehen, hören, Gleichgewicht)			
Vegetativer Status			
Hypermobilität			
Hypomobilität			

Ziele Patient/in:

____

____

____

____

Ziele aus physiotherapeutischer Sicht:

____

____

____

____

C8

IMA
physiotherapeutische Dokumentation

Pat-ID: ____________________

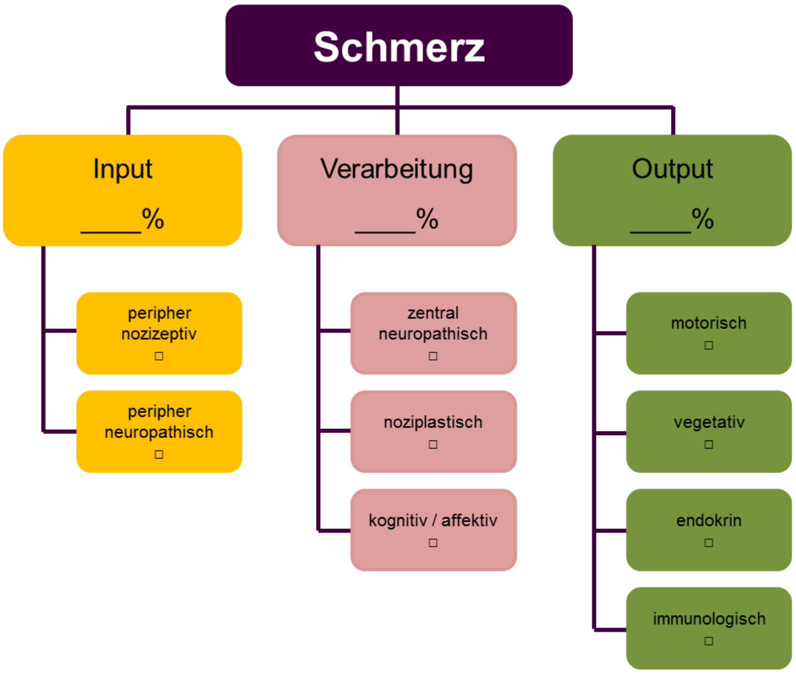

Bitte zeichnen Sie die Verteilung von

- Input (I)
- Verarbeitung (V)
- Output (O)

Einschätzung für Teamsitzung

	Schweregrad Störungsbild (funktionell)	Bedeutung f. Schmerz-problematik	Sicherheit der Ein-schätzung	IMST-Ansatz erforderlich	Spezifische Behandlung erforderlich	Gruppen-fähigkeit für IMST	Kontra-indikation für IMST
**Physio-thera-peutisch**	________	________	________	________	________	________	________
	0 = kein<br>1 = leicht<br>2 = mittel<br>3 = schwer	0 = keine<br>1 = gering<br>2 = moderat<br>3 = hoch	1 = gering<br>2 = mittel<br>3 = hoch	0 = nein<br>1 = fraglich<br>2 = ja	0 = nein<br>1 = fraglich<br>2 = ja	0 = nein<br>1 = fraglich<br>2 = ja	0 = nein<br>1 = fraglich<br>2 = ja

Bitte bewerten Sie im Anschluss an das IMA folgende Aussagen und notieren Sie gerne Ihre Anmerkungen im Freitext:

**Vom Physiotherapeuten auszufüllen**	stimme voll und ganz zu	stimme eher zu	weder noch	stimme eher nicht zu	stimme überhaupt nicht zu
Es lagen relevante Vorbefunde in ausreichendem Maß vor.	☐	☐	☐	☐	☐
Es war ausreichend Zeit vorhanden, die vorliegenden Befunde zu sichten.	☐	☐	☐	☐	☐
Die Zeit für die Umsetzung des physiotherapeutischen IMA war ausreichend.	☐	☐	☐	☐	☐
Freitext:					

C9

IMA
Ergänzender Fragebogen
(PHQ-15 und SKID-Befindlichkeit)

Deutsche Schmerzgesellschaft e.V.

IMA-Termin Datum: ____________________ Pat-ID: ________________

Schmerz-Beschwerden (PHQ-15)

Sie finden auf diesem Blatt eine Liste von körperlichen Beschwerden, die man manchmal hat. Bitte lesen Sie jede Frage sorgfältig durch und entscheiden Sie, wie sehr Sie **in den letzten Tagen** durch diese Beschwerden gestört oder bedrängt worden sind.
Bitte beantworten Sie jede Frage.

Wie sehr litten Sie in den letzten Tagen unter …?

		nicht beeinträchtigt	wenig beeinträchtigt	stark beeinträchtigt
1.	Bauchschmerzen……………………………………	[0]	[1]	[2]
2.	Rückenschmerzen……………………………….	[0]	[1]	[2]
3.	Schmerzen in Armen, Beinen, Gelenken (Knie, Hüfte, etc.)………………………………...	[0]	[1]	[2]
4.	Menstruationsbeschwerden oder andere Probleme Ohnmachts- oder Schwindelgefühlen..	[0]	[1]	[2]
5.	Schmerzen oder Probleme beim Geschlechts-verkehr…………………………………………….	[0]	[1]	[2]
6.	Kopfschmerzen…………………………………..	[0]	[1]	[2]
7.	Schmerzen im Brustbereich…………………….	[0]	[1]	[2]
8.	Schwindel………………………………………..	[0]	[1]	[2]
9.	Ohnmachtsanfälle………………………………..	[0]	[1]	[2]
10.	Herzklopfen oder Herzrasen…………………….	[0]	[1]	[2]
11.	Kurzatmigkeit……………………………………..	[0]	[1]	[2]
12.	Verstopfung, nervöser Darm oder Durchfall……..	[0]	[1]	[2]
13.	Übelkeit, Blähungen oder Verdauungsbeschwerden………………………….	[0]	[1]	[2]

		überhaupt nicht	an einzelnen Tagen	an mehr als der Hälfte aller Tage
14.	Schwierigkeiten, ein- oder durchzuschlafen, oder vermehrter Schlaf……………………………..	[0]	[1]	[2]
15.	Müdigkeit, oder das Gefühl keine Energie zu haben………………………………………………...	[0]	[1]	[2]

C9

IMA
Ergänzender Fragebogen
(PHQ-15 und SKID-Befindlichkeit)

SKID-Befindlichkeit

Pat-ID: ________________

Wir möchten Ihnen noch einige wichtige Fragen zu Ihrer allgemeinen Befindlichkeit stellen. Bitte beantworten Sie die Fragen möglichst mit ja oder nein. Sollten Sie unsicher sein, können Sie auch mit „unsicher“ antworten.

		0	1	2
1.	Haben Sie in letzter Zeit fünf oder mehr Gläser Alkohol (Bier, Wein oder Likör) auf einmal getrunken?	nein	unsicher	ja
2.	Nehmen Sie Drogen?	nein	unsicher	ja
3.	Fühlen Sie sich von einem ärztlich verschriebenen Medikament abhängig oder nehmen Sie mehr davon ein als Ihnen verschrieben wurde?	nein	unsicher	ja
4.	Leiden Sie unter Angstanfällen, bei denen Sie ganz plötzlich in panischen Schrecken geraten oder starke Angst haben?	nein	unsicher	ja
5.	Haben Sie Angst, alleine das Haus zu verlassen, sich in einer Menschenmenge zu befinden, in einer Schlange anzustehen oder mit dem Zug oder Bus zu fahren?	nein	unsicher	ja
6.	Haben Sie Angst davor oder ist es Ihnen unangenehm, in Gegenwart anderer Menschen zu sprechen, zu essen oder zu schreiben?	nein	unsicher	ja
7.	Gibt es andere Dinge, vor denen Sie besonders Angst haben, wie z.B. in einem Flugzeug zu sitzen, Blut zu sehen, sich in geschlossenen Räumen aufzuhalten, vor bestimmten Tieren oder vor Höhe?	nein	unsicher	ja
8.	Leiden Sie unter Gedanken, die unsinnig sind und immer wieder kommen, auch wenn Sie es gar nicht wollen?	nein	unsicher	ja
9.	Kommt es vor, dass Sie bestimmte Dinge immer und immer wieder tun müssen, wie z.B. sich immer wieder die Hände zu waschen oder etwas mehrmals zu kontrollieren, um sicherzugehen, dass Sie es richtig gemacht haben?	nein	unsicher	ja
10.	Waren Sie in den letzten sechs Monaten besonders nervös oder ängstlich?	nein	unsicher	ja
11.	Kam es in letzter Zeit vor, dass andere Menschen sagten, Sie seien zu dünn?	nein	unsicher	ja
12.	Haben Sie Essanfälle, bei denen Sie das Gefühl haben, Ihr Essverhalten nicht mehr kontrollieren zu können?	nein	unsicher	ja
13.	Leiden Sie seit mehreren Monaten unter körperlichen Beschwerden, für die Ihr Arzt keine eindeutige Erklärung finden konnte?	nein	unsicher	ja
14.	Haben Sie jemals ein ungewöhnlich schreckliches oder bedrohliches Ereignis oder eine ungewöhnlich schreckliche oder bedrohliche Situation erlebt, unter deren Nachwirkungen Sie heute noch leiden?	nein	unsicher	ja
15.	Fühlen Sie sich mit der Bewältigung eines einschneidenden Ereignisses in Ihrem Leben so stark belastet, dass dadurch Ihr Befinden spürbar beeinträchtigt ist?	nein	unsicher	ja

DIBpS1-Projektteam:
Jahed, J., Vogel, B., Baumeister, H., Härter, M., Barth, J. & Bengel, J.
Diagnostik, Indikation und Behandlung von psychischen Störungen in der medizinischen Rehabilitation

C11

IMA
psychologische Dokumentation inkl. MASK-P

Deutsche Schmerzgesellschaft e.V.

IMA-Termin Datum: ________________ Pat-ID: _________________

Dokumentation Testbefunde

Schmerzintensität:	momentan ____ durchschnittlich ____ maximal ____
Beeinträchtigung:	Alltag ____ Freizeit ____ Arbeit ____
von Korff:	_____
DASS:	Depressivität ______ Angst ________ Stressbelastung ______
VR-12:	Physische Gesundheit, PCS ______________ Mentale Gesundheit, MCS ____________
Weitere:	FW7: ______ SBL-A: ______
PCS:	Hilflosigkeit ____ Magnification ____ Rumination ____ ∑ ____
PHQ-15:	______
SKID-Befindlichkeit:	Auffällig?<br>Ja ☐ Nein ☐<br>Wenn ja, auffällige Bereiche ankreuzen:<br>☐ 1 ☐ 2 ☐ 3 ☐ 4 ☐ 5 ☐ 6 ☐ 7 ☐ 8 ☐ 9 ☐10 ☐ 11 ☐ 12 ☐ 13 ☐ 14 ☐ 15
MASK-P:	1 (__)→__ __ __, 5 (__)→__ __ __,<br>2 (__)→__ __ __, 6 (__)→(__) __ (__) __ (__) __,<br>3 (__)→__ __ __, 7 (__)→__ __ __,<br>4 (__)→__ __ __, 8 (__)→__ __ __.

Ziele Patient/in:

____

____

Ziele aus psychologischer Sicht:

____

____

C11

IMA
psychologische Dokumentation inkl. MASK-P

Pat-ID: ________________

Einschätzung für Teamsitzung

	Schweregrad Störungsbild (psychosozial)	Bedeutung f. Schmerz-problematik	Sicherheit der Ein-schätzung	IMST-Ansatz erforderlich	Spezifische Behandlung erforderlich	Gruppen-fähigkeit für IMST	Kontra-indikation für IMST
**psycho-logisch**	________	________	________	________	________	________	________
	0 = kein<br>1 = leicht<br>2 = mittel<br>3 = schwer	0 = keine<br>1 = gering<br>2 = moderat<br>3 = hoch	1 = gering<br>2 = mittel<br>3 = hoch	0 = nein<br>1 = fraglich<br>2 = ja	0 = nein<br>1 = fraglich<br>2 = ja	0 = nein<br>1 = fraglich<br>2 = ja	0 = nein<br>1 = fraglich<br>2 = ja

Achsenzusatzkodierung: *Für alle Achsen wird jeweils eines der folgenden Merkmale vergeben:*

1 Achse wurde nicht untersucht
2 keine Auffälligkeiten identifizierbar
3 Patient/in sieht Auffälligkeiten nicht
4 Patient/in sieht Auffälligkeiten

Für jede Achse können maximal 3 Auffälligkeiten vergeben werden.

**Achse 1: Motorisch-verhaltensmäßige Schmerzverarbeitung**		
Achsen-Zusatzkodierung:		*kodiere 1, 2, 3 oder 4*
Bis zu 3 Auffälligkeiten markieren durch ankreuzen<br><br>auf Doku-Beleg übertragen	1	Ausgeprägt non-verbales Schmerzverhalten
	2	Ausgeprägt verbales Schmerzverhalten
	3	Diskrepanz zwischen verbalem und non-verbalem Schmerzverhalten
	4	Defizite im Bitten um soziale Unterstützung
	5	Ausgeprägte Vermeidung körperlicher Aktivitäten
	6	Ausgeprägte Vermeidung sozialer Aktivitäten
	7	Ausgeprägtes Durchhalteverhalten
	8	Nichteinhaltung erforderlichen Gesundheitsverhaltens

**Achse 2: Emotionale Schmerzverarbeitung**		
Achsen-Zusatzkodierung:		*kodiere 1, 2, 3 oder 4*
Bis zu 3 Auffälligkeiten markieren durch ankreuzen<br><br>auf Doku-Beleg übertragen	1	Traurig-niedergeschlagene Stimmung
	2	Ärgerlich-gereizte Stimmung
	3	Ängstliche Stimmung
	4	Leichte innere Erregbarkeit
	5	Eingeschränktes emotionales Erleben
	6	Mangelnder Emotionsausdruck
	7	Übertrieben positiver Emotionsausdruck

C11

PAIN2020

IMA
psychologische Dokumentation inkl. MASK-P

Deutsche Schmerzgesellschaft e.V.

Pat-ID: ________________

**Achse 3: Kognitive Schmerzverarbeitung**		
Achsen-Zusatzkodierung:		*kodiere 1, 2, 3 oder 4*
Bis zu 3 Auffälligkeiten markieren durch ankreuzen<br><br>auf Doku-Beleg übertragen	1	Hilflosigkeit / Katastrophisieren
	2	Resignation / Hoffnungslosigkeit
	3	Suizidgedanken
	4	Mangelhafte Wahrnehmung körperlicher Vorgänge
	5	Ausgeprägte Bagatellisierung körperlicher Vorgänge
	6	Ausgeprägte Selbstaufmerksamkeit für körperliche Vorgänge
	7	Ausgeprägter Durchhalteappell

**Achse 4: Krankheitsbezogene Metakognitionen**		
Achsen-Zusatzkodierung:		*kodiere 1, 2, 3 oder 4*
Bis zu 3 Auffälligkeiten markieren durch ankreuzen<br><br>auf Doku-Beleg übertragen	1	Ausgeprägtes somatisches Krankheitsmodell
	2	Ausgeprägte stabile Ursachenattribution
	3	Ausgeprägte externale Kontrollattribution
	4	Ausgeprägte internale Kontrollattribution
	5	Schuldzuschreibungen
	6	Ausgeprägte Fear-Avoidance-Beliefs
	7	Ausgeprägte Endurance-Beliefs

**Achse 5: Aktuelle Stressoren**		
Achsen-Zusatzkodierung:		*kodiere 1, 2, 3 oder 4*
Bis zu 3 Auffälligkeiten markieren durch ankreuzen<br><br>auf Doku-Beleg übertragen	1	Physikalische Belastungen am Arbeitsplatz
	2	Psychosoziale Belastungen am Arbeitsplatz
	3	Unklarer beruflicher Status
	4	Erhebliche finanzielle Belastungen
	5	Probleme im Familien- und/oder Freundeskreis
	6	Ehe- / Partnerschaftsprobleme
	7	Belastungen durch zusätzliche gesundheitliche Probleme
	8	Krisenhafte Ereignisse
	9	Belastungen im Freizeitbereich

C11

IMA
psychologische Dokumentation
inkl. MASK-P

Deutsche Schmerzgesellschaft e.V.

Pat-ID: ________________

**Achse 6: Traumata / Belastungen in der Lebensgeschichte**			
Achsen-Zusatzkodierung:		*kodiere 1, 2, 3 oder 4*	
Bis zu 3 Auffälligkeiten markieren durch ankreuzen pro Auffälligkeit auch Zeitangabe <u>voranstellen</u><br><br>auf Doku-Beleg übertragen		1	Verlust naher Angehöriger / Bezugspersonen
		2	Konflikte innerhalb der Familie / Partnerschaft
		3	Konflikte am Arbeitsplatz
		4	Körperliche und/oder psychische Misshandlungen
		5	Verlust / Bedrohung der existenziellen Basis
		6	Akute Lebensbedrohung
		7	Harte Erziehungsbedingungen mit emotionaler Entbehrung
		8	Schwere körperl./psych. Erkrankung naher Bezugspersonen
		9	Eigene schwere körperliche / psychische Erkrankung
↑<br>Zeitzusatz-Kodierung			1 Kindheit (bis 6 Jahre)<br>2 Jugendzeit (bis 18 Jahre)<br>3 Erwachsenenalter (ab 18 Jahre)<br>4 Kindheit und Jugendzeit<br>5 Jugendzeit und Erwachsenenalter<br>6 Kindheit und Erwachsenenalter<br>7 Kindheit, Jugend und Erwachsenenalter

**Achse 7: Habituelle Personen-Merkmale**		
Achsen-Zusatzkodierung:		*kodiere 1, 2, 3 oder 4*
Bis zu 3 Auffälligkeiten markieren durch ankreuzen<br><br>auf Doku-Beleg übertragen	1	Selbstüberforderung bei exzessivem Leistungsanspruch
	2	Mangelnde soziale Kompetenz
	3	Selbstwertdefizite
	4	Starre Norm- und Wertvorstellungen
	5	Mangelnde Selbstreflexion / Introspektionsfähigkeit
	6	Mangelnde Wahrnehmung eigener Stressreaktionen
	7	Psychophysiologische Reaktionsstereotypie
	8	Abhängigkeitsverhalten

**Achse 8: Maladaptive Stressverarbeitung**		
Achsen-Zusatzkodierung:		*kodiere 1, 2, 3 oder 4*
Bis zu 3 Auffälligkeiten markieren durch ankreuzen<br><br>auf Doku-Beleg übertragen	1	Katastrophisierend-/vermeidende Stressverarbeitung
	2	Resignativ-/rückzugsbetonte Stressverarbeitung
	3	Ärger-betonte Stressverarbeitung und Kontrollillusion
	4	Mangelnde Wahrnehmung/Bagatellisierung von Stressreaktionen
	5	Übermäßige körperliche Ablenkung bei Stress
	6	Mangel an entspannungsfördernder Stressbewältigung
	7	Mangel an emotionsregulierender Stressbewältigung
	8	Mangel an sozial kompetenter Stressverarbeitung

C11

IMA
psychologische Dokumentation
inkl. MASK-P

Pat-ID: ___________________

Bitte bewerten Sie im Anschluss an das IMA folgende Aussagen und notieren Sie gerne Ihre Anmerkungen im Freitext:

**Vom Psychologen auszufüllen**	stimme voll und ganz zu	stimme eher zu	weder noch	stimme eher nicht zu	stimme überhaupt nicht zu
Es lagen relevante Vorbefunde in ausreichendem Maß vor.	☐	☐	☐	☐	☐
Es war ausreichend Zeit vorhanden, die vorliegenden Befunde zu sichten.	☐	☐	☐	☐	☐
Die Zeit für die Umsetzung des psychologischen IMA war ausreichend.	☐	☐	☐	☐	☐

Freitext:

C12

IMA
Interdisziplinäre Teamsitzung inkl. Abschlussgespräch

IMA-Termin Datum: ____________________ **Pat-ID:** _____________________

Übertrag Einzelbefunde (nur für Teamübersicht)

	Schweregrad Störungsbild	Bedeutung f. Schmerzproblematik	Sicherheit der Einschätzung	IMST-Ansatz erforderlich	Spezifische Behandlung in Fachbereich erforderlich	Gruppenfähigkeit für IMST	Kontraindikation für IMST
**ärztlich**	______	______	______	______	______	______	______
**physioth.**	______	______	______	______	______	______	______
**psychol.**	______	______	______	______	______	______	______
	0 = kein<br>1 = leicht<br>2 = mittel<br>3 = schwer	0 = keine<br>1 = gering<br>2 = moderat<br>3 = hoch	1 = gering<br>2 = mittel<br>3 = hoch	0 = nein<br>1 = fraglich<br>2 = ja	0 = nein<br>1 = fraglich<br>2 = ja	0 = nein<br>1 = fraglich<br>2 = ja	0 = nein<br>1 = fraglich.<br>2 = ja

Einschätzung Team über Ursachenmodell Patient/in:

kein Modell	rein somatisch	in Ansätzen bio-psycho-sozial	bio-psycho-sozial
☐	☐	☐	☐

Ziele Team (für den Pat.):

____

____

____

Therapieempfehlung TEAM (Bitte ankreuzen)

**Grundsätzliche Einschätzung**		
Weitergehende Diagnostik vor Therapieentscheidung notwendig	☐ Ja	☐ Nein
IMST-Ansatz erforderlich?	☐ Ja	☐ Nein
Kontraindikation für IMST gegeben (somatisch/psychologisch)?	☐ Ja	☐ Nein
Gruppenfähigkeit gegeben?	☐ Ja	☐ Nein
Vorrangig spez. Therapiebedarf (Med, Psy, PT), nicht in IMST integrierbar?	☐ Ja	☐ Nein

C12

IMA
Interdisziplinäre Teamsitzung inkl. Abschlussgespräch

Pat-ID: ____________________

**Konkrete Empfehlung** (Mehrfachangaben möglich)	**Empfehlung TEAM**	**Empfehlung nach Abschlussgespräch**
Akut-stationäre Einrichtung (Prüfung einer OP-Indikation)		
Weiterführung der bestehenden hausärztlichen Behandlung ist ausreichend		
Weiterführung der bestehenden fachärztlichen Behandlung ist ausreichend		
Neue ambulante fachärztliche Behandlung mit Angabe der Fachrichtung		
Anpassung der medikamentösen Therapie		
Weiterführung oder Ausbau der alltäglichen körperlichen Aktivität		
Funktionstraining/Rehasport		
Physiotherapie, Ergotherapie oder Logopädie mit Angabe PT/ET/LP		
Ambulante Psychotherapie		
Psychosomatische Einrichtung		
Ambulante oder stationäre Reha-Behandlung		
Edukative IMST (E-IMST)		
Begleitende IMST (B-IMST) Keine Kombination mit anderen neuen Verfahren der Regelversorgung!		
(Teil-)stationäre IMST (im Rahmen der Regelversorgung)		
Sonstiges		

Bitte denken Sie daran, die Diagnosen inkl. möglicher weiterer somatischer sowie psychologischer / psychiatrischer Diagnosen im Team zu besprechen und in C5 einzutragen!

Abschlussgespräch mit Patient/in

Ziele Patient/in:

____

____

C12

IMA
Interdisziplinäre Teamsitzung inkl. Abschlussgespräch

Pat-ID: ____________________

Begründung im Fall von Abweichungen von der Empfehlung

(bitte auch in Tabelle Therapieempfehlung TEAM notieren):

□ Pat. lehnt Therapieempfehlung ab (z.B. kein Interesse, lehnt Therapiekonzept ab).
□ Pat. lehnt aus organisatorischen Gründen die Therapieempfehlung ab.
□ Sonstiges: ____

Dokumentation Befundbericht

Befundbericht verschickt	Datum:

Bitte bewerten Sie im Team im Anschluss an das Abschlussgespräch folgende Aussagen. Sollte eine interdisziplinäre Einigung nicht möglich sein, kreuzen Sie nichts an.

**Vom Team auszufüllen**	stimme voll und ganz zu	stimme eher zu	weder noch	stimme eher nicht zu	stimme überhaupt nicht zu
Im Team bestand Konsens in Bezug auf die Therapieempfehlungen.	□	□	□	□	□
Es war leicht mit Patienten ein gemeinsames Ziel zu definieren.	□	□	□	□	□
Die Zeit für die Umsetzung der Teambesprechung / des Abschlussgesprächs war ausreichend.	□	□	□	□	□
Freitext:					

C13

PAIN2020

IMA Algorithmus

Deutsche Schmerzgesellschaft e.V.

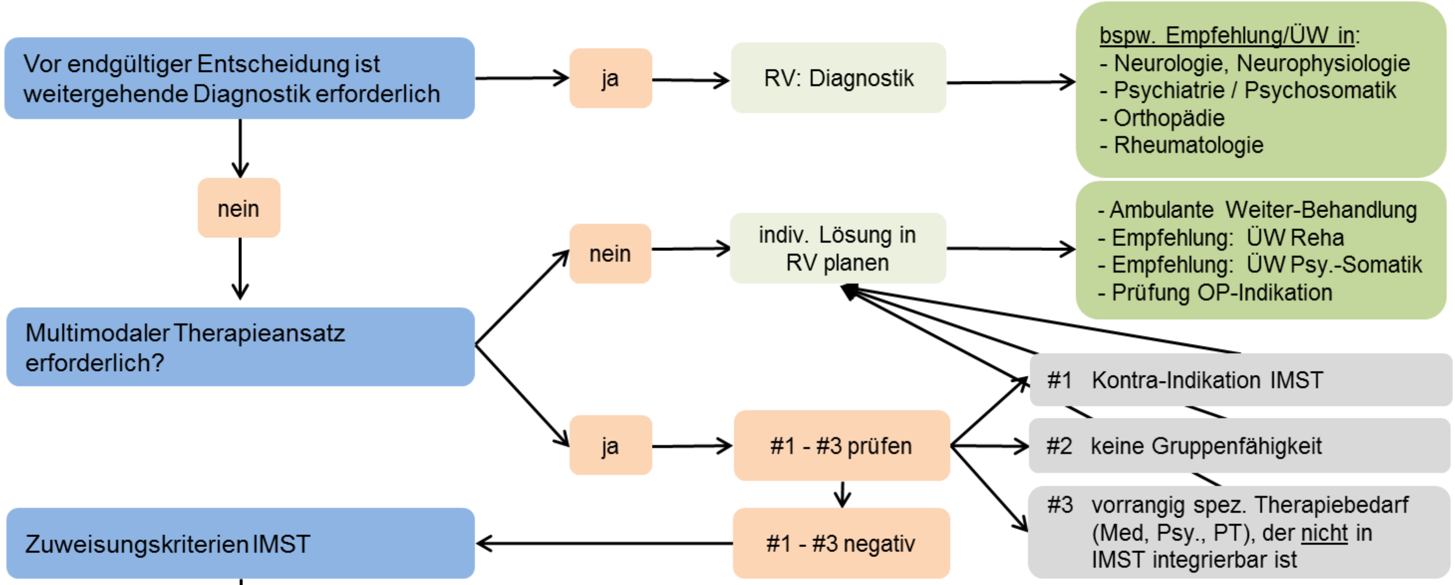

	IMST im Rahmen der Studie		RV
	E-IMST	B-IMST	T/S-IMST
MPSS-Chronifizierung (I-III)	I / II	I / II	II / III
v.Korff-Graduierung (1-4)	bis 2	bis 4	3 und 4
Komplexität der Störung<br>Einfluss-/Risikofaktoren auf allen Ebenen des bio-psycho-sozialen Schmerzmodells	gering	hoch	hoch
Regelmäßige Einzeltherapie PT	möglich*	nein, ÜW in RV	integriert
Regelmäßige Einzeltherapie PSY	möglich*	nein, ÜW in RV	integriert
Regelmäßige Einzeltherapie Med	möglich*	nein, ÜW in RV	integriert

RV: Regelversorgung
ÜW: Überweisung
ET: Einzeltherapie
PT: Physiotherapie
Psy: Psychotherapie
Med: Medizin. Behandlung
* Ergänzende, parallele Einzeltherapie in der RV bei positiver klinischer Beurteilung
